# Self-alleviation of continuous-cropping obstacles in potato via root-exudate-driven recruitment of growth-promoting bacteria

**DOI:** 10.1016/j.xplc.2025.101372

**Published:** 2025-05-12

**Authors:** Haiyan Ma, Zhitong Ren, Aihua Luo, Xiaoting Fang, Ruilin Liu, Chao Wu, Xinxin Shi, Junji Li, Heping Lv, Xiaohua Sun, Kaiqin Zhang, Shunlin Zheng

**Affiliations:** 1 State Key Laboratory of Crop Gene Exploration and Utilization in Southwest China, College of Agronomy, Sichuan Agricultural University, Chengdu 611130, China; 2 Crop Ecophysiology and Cultivation Key Laboratory of Sichuan Province, Chengdu 611130, China; 3 Potato Research Institute of Gansu Academy of Agricultural Sciences, Lanzhou 730070, China; 4 Key Laboratory of Tuber Crop Genetics and Breeding, Ministry of Agriculture, Chengdu Joyson Agricultural Technology Co., Ltd, Xindu 610500, China; 5 Yibin Academy of Agricultural Sciences, Yibin 644699, China

**Keywords:** potato, continuous cropping, *Pantoea* sp. MCC16, nobiletin, adventitious root numbers

## Abstract

Continuous-cropping (CC) obstacles are among the primary factors limiting the development of global agriculture. Although most plants are negatively affected by barriers that develop with CC, they may also overcome such obstacles by altering the soil biological and chemical environment to favor plant growth. In this study, we investigated the mechanism by which plants recruit beneficial microorganisms using root exudates to alleviate obstacles in a 10-year CC potato system. On day 20 after potato emergence, soil microorganisms in the CC system promoted an increase in adventitious root (AR) numbers by increasing the indole-3-acetic acid (IAA) content of the rhizosphere soil. Analysis of rhizosphere bacterial communities using 16S rRNA sequencing revealed that CC alters community structure, increasing the abundance of *Pantoea* sp. MCC16. Irrigation with root exudates from CC potato significantly increased AR numbers and *Pantoea* sp. MCC16 abundance. Through untargeted metabolomic analysis, we identified nobiletin as key metabolite that promotes *Pantoea* sp. MCC16 colonization in the rhizosphere. Furthermore, application of either nobiletin or *Pantoea* sp. MCC16 significantly improved the yield of CC potatoes. These findings demonstrate that CC plants can actively secrete the unique metabolite nobiletin to recruit *Pantoea* sp. MCC16, a high IAA producer, to help plants recover functional traits and mitigate CC obstacles.

## Teaser

Potato exhibits autonomous adaptation to continuous-cropping stress. This study provides evidence that continuously cropped potato secretes nobiletin to recruit *Pantoea* sp. MCC16, which increases rhizosphere IAA levels and stimulates adventitious root proliferation during the seedling stage, thereby alleviating the decline in root biomass and yield caused by self-toxic stress.

## Introduction

Although the traditional practice of continuous cropping (CC) can meet short-term yield demands, the prolonged use of CC engenders a complex of persistent challenges, collectively termed CC obstacles. CC obstacles significantly impair crop productivity and sustainability, thereby hindering the long-term development of modern agriculture (Li et al., 2021; Ma et al., 2023a).

Geographic constraints, arable-land limitations, economic demands, and intensified modern farming practices reduce species diversity in continuously cropped areas. Combined with excessive fertilizer application aimed at maximizing crop yields, these factors disrupt ecosystem self-regulation, exacerbating CC obstacles. It is challenging to completely eliminate such obstacles when implementing a CC system. However, artificial means can be used to mitigate the suppression of plant growth in CC systems. The main obstacles to CC are usually caused by soil-borne diseases and autotoxicity. Approaches to prevent and control soil-borne diseases in CC systems have evolved from extensive management aimed at eliminating all soil microorganisms, to more refined management focusing on individual pathogenic bacteria, and finally to management of the entire microbial community, which involves making overall adjustments to soil structure (Wang et al., 2024a). The introduction of microorganisms with specific functions has historically been the primary technique used to regulate soil microbial community structure. However, soil colonization is challenging because of factors such as poor environmental adaptability, competition with native flora, and host immune responses. Therefore, an effective strategy to prevent and control soil-borne diseases under CC conditions is the direct soil application of prebiotics required by beneficial bacteria to increase their competitiveness or the application of functional strains that can inhibit the main active substances of pathogenic bacteria (Chung et al., 2016; Wen et al., 2023). In addition, ecosystem function can be effectively optimized by leveraging the “home-field advantage” of native beneficial microorganisms (Jiang et al., 2023).

Autotoxicity, mainly mediated by phenolic acids produced by the plants themselves, remains a difficult problem. Small amounts of phenolic acids can temporarily improve plant tolerance and reduce oxidative stress (Martinez et al., 2016; Sun et al., 2024). However, long-term CC leads to the accumulation of large amounts of phenolic acids in the soil, which disrupt root membrane structures and hinder the development of root morphology (Ni et al., 2021). Autotoxicity is most commonly observed during the seedling stage, although the specific phenolic acids responsible for autotoxicity vary among different plants (Wang et al., 2022). Therefore, to alleviate autotoxicity, in addition to developing new varieties that are resistant to CC obstacles, a common practice is to screen for beneficial bacteria that can degrade specific phenolic acids on a large scale and then apply them to CC soil in a targeted manner (Chen et al., 2023). However, with this method, challenges remain in terms of colonization and efficacy. Artificial stimulation of root system establishment during the seedling stage is another approach to alleviate autotoxicity, and it has been shown to increase yield by approximately 20% (Ma et al., 2023b). With this method, negative plant responses to external stress weaken, gradually reducing the release of phenolic acids into the soil.

Although CC leads to an imbalance in overall ecosystem function, plants can still promote their own growth by adjusting surrounding microhabitats in a “cry-for-help” strategy that is common in plant–microbe systems (Rolfe et al., 2019). Plants actively secrete metabolites that attract beneficial bacteria to accumulate in the rhizosphere. The specific metabolites serve as signaling or energy substances for the bacteria, with specific bacteria selectively recruited during certain periods and their functions maintained. Under long-term stress conditions, nonresistant plant varieties tend to be more effective at stimulating soil microbial communities to suppress pathogens and alleviate environmental stresses—a phenomenon indicative of the development of resistant soil—compared with their resistant counterparts, although the extent of this effect varies depending on the specific environmental conditions and the particular plant–microbe interactions involved (Raaijmakers and Mazzola, 2016). For example, susceptible varieties of cucumber secrete more organic acids to recruit Comamonadaceae, which inhibit *Fusarium oxysporum* f.sp. *cucumerinum* (Foc) (Wen et al., 2020). Similarly, after infection by *Pseudomonas syringae* pv *tomato* (Pst), the subsequent generation of *Arabidopsis* secretes more long-chain organic acids and amino acids to regulate the soil bacterial community and increase plant resistance (Yuan et al., 2018). Some of the obstacles posed by CC can be alleviated or even eliminated with time, usually more than 10 years (Zhang and Chen, 2014), suggesting that plants develop strategies to cope with CC obstacles over the long term. Such strategies are closely linked to the “root exudate–soil microbial” system. Therefore, harnessing the restoration capabilities of the ecosystem itself may be the most effective approach for mitigation of CC obstacles.

Potatoes, a top-four food crop, are known for their nutrient-rich and stress-resistant properties and are widely grown in CC systems. However, the inherent intolerance of Solanaceae plants to CC makes the CC of potato a persistent industry challenge (Chen et al., 2023), and potato seedling growth is typically significantly inhibited in CC systems. In the absence of noticeable pest and disease symptoms, the accumulation of autotoxic substances in soil is thought to be a primary factor underlying this phenomenon (Guo et al., 2016; Lyu et al., 2022; Xiang et al., 2022). In previous research, we discovered that the autotoxic substance vanillin, present in CC systems, initially led to significantly lower adventitious root (AR) numbers than those of normal plants (Ma et al., 2023b). However, AR numbers increased significantly later in the seedling stage. We hypothesized that long-term CC stimulates plants to autonomously recruit beneficial microorganisms, thereby increasing AR numbers. Changes in potato root exudates are considered to drive increases in beneficial plant microorganisms, with exudates influenced by autotoxic substances in CC soil. To test this hypothesis, we performed experiments to (1) evaluate the establishment of a microbiome that promotes increased AR numbers in a CC system, (2) analyze root exudates of CC potatoes to identify signaling compounds for the recruitment of beneficial bacterial taxa, and (3) examine changes in plant-growth-promoting rhizosphere bacteria after exposure to root exudates from CC potatoes and examine the effect of using plant-growth-promoting bacteria and growth-promoting metabolites to alleviate CC obstacles (Supplemental Figure 1).

The results of this study shows that AR numbers increased significantly at a later seedling stage under vanillin stress in CC conditions. The increase in AR numbers was due to significant enrichment of *Pantoea* sp. MCC16 in the rhizosphere soil. By increasing the indole-3-acetic acid (IAA) content of rhizosphere soil, the bacterium increased the content of root IAA, which in turn promoted increases in AR. In addition, we found that enrichment of *Pantoea* sp. MCC16 was due to the recruitment ability of nobiletin, which was secreted by CC potato roots under autotoxic conditions.

## Results

### Autotoxicity inhibits potato plant growth under continuous cropping

**Figure 1:**
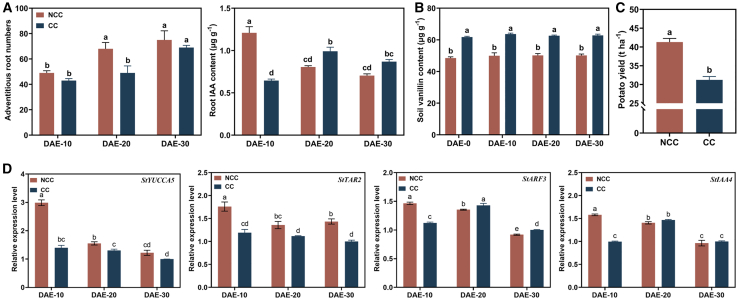
Inhibition of potato plant growth under continuous cropping. **(A)** Effects of continuous cropping (CC) on the number of potato ARs and the root indole-3-acetic acid (IAA) content. **(B)** Effects of CC on soil vanillin content. **(C)** Effects of CC on potato yield. **(D)** Effects of CC on the expression of IAA-synthesis (*StYUCCA5*, *StTAR2*) and IAA-response (*StARF3*, *StIAA4*) genes. NCC, noncontinuous cropping; DAE-X, X days after potato emergence. Data are shown as the mean ± SEM (*n* = 3). Different letters indicate significant differences (Tukey’s HSD test, *p* < 0.05).

CC significantly inhibited potato seedling growth (Figure 1, ① in Supplemental Figure 1). At the seedling stage, AR numbers increased significantly at 10–20 days after emergence (DAE) under noncontinuous cropping (NCC) conditions but at 20–30 DAE under CC conditions. The maximum IAA content occurred at 10 DAE in NCC potato roots but at 20 DAE in CC potato roots (Figure 1A). Although plant height, shoot diameter, and leaf and root dry weights of CC potato increased significantly at 20–30 DAE, these values were always lower than those of NCC potato, and potato yield was significantly lower (−24.43%) under CC conditions than under NCC conditions (Supplemental Figure 2; Figure 1C). In rhizosphere soil at the seedling stage, the vanillin content of CC soil was significantly higher than that of NCC soil on all sample dates (Figure 1B). According to RT–qPCR analysis, CC significantly inhibited expression of the root IAA-synthesis genes *StYUCCA5* and *StTAR2* throughout the seedling stage, whereas expression of the root IAA-response genes *StARF3* and *StIAA4* increased significantly at 10–20 DAE (Figure 1D). These results indicated that CC significantly inhibited the growth of potato seedlings but that substances in CC soil promoted an increase in root IAA content at 20 DAE.

### Soil microorganisms promotes potato plant growth under continuous cropping

**Figure 2:**
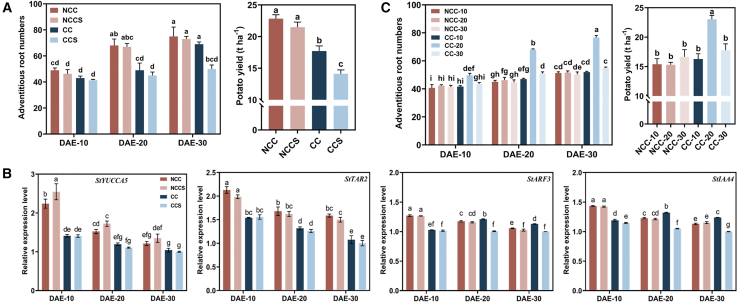
Rhizosphere microorganisms promote potato plant growth in continuously cropped soil 20 days after emergence. **(A)** Effects of soil sterilization on the AR number and yield of potato. NCC, unsterilized noncontinuous-cropping soil; NCCS, sterilized noncontinuous-cropping soil; CC, unsterilized continuous-cropping soil; CCS, sterilized continuous cropping soil; DAE-X, X days after potato emergence. **(B)** Effects of soil sterilization on the expression of IAA-synthesis (*StYUCCA5*, *StTAR2*) and IAA-response (*StARF3*, *StIAA4*) genes in potato roots. **(C)** Effects of microflora transfer on AR numbers and potato yield. NCC-X, rhizosphere microorganisms on day X after potato emergence in noncontinuous cropping soils; CC-X, rhizosphere microorganisms on day X after potato emergence in CC soils. Data are shown as the mean ± SEM (*n* = 3). Different letters indicate significant differences (Tukey’s HSD test, *p* < 0.05).

To determine whether microorganisms in CC soil could promote plant growth, we set up a soil sterilization test. In CC soil, the presence of microorganisms promoted potato plant growth (Supplemental Figure 3A). Soil vanillin content was significantly higher in CC soil than in NCC soil, regardless of whether the CC soil had been sterilized, indicating the development of autotoxicity in CC soil (Supplemental Figure 3D). Soil sterilization had no significant effect on AR numbers at any seedling stage in NCC soil, and AR numbers increased significantly at 10–20 DAE (Figure 2A). In addition, IAA content did not differ significantly between sterilized and unsterilized NCC soil during the seedling stage, and root IAA content was highest at 10 DAE (Supplemental Figures 3B and 3C).

In CC soil, AR numbers in sterilized soil did not increase significantly during the seedling stage, but in unsterilized soil, numbers increased significantly at 20–30 DAE (Figure 2A). In CC rhizosphere soil, IAA content increased significantly at 20 DAE (Supplemental Figure 3C). Notably, expression of the root IAA-synthesis genes *StYUCCA5* and *StTAR2* decreased significantly, whereas expression of the root IAA-response genes *StARF3* and *StIAA4* increased significantly (Figure 2B). In addition, although potato yield was significantly lower in CC soil than in NCC soil (regardless of sterilization), potato yield was significantly lower in sterilized continuously cropped soil (CCS) than in in unsterilized CC soil (Figure 2A). These results indicated that soil microorganisms caused the increase in AR numbers under CC conditions (with autotoxicity) (② in Supplemental Figure 1).

To further explore the stage at which microorganisms in CC soil played the main role in increasing AR numbers, we collected rhizosphere flora of potato seedlings in CC and NCC soil and transferred them to sterilized CC soil. There was no significant difference in the effect of bacterial transfer on soil vanillin content, and vanillin stress was present in each treatment (Supplemental Figure 3G). Soil microorganisms from NCC soil did not significantly affect the IAA content of rhizosphere soil or potato roots, whereas soil microorganisms from CC soil significantly increased IAA content in both soil and roots. Regarding the increases in IAA content in rhizosphere soil and roots, the most significant effect of CC rhizosphere microorganisms occurred at 20 DAE (Supplemental Figure 3E and 3F).

In addition, microorganisms from the rhizosphere of NCC potato seedlings produced no significant increases in AR numbers or potato yield. However, microorganisms from CC soil at 20 DAE significantly increased AR numbers throughout the seedling stage, with the first significant difference detected at 10 DAE (Figure 2C). Therefore, microorganisms from CC soil at 20 DAE significantly increased the yield of CC potatoes (Figure 2C, ③ in Supplemental Figure 1). On the basis of these results, we hypothesized that microorganisms in CC soil could significantly increase potato yield under CC vanillin stress, with the main functions of rhizosphere microorganisms apparent at 20 DAE of CC potatoes.

### *Pantoea* sp. MCC16 promotes potato growth under continuous cropping

After confirming that rhizosphere soil microorganisms at 20 DAE had a significant growth-promoting effect on CC potatoes, we analyzed the bacterial community structure of CC and NCC soil by performing sequencing for absolute quantification of 16S rRNA at 10 and 20 DAE.

**Figure 3:**
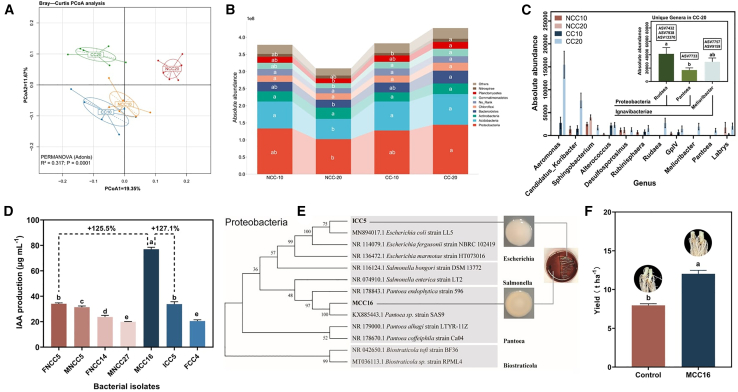
*Pantoea* sp. MCC16 promotes plant growth in continuous cropping soil. **(A)** Principal coordinate analysis of differences in rhizosphere soil community structure under continuous cropping (CC) and noncontinuous cropping (NCC) conditions on days 10 and 20 after emergence. NCC-X, rhizosphere microorganisms on day X after potato emergence in NCC soils; CC-X, rhizosphere microorganisms on day X after potato emergence in CC soils. **(B)** Absolute abundance of the top 10 bacterial phyla in CC10, CC20, NCC10, and NCC20. **(C)** Bacterial genera absent (11) in CC10, NCC10, or NCC20 and unique genera (3) in CC20. **(D)** Strains with an indole-3-acetic acid (IAA) production capacity >20 μg ml^−1^ in CC and NCC rhizosphere soils. I-X, 10 days after potato emergence (DAE); M-X, 20 DAE; F-X, 30 DAE. **(E)** Evolutionary tree of bacterial species ICC5 and MCC16. The red plate contains *E. coli* identification medium. **(F)** Effect of irrigation with a solution of *Pantoea* sp. MCC16 on the yield of CC potato. Data are shown as the mean ± SEM (*n* = 3). Different letters indicate significant differences (Tukey’s HSD test, *p* < 0.05).

The difference in rhizosphere bacterial community structure between CC and NCC was more significant at 20 DAE than at 10 DAE (Figure 3A). Bacterial community diversity was significantly higher in CC soil than in NCC soil, especially at 20 DAE, whereas there was little difference in community richness (Supplemental Figure 4A and 4B). The absolute abundance of the top 10 bacterial phyla was highest in CC soil at 20 DAE (CC20), especially for Proteobacteria and Bacteroidetes (Figure 3B). To identify which bacteria in CC20 caused significant differences compared with the other treatments, we identified the bacteria that were present in CC20 but not in CC10, NCC10, or NCC20. Eleven bacteria were screened in total, and *Rudaea* (3ASV), *Pantoea* (1ASV), and *Melioribacter* (2ASV) were present only in the CC20 treatment; *Rudaea* and *Pantoea* are members of Proteobacteria (Figure 3C).

Because previous experiments confirmed that the bacteria in CC20 rhizosphere soil could increase soil IAA content, we isolated and cultured rhizosphere soil bacteria in the seedling stage under CC and NCC. Seven IAA-producing strains (IAA-producing ability >20 μg ml^−1^) were isolated: three strains in CC and four strains in NCC. The IAA production capacity of MCC16 in CC was as high as 77.022 μg ml^−1^, which was 125.5% higher than that of FNCC5, the strain with the highest IAA production capacity in NCC (Figure 3D). The seven strains were then identified by analyzing their 16S rDNA sequences. Five of the strains were in Firmicutes, whereas the other two were in Proteobacteria (ICC5, MCC16), and the abundance of Proteobacteria in CC20 increased significantly (Figure 3B and 3D). Strain ICC5 was identified as *Escherichia coli*, and strain MCC16 showed 100% homology to *Pantoea* sp. SAS9 (Figure 3E). Absolute quantification of 16S rRNA sequencing results revealed that only one taxon, ASV7733, was in the genus *Pantoea*. This taxon was only found at 20 DAE, and strain MCC16 was isolated from potatoes at 20 DAE, indicating that ASV7733 was the MCC16 strain (Figure 3E; Supplemental Figure 4C, ④ in Supplemental Figure 1).

When determining whether *Pantoea* sp. MCC16 could promote plant growth in CC potato, we found that the IAA content in rhizosphere soil and roots of CC potato increased significantly after bacterial suspension treatment, leading to increases in AR numbers and potato yield. Notably, expression of the IAA-response genes *StARF3* and *StIAA4* was significantly enhanced in the roots after *Pantoea* sp. MCC16 treatment, whereas expression of the IAA-synthesis genes *StYUCCA5* and *StTAR2* showed no significant change (Figure 3F; Supplemental Figure 4D and 4E). These results also confirmed that *Pantoea* sp. MCC16 was the main strain that promoted plant growth in CC soil (⑤ in Supplemental Figure 1).

### Potato root exudates under continuous cropping promote plant growth

**Figure 4:**
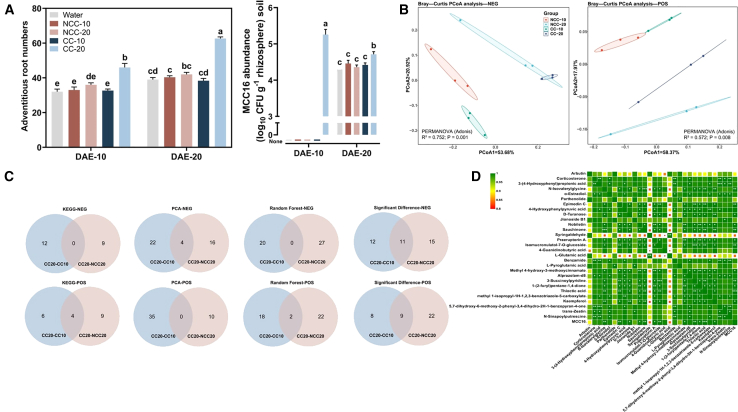
*Pantoea* sp. MCC16 colonization was stimulated by root exudates on day 20 after emergence of continuously cropped potato. **(A)** Effects of root exudates from CC and NCC potato on AR numbers and *Pantoea* sp. MCC16 abundance of CC potato. NCC-X, root exudates on day X after potato emergence in NCC soils; CC-X, root exudates on day X after potato emergence in CC soils; CFU, colony-forming unit. **(B)** Principal coordinate analysis of root exudates in CC and NCC. **(C)** Four methods used to screen for metabolites that were concentrated in the root exudates of CC20. **(D)** Correlation analysis of metabolites enriched in CC20 (29 metabolites) and *Pantoea* sp. MCC16. Data are shown as the mean ± SEM (*n* = 3). Different letters indicate significant differences (Tukey’s HSD test, *p* < 0.05).

To determine why *Pantoea* sp. MCC16 appeared only in CC20, we collected root exudates of CC and NCC potatoes at 10 and 20 DAE and performed irrigation tests of root exudates against the background of unsterilized CC soil. Only the CC20 root exudates significantly increased AR numbers at the beginning of the potato seedling stage and maintained this enhancement effect over time. In addition, strain MCC16 in rhizosphere soil appeared only under irrigation with CC20 root exudates at 10 DAE (Figure 4A). These results indicated that CC20 root exudates could directly promote *Pantoea* sp. MCC16 colonization of the potato rhizosphere (⑥ in Supplemental Figure 1).

To further explore which specific metabolites in CC20 could recruit *Pantoea* sp. MCC16, we performed untargeted metabolomics analysis of root exudates in CC and NCC. The components and contents of root exudates treated with CC and NCC differed significantly in both positive and negative modes (Figure 4B). Using Kyoto Encyclopedia of Genes and Genomes (KEGG) pathway enrichment analysis (TOP10) (Supplemental Figure 5; Supplementary Table 3), principal-component analysis variable-importance analysis (TOP40) (Supplemental Figure 6), random forest variable importance analysis (TOP40) (Supplemental Figure 7), and significant difference analysis (TOP50) (Supplemental Figure 8), we selected 29 metabolites that increased significantly only in CC20 (Figure 4C, ⑦ in Supplemental Figure 1). The absolute abundance of MCC16 was significantly positively correlated with the relative contents of 11 metabolites: N-isovalerylglycine, epimedin C, D-turanose, nobiletin, sauchinone, praeruptorin A, isomucronulatol-7-O-glucoside, methyl 4-hydroxy-3-methoxycinnamate, 3-succinoylpyridine, thioctic acid, and kaempferol (Figure 4D).

### Nobiletin alleviates potato continuous cropping obstacles by recruiting *Pantoea* sp. MCC16

**Figure 5:**
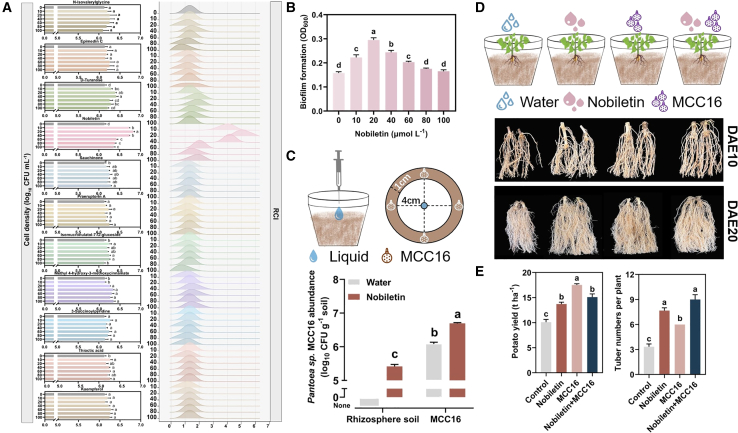
Validation of the ability of nobiletin to alleviate continuous cropping obstacles in potato. **(A)** Chemotactic analysis of 11 different concentrations of metabolites and *Pantoea* sp. MCC16. **(B)** Effect of different concentrations of nobiletin on the biofilm formation of *Pantoea* sp. MCC16. **(C)** Verification of the chemotaxis of *Pantoea sp. MCC16 toward* exogenous metabolites in sterilized CC soil. “Rhizosphere soil” denotes rhizosphere soil inoculated with CC20; “MCC16” denotes inoculation with *Pantoea* sp. MCC16. **(D)** Effects of exogenous nobiletin and *Pantoea* sp. MCC16 on the growth of CC potato. Different droplet properties represent different treatments. The blue droplet represents the control with sterile water. DAE, days after emergence. **(E)** Effects of exogenous nobiletin and *Pantoea* sp. MCC16 on the yield of CC potato. Data are shown as the mean ± SEM (*n* = 3). Different letters indicate significant differences (Tukey’s HSD test, *p* < 0.05).

To determine which substances could recruit *Pantoea* sp. MCC16, we performed an *in vitro* test to assess the chemotaxis of *Pantoea* sp. MCC16 toward the 11 substances. Only nobiletin significantly stimulated chemotaxis in *Pantoea* sp. MCC16, and this chemotaxis reached its maximum value at 20 μmol l^−1^ nobiletin (Figure 5A). We next examined the effect of nobiletin on biofilm formation by *Pantoea* sp. MCC16 and found that biofilm formation was significantly promoted by 20 μmol L^−1^ nobiletin (Figure 5B, ⑧ in Supplemental Figure 1). To confirm this result, we set up an experiment with exogenous nobiletin irrigation in CC soil. In CCS, exogenous nobiletin had little effect on AR numbers. In unsterilized CC soil, however, nobiletin significantly promoted AR numbers, increasing them by 73.91% at DAE-10 and 125.49% at DAE-20 compared with CCS (Supplemental Figure 9). Finally, we set up an experiment with exogenous nobiletin irrigation in CC soil. Compared with CC or CCS control soil, nobiletin significantly increased the abundance of *Pantoea* sp. MCC16 in rhizosphere soil. These results suggested that nobiletin was a major functional substance secreted by CC potato roots that could recruit *Pantoea* sp. MCC16 (Figure 5C, ⑨ in Supplemental Figure 1).

Finally, we performed a pot test with exogenous application of nobiletin and a *Pantoea* sp. MCC16 bacterial solution. Nobiletin and *Pantoea* sp. MCC16 significantly increased AR numbers at 10 and 20 DAE (Figure 5D). In further studies, no significant difference was found in soil vanillin content under different treatments. However, the IAA content of potato rhizosphere soil and roots increased significantly (Supplemental Figure 10). Exogenous application of nobiletin and *Pantoea* sp. MCC16 also significantly increased potato yield, although their modes of action differed. Nobiletin mainly increased potato quantity, whereas *Pantoea* sp. MCC16 mainly increased potato unit weight (Figure 5E, ⑩ in Supplemental Figure 1; Supplemental Figure 12). This difference may have occurred because exogenous nobiletin changed the structure of the rhizosphere microbial community, thus changing microbial community function.

## Discussion

**Figure 6:**
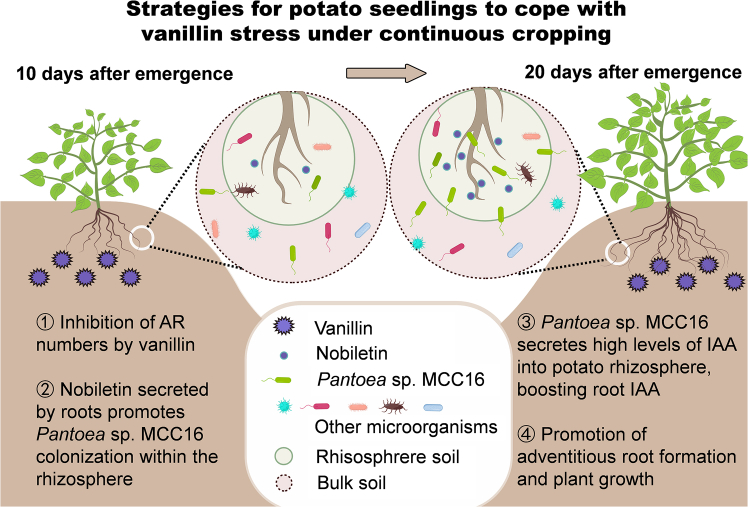
Conceptual model of plant self-repair under continuous cropping obstacles. (1) When potatoes are replanted into continuous cropping (CC) potato soil with accumulated vanillin, seedlings suffer vanillin stress, resulting in inhibited IAA synthesis in the roots, lower adventitious root (AR) numbers, and suppressed aboveground growth. (2) Under CC vanillin stress, potato plants adopt autonomous adaptive strategies by secreting substantial amounts of nobiletin from roots, which acts as a signaling molecule to recruit *Pantoea* sp. MCC16 for rhizosphere enrichment. (3) In the CC potato rhizosphere, *Pantoea* sp. MCC16 secretes abundant IAA (indole-3-acetic acid), increasing rhizosphere IAA levels and subsequently enhancing IAA accumulation in the roots. (4) Consequently, under vanillin stress, CC potato seedlings exhibit increased AR numbers, promoting aboveground plant growth and ultimately achieving mitigation of CC obstacles.

Agricultural ecosystems possess an inherent capacity for self-repair, which manifests as the alleviation of growth suppression caused by biotic or abiotic stresses when environmental conditions improve (Ma et al., 2019; Davis et al., 2023). However, the ability of agricultural ecosystems to overcome CC obstacles has not yet been investigated extensively. This failure may be due to plant–soil systems not reaching self-repair thresholds after an insufficient number of CC years (Xuan et al., 2024). In addition, the initial self-repair of plant–soil systems cannot compensate for losses in the final economic yield, leading to the misconception that CC systems lack self-repair ability (Wang et al., 2024a). In this study, even though the final yield of CC potato was significantly lower than that of NCC potato, the roots of CC potato actively recruited beneficial bacteria during the seedling stage to increase rhizosphere IAA content, thereby alleviating the decline in root biomass caused by self-toxic stress and further promoting the morphogenesis of CC potato (Figure 6). Previous studies on CC obstacles have focused primarily on healthy plants that do not show CC obstacles in a small number of fields (Song et al., 2021; Sui et al., 2023). In this study, our findings suggest that the CC system leads to positive regulation by the majority of plants experiencing CC obstacles. Given the prevalence of these plants, the findings of this study may have significant implications for artificial regulation strategies aimed at enhancing plant adaptation to CC environments. In addition, the results revealed a new mechanism by which CC plants autonomously recruit plant-growth-promoting bacteria to alleviate CC obstacles. Thus, this study demonstrates that beneficial microorganisms can be obtained from rhizosphere soil with CC obstacles, providing a new way to overcome difficulties due to CC.

### Self-repair in a continuous cropping system dose not directly reduce the content of an autotoxic substance

The accumulation of autotoxic substances is the main factor that causes autotoxicity during CC of plants. To date, most research on mitigating plant autotoxicity has focused on how to eliminate autotoxic substances (Zhang et al., 2010; Chen et al., 2011; Liu et al., 2023). However, according to the results of this study, the ultimate goal of the measures taken by potato plants to deal with autotoxic stress was not to reduce the content of autotoxic substances but to compensate for the plant growth defects caused by autotoxic stress, mainly by increasing AR numbers to promote plant growth at the seedling stage (Figure 1). This strategy may be a sustainable positive regulatory measure chosen by plants independently under long-term autotoxic stress to obtain greater stability. A recent study also demonstrated for the first time that continued allelopathic stress can promote plant microorganisms that have positive effects on plant performance (Revillini et al., 2023). Research also indicates that nonresistant varieties that have been exposed to the same stress for an extended period are more likely to induce soil resistance to the stress, resulting in what is commonly referred to as resistant soil (Raaijmakers and Mazzola, 2016). In addition, when a plant chooses to alleviate its own stress by reducing autotoxic substances in the soil environment, the plant’s continued input of those substances into the soil is not affected. Moreover, relying on microorganisms to degrade autotoxic substances over the long term will continue to consume the plant’s energy. By contrast, improving the plant’s own growth under autotoxic stress can adjust metabolism in the short term, thereby reducing the input of autotoxic substances into the soil and stably improving the soil environment under CC (Xuan et al., 2024).

Under vanillin stress during CC, a decrease in AR numbers due to vanillin inhibition of root IAA content is the main factor that causes reduced root biomass (Ma et al., 2023b). However, under continued vanillin stress in the present study, the IAA content of potato roots increased, the expression of root IAA-responsive genes increased significantly, and the expression of root IAA-synthesis genes decreased significantly (Figure 1D). The results indicated that the significant increase in IAA content in CC potato roots was closely associated with increased IAA content in the soil. Li et al. (2019) observed a significant decrease in the expression of IAA-synthesis and -response genes in the roots of CC peanuts. In this study, this decrease may have been attributable to the relatively short duration of CC, during which the system did not reach the threshold at which plants initiate self-repair. Furthermore, although CC potatoes did exhibit self-repair, the increase in AR numbers was delayed by approximately 10 days compared with that of normal plants (Figure 1A; Supplemental Figure 2A). This may have been because, after potato seedlings are stressed by vanillin, they require some time to respond to the recruitment of specific functional microorganisms. Previous studies have also shown that, in addition to the perception of stress signals, the initiation of *in vivo* signal transduction, and the activation of molecular network cascades of gene expression (Yang et al., 2023), plant adaptation to environmental stress also requires host–microbe interaction signaling (Rodriguez et al., 2019; Song et al., 2021). Although insufficient AR numbers in the early stage may be the main reason why the yield of CC potatoes is significantly lower than that of normal plants (Huang et al., 2017), AR numbers are expected to rise as the degree of self-repair gradually increases.

### Change in the rhizosphere microbial community promotes root morphogenesis of continuous cropping plants

Rhizosphere microorganisms have vital roles in plant growth, participating in growth and development, resistance to stress, and other processes (Berendsen et al., 2012; Kwak et al., 2018; Zhang et al., 2019). The roles of microorganisms in plant growth extend beyond the current growing season and also help future generations of plants to adjust to the growth environment (Wilkinson, 1997; Wilkinson and Sherratt, 2001; Bakker et al., 2018). In plants that reproduce vegetatively, progeny also receive a considerable proportion of symbiotic bacteria and fungi from the parent plant (Vannier et al., 2018). However, although host genetics can significantly influence the assembly of rhizosphere microbial communities, these effects are weaker than those of environmental factors (Pérez-Jaramillo et al., 2017; Thiergart et al., 2019). In addition, unlike other vegetatively propagated plants, the seed potatoes planted each year are virus free and propagated from tissue-culture seedlings. With this approach, it is not possible to pass the stress memory of potato CC obstacles to offspring. Thus, the most effective strategy for alleviating the CC obstacles encountered during potato cultivation is to develop soil resistance to CC through sustained planting practices. This process enables the soil to acquire resilience in the face of environmental changes, resulting in the formation of resistant soil.

In current agricultural research, greater attention is given to the development of resistant soil formed through the persistent induction of a specific pathogen, in which the soil acquires a strong resistance to that pathogen. This acquisition proceeds from no resistance to significant resistance and is due primarily to significant enrichment of antagonistic bacteria with specific resistance to the pathogen in the soil (Song et al., 2021). However, production problems caused by non-pathogenic factors can also lead to the formation of resistant soil, such as the enhancement of soil recovery capacity by long-term drought stress through changes in soil microbial community function (Canarini et al., 2021). Less research has focused on the induction of resistant soil by long-term CC stress. In this study, rhizosphere microorganisms from CC potatoes increased AR numbers by increasing root IAA content (Figure 2; Supplemental Figure 3). The presence of these microorganisms was likely the result of independent selection by potatoes under long-term CC conditions. Studies have shown that the microbiome selected by allelopathic substances can moderately reduce the plant growth inhibition caused by allelopathy (Revillini et al., 2023). However, the selective effect of root signals on soil microorganisms during plant growth may be stronger than that of a single allelopathic substance (Venturi and Keel, 2016). In addition, plants show dynamic changes in microbial recruitment during different growth stages (Shi et al., 2015; Cordovez et al., 2021; Santoyo, 2022). In this study, the difference in microbial communities between CC and normal potato plants was much smaller at 10 DAE than at 20 DAE. At the same time, the absolute abundance of rhizosphere microorganisms in CC potato plants increased from 10 to 20 DAE. This result was the opposite of that observed for normal potato plants (Figures 3A and 3B) and may reflect an effect of CC soil on the components and contents of potato root exudates, which in turn increased soil bacterial diversity (Supplemental Figure 4A). However, in contrast to this study, most studies have found that CC significantly reduces bacterial diversity (Tan et al., 2017; Zhao et al., 2020), a result that may be related to the different backgrounds of the plant samples. The CC barriers to plant growth in the present study were caused by autotoxicity rather than disease, which reduced the negative interactions between microorganisms to a certain extent. In addition, owing to the dynamic changes in soil microorganisms, sampling time also has an important effect on the measurement results. We believe that the seedling stage, a critical period for the establishment of plant morphology, is more important than other periods.

Plant–microbe interactions can be long term or short term (Cordovez et al., 2021; Orozco-Mosqueda and Santoyo, 2021; Santoyo, 2022). Previous studies have suggested that short-term interactions with specific probiotic bacteria may contribute to plant health and productivity (Berlanga-Clavero et al., 2020). In the present study, *Pantoea* sp. MCC16 appeared only at 20 DAE in CC potatoes (Figure 3C), indicating that there was a short-term interaction between CC potato plants and *Pantoea* sp. MCC16. This may have been because the IAA required to establish the potato root system reached its peak at the seedling stage, whereas later growth does not require abundant IAA in the soil. However, change in the abundance of *Pantoea* sp. MCC16 is a dynamic process. Although the findings of this study suggest that *Pantoea* sp. MCC16 appears only in CC20, this does not mean that no *Pantoea* sp. MCC16 is present in CC10 and CC30 but rather that the detection of low-abundance strains is limited by sequencing technology and bacterial isolation and culture methods. In addition, beneficial microorganisms can improve plant growth by secreting plant hormones into the surrounding environment and also by affecting plant gene expression to cause physiological changes (Angulo et al., 2022).

Microorganisms in the environment typically alter plant IAA levels through two mechanisms: one is the synthesis of IAA by microorganisms for direct absorption and utilization by the plant, and the other is the induction of IAA production by the plant itself (Etesami and Glick, 2024). Plant absorption of exogenous IAA can occur through diffusion or via transport proteins that facilitate its entry into the root system. The process by which microorganisms stimulate the autonomous production of IAA in plants often involves signaling pathways activated by metabolic byproducts or other compounds from the microorganisms. For instance, rhizobia AS5 and AS55 stimulate the expression of *IAA12* and *YUCCA2* in *Arabidopsis*, leading to an increase in endogenous IAA levels (Sujkowska-Rybkowska et al., 2022). *Lysinibacillus* spp. can promote plant growth by producing IAA and influencing the plant’s own IAA production (Pantoja-Guerra et al., 2023). Our previous research found that exogenous IAA can enhance the IAA level of potato roots under vanillin stress in a sterile matrix, demonstrating that the root can use exogenous IAA directly (Ma et al., 2023b). In the present study, the rhizosphere microorganisms of CC potato did not affect the expression of auxin synthesis genes in potato roots under autotoxic stress but did significantly increase the expression of auxin-response genes (Figure 2B), indicating that the increased AR numbers may have been due to direct utilization of IAA produced by MCC16 in the soil. Given that transport of IAA into and out of plant cells involves both intramembrane receptors (such as TIR1/AFBs) and extramembrane receptors (such as ABL and TMK) and that IAA does not directly enter plant cells from the outside, it will be necessary to further investigate the activity of both intramembrane and extramembrane receptors in order to characterize the mechanism by which *Pantoea* sp. MCC16 increases root IAA levels. In addition, although more IAA-producing bacteria were screened in the rhizosphere of normal potato plants, the IAA-producing ability of such bacteria in the rhizosphere of CC potatoes was stronger (Figure 3D). Moreover, similar to previous studies (Zhao et al., 2020), the IAA-producing bacteria screened in the rhizosphere of normal potato plants were all Firmicutes, whereas those in CC soil were all Proteobacteria (Figure 3E; Supplemental Figure 4C). This result may reflect the continuous accumulation of root exudates due to CC, which changed the soil environment and excluded those microorganisms for which the growth conditions were not suitable. Although many studies have reported the potential of *Pantoea* to promote plant growth (Hernández-Forte et al., 2022; Kumar et al., 2022), the genus is not common in CC systems, possibly because of differences in sampling times and CC duration.

### Importance of root exudates in self-repair of the plant–soil system

Changes in the composition of root exudates during plant growth lead to corresponding changes in the structure of the rhizosphere microbial community (Shi et al., 2015; Cordovez et al., 2021; Santoyo, 2022). However, when plants are exposed to external environmental stress, the composition and content of root exudates often differ significantly from those observed under normal conditions during the same period (Stringlis et al., 2018; Gong et al., 2023). There has been extensive research on root secretions during the formation of resilient soil. For example, after infection by *P. syringae* pv. *tomato* for five consecutive generations, *Arabidopsis thaliana* secretes increased amounts of long-chain organic acids and amino acids to regulate the soil bacterial community and increase plant resistance (Yuan et al., 2018). Wild soybean (*Glycine soja*) exudes key metabolites, such as purines, to recruit beneficial *Pseudomonas* strains against salt stress (Zheng et al., 2024). In this study, at 20 DAE of CC potatoes, roots increased the secretion of nobiletin to recruit *Pantoea* sp. MCC16 and promote the establishment of seedling morphology (Figures 4D and 5C). The secondary metabolite nobiletin is a member of the polymethoxyflavonoids, a subclass of flavonoids (Kawai et al., 2007). Flavonoids, phenylpropanoids, nitrogenous compounds, and terpenoids are the main categories of root-specific metabolites and play crucial roles in regulating the assembly of specific microbial populations in the rhizosphere (Singh et al., 2023; Wang et al., 2024b), mainly serving as signaling substances or growth substrates in the microbial recruitment process (He et al., 2022). In this study, through preliminary verification, we found that *Pantoea* sp. MCC16 could not use nobiletin as a substrate (Supplemental Figure 11). Therefore, nobiletin may effectively recruit *Pantoea* sp. MCC16 because the bacterium contains flavonoid-sensing regulators. Plant-derived flavonoids can be sensed by the TetR regulator PhlH in *P. fluorescens 2P24* and act as important signaling molecules, providing a sensing mechanism that facilitates the movement of microorganisms at the root–soil interface and increases their colonization on the root surface (Yu et al., 2020). However, the proliferation of *Pantoea* sp. MCC16 requires nutrients whose availability may be significantly influenced by certain substances in the soil. It is currently unclear whether these substances are produced by the host plant’s root exudates or result from changes in soil microbial community structure, potentially leading to an increase in microorganisms whose secreted metabolites support the growth of *Pantoea* sp. MCC16 or to a decrease in microorganisms that inhibit its growth.

The shikimic acid pathway is one of the main secondary metabolic pathways involved in plant responses to external stress, and IAA, nobiletin, and vanillin are all secondary metabolites produced through the shikimic acid pathway in potatoes. Both nobiletin and vanillin are produced by the phenylalanine metabolism pathway, whereas IAA is produced by the tryptophan metabolism pathway. Both pathways use chorismate as the initial precursor for synthesis (Maeda and Dudareva, 2012). In this study, the IAA content of CC potatoes decreased, suggesting that the contents of phenylalanine-pathway products may have increased. In addition, vanillin is synthesized from ferulic acid (Gallage and Møller, 2015), whereas nobiletin is a flavonoid. Synthesis of both ferulic acid and flavonoids relies on a common precursor, p-coumaric acid (Mandal et al., 2010). We found that the nobiletin content of CC potatoes increased significantly, suggesting that vanillin synthesis in these plants may have decreased, consistent with previous results (Ma et al., 2023b). This strategy not only promotes plant growth in the current season but also gradually reduces the amount of autotoxic substances released into the soil, thereby improving the soil environment in subsequent CC seasons. Because self-repair is initiated by promoting plant growth at the seedling stage under CC vanillin stress, this strategy also explains, at least in part, why CC potato plants choose to secrete nobiletin to recruit *Pantoea* sp. MCC16.

Although the findings of this study demonstrate that potato plants adapt to CC obstacles under vanillin stress in the soil, this does not preclude the possibility that soil vanillin content may decrease as the adaptive process progresses. A recent study showed that tobacco adapts to CC obstacles primarily by reducing soil vanillin content (Xuan et al., 2024), in contrast to the results of the present study. This discrepancy may be attributed to the shorter duration of CC in the current study or to variations in microbial community composition across different regions, which lead to distinct optimal strategies for different plants. Furthermore, the findings of this study indicate that *Pantoea* sp. MCC16 is a key functional strain that alleviates potato CC obstacles, nobiletin is the key root exudate that recruits *Pantoea* sp. MCC16, and 20 DAE is a critical period for adaptation to CC obstacles. However, factors such as region, climate, and cultivar may lead to differences in the primary functional strains that help potatoes adapt to CC obstacles, the main root exudates that recruit functional strains, and the critical timing of recruitment. Despite these differences, the underlying mechanism for alleviation of CC obstacles identified in this study remains broadly applicable.

Although self-repair capabilities have been demonstrated in long-term CC systems, this ability cannot be relied upon to compensate for the consequences of inappropriate agricultural practices. In practical production, annual yield losses can severely impact laborers’ income and industrial development. To protect and enhance these vital agricultural ecosystems, sustainable agricultural management strategies must be implemented. The direct application of beneficial bacteria to the soil is currently the primary method used in agriculture to regulate plant growth. However, owing to unsuitable soil environments and ecological niches, as well as competition for resources with indigenous microorganisms, beneficial bacteria often fail to effectively colonize the rhizosphere (Alabouvette et al., 2009; Wang et al., 2019). The use of beneficial bacteria from the indigenous microbial community may therefore represent an effective solution. In this study, application of the beneficial bacterium *Pantoea* sp. MCC16 to CC soil significantly increased potato yields by 51.09% compared with the control (Figure 3F). Moreover, in large-scale field production, the direct large-scale application of bacterial inoculants may consume substantial resources. The enrichment of target bacterial strains in the soil at the appropriate time may thus provide a more efficient approach. In this study, *Pantoea* sp. MCC16 only became enriched in the rhizosphere 20 days after the emergence of CC potatoes, which was clearly disadvantageous for early plant development. Therefore, nobiletin was applied to the soil on the day that the potatoes emerged. Potato root biomass and aboveground growth were significantly higher in nobiletin-treated plants than in the controls at 10 DAE, ultimately leading to a 35.86% increase in potato yield (Figure 5D and 5E; Supplemental Figure 9). Although the direct application of nobiletin was less effective than the direct application of *Pantoea* sp. MCC16, the findings confirmed the feasibility of this approach. Therefore, manipulating the timing and composition of root exudates can effectively enhance the colonization of beneficial bacteria and lead to more sustainable and efficient agricultural practices, reducing reliance on synthetic fertilizers and pesticides. By identifying and promoting the growth of indigenous beneficial bacteria, farmers can enhance plant health and resilience, leading to higher yields and reduced environmental impacts. The use of natural compounds such as nobiletin to stimulate beneficial bacterial growth could also represent a cost-effective and environmentally friendly strategy for improving crop performance.

In conclusion, this study revealed that potatoes mitigate CC obstacles by increasing root IAA content and AR numbers under vanillin stress in the soil. Potato roots counteract vanillin stress by secreting nobiletin, attracting the high-IAA producing bacterium *Pantoea* sp. MCC16, raising the levels of IAA in the rhizosphere, enhancing root IAA uptake, increasing AR numbers, and improving plant resilience, thereby alleviating CC challenges (Figure 6).

## Methods

### CC field experiment and sample collection

The potato CC positioning test was initiated in 2011 at the potato experimental station (35°6′41″ N, 103°59′4″ E, 2276 m above sea level) in Weiyuan County, Dingxi City, Gansu Province, China, with an annual average temperature of 6.8°C, an annual rainfall amount of 500 mm, and a frost-free period of 166 days. The soil type was black loam, which is loose, soft, and easy to cultivate. The physical and chemical properties of the soil in the cultivated layer (0–20 cm) of the abandoned land were as follows: total nitrogen 0.746 g kg^−1^, total phosphorus 0.638 g kg^−1^, alkaline nitrogen 42.02 mg kg^−1^, available phosphorus 13.80 mg kg^−1^, available potassium 184.99 mg kg^−1^, organic matter 16.26 g kg^−1^, and a pH of 8.46. The only variable in this test was the number of CC years. Each year, a new piece of land was introduced as a CC treatment for 1 year. By 2020, the plots had durations of CC ranging from 1 to 10 years. Owing to the short frost-free period in the area, potatoes are planted in early May and harvested in early October each year. The potato variety was Longshu no. 7 (pre-elite seed), which was provided by the Potato Research Institute of the Gansu Academy of Agricultural Sciences (Dingxi, China). Each CC period was represented by one plot, with an area of 48.6 m^2^ (9 × 5.4 m), row spacing of 60 cm, plant spacing of 30 cm, and planting density of 5.56 × 10^4^ plants ha^−1^. Fertilization occurred on the day of potato planting and included 150 kg ha^−1^ nitrogen fertilizer (urea, 46% N; one-quarter of the nitrogen fertilizer was top-dressed during the budding stage in combination with soil cultivation), 120 kg ha^−1^ phosphate fertilizer (diammonium phosphate, 18% N and 46% P_2_O_5_), 75 kg ha^−1^ potassium fertilizer (52% K_2_O), and 37.5 t ha^−1^ of organic fertilizer (fresh sheep manure). Weeding was performed manually, and 25% leucine was applied during the budding stage to control aphids. Metalaxyl manganese zinc (58%) was used to control late blight, with four sprays performed based on field conditions.

On the basis of previous research (Ma et al., 2023b), 7 years was selected as the representative period for potato CC obstacles. Therefore, the CC treatment in this study refers to 7 years of CC, whereas the NCC treatment refers to 1 year of CC. Previous studies (Ma et al., 2023b) also indicated that the autotoxicity effect of CC significantly inhibits AR numbers in potato seedlings during the mid-seedling stage. However, field experiment observations revealed no significant difference in AR numbers between CC and NCC during the late seedling stage. To further investigate the changes in AR number during the seedling stage, we collected CC and NCC potato plants from the field and analyzed plant morphology, root IAA content, root expression of genes related to IAA synthesis and response, soil vanillin content, and yield. Details of sample collection are provided in Supplementary Method 1.

### Soil sterilization and microbial transfer experiments to identify soil microbial functions in CC

#### CC soil sterilization test

Four treatments were used: unsterilized CC soil, CCS, unsterilized NCC soil, and sterilized NCC soil. The test soil and seed potatoes (Longshu no. 7, pre-elite seed) were transported from Weiyuan Potato Experimental Station, Dingxi City, Gansu Province, China, to Sichuan Agricultural University (Chengdu, China) in late November 2021. For the sterilization treatment, we used two high-pressure steam sterilization events (121°C, 1.5 h) with an interval of 3 days (Zheng et al., 2017). After the second sterilization, sterilized soil extract was isolated and cultured on Luria–Bertani (LB) solid medium to ensure that no bacteria could be isolated from the soil. The specific test procedure was as follows. First, 30 pots were set up for each treatment (300 mm in upper diameter × 210 mm in height). Each pot was filled with 4 kg of soil and then planted with one grain of whole potato weighing approximately 50 g at a sowing depth of 10 cm. The seed potatoes were disinfected with 0.1% sodium hypochlorite solution for 20 min and then washed with sterile distilled water. When potato plants had emerged to approximately 1 cm, they were watered with 200 ml Hoagland nutrient solution once every 7 days. The application times of the Hoagland nutrient solution were increased appropriately according to the potato growth, and watering ceased after the tuber-formation stage. Throughout the potato growth process, the water supply was appropriate, pests and diseases were controlled, and weeding was performed manually. For details of sample collection, please see Supplementary Method 2.

### Microbial flora transfer test

The rhizosphere soils of CC and NCC plants in the CC potato field test were used as the source of microbial flora, and potatoes were planted in the field as described in the first experiment. First, field cultivation was performed in early May 2022, and a new plot was used for the NCC treatment (with specific cultivation measures such as those in the first experiment). Before potato planting, 400 kg of CC topsoil was collected and transported to Sichuan Agricultural University for sterilization treatment (as described above), then used as the basic soil for the subsequent experiment. At 10, 20, and 30 DAE, 10 CC and 10 NCC plants were selected. Soil clods of approximately 20 cm around the stem base of the plants were loosened with a shovel. After the plants were gently pulled out, some of the soil was shaken off, but the roots remained wrapped in soil. The rhizosphere soil of different plants from the same treatment was then collected into the same aseptic bag with a sterile brush, mixed, and stored temporarily at 4°C.

To ensure maximum microbial activity in the rhizosphere soils, pot tests were performed on the day after rhizosphere soil collection (10, 20, and 30 DAE). Therefore, the experiment was divided into three periods with intervals of 10 days. The method for microflora transfer was based on Jin et al. (2023). The specific method was as follows. CC and NCC rhizosphere soils were transferred to sterile soil at a 3% (g) inoculation rate, with a total of 4 kg soil per pot (300-mm upper diameter × 210-mm height). Potatoes were disinfected with 0.1% sodium hypochlorite solution for 20 min before planting and then washed with sterile distilled water. One seed potato was planted in each pot at a planting depth of 10 cm. Fifteen pots were set up for each treatment, and the pots were placed in a light culture room for cultivation (12-h light, 12-h dark). When the potato plants had emerged to approximately 1 cm, 200 ml Hoagland nutrient solution was applied once every 7 days. The application times of the Hoagland nutrient solution were increased appropriately according to potato growth, and watering ceased after the tuber-formation stage. During the potato growth process, the water supply was appropriate, pests and diseases were controlled, and weeding was performed manually. Potato plant samples were collected from each treatment at 10, 20, and 30 DAE, and the effects of rhizosphere flora on potato AR number, root IAA content, rhizosphere soil IAA content, soil vanillin content, and yield were determined. Potato yield was measured in five pots.

### Profiling the potato rhizosphere microbiome

16S rRNA absolute quantitative analysis of rhizosphere bacterial communities was performed at 10 and 20 DAE in CC (CC10, CC20) and NCC (NCC10, NCC20) soils to explore the structure of the rhizosphere soil microbial community during the period. For details of 16S rRNA sequencing, please see Supplementary Method 3.

### Isolation of rhizosphere bacteria and screening of IAA-producing strains and their effects on potato growth

Rhizosphere bacteria were isolated and cultured at 10 (I), 20 (M), and 30 (F) DAE in the CC and NCC treatments. The specific steps were as follows. In June 2022, plants in CC and NCC soils were retrieved from the field, and rhizosphere soil was collected. Owing to the difference in sampling times, the standard continuous-dilution culture method was used to isolate culturable bacteria immediately after retrieval. Soil suspensions with different concentrations (*n* = 5) were plated on solid LB medium and cultured overnight at 37°C. After colony formation, labels were made successively according to colony morphology, and bacteria were picked out. Purified single colonies were obtained by plate marking on the corresponding LB medium, and the lines of single colonies were redrawn twice to ensure colony purity. Finally, the strains were inoculated into LB broth medium and stored at −80°C.

Salkowski’s reagent method (Sarwar and Kremer, 1995) was used to examine the IAA production capacity of all isolated bacteria. Seven strains of bacteria with an IAA production capacity greater than 20 μg ml^−1^ were identified, followed by sequence alignment (Supplementary Table 2; Supplemental Figure 4C). According to the identifications, the MCC16 strain and the unique strain ASV7733 identified in CC20 rhizosphere soil were the same strain, and this strain also had the strongest IAA production capacity. Therefore, an irrigation test with MCC16 bacterial solution was designed to determine whether this strain could promote the growth of CC potatoes. The specific design of this experiment is described in Supplementary Method 4.

### Pot experiment to determine the effect of CC root exudates on potato growth and *Pantoea* sp. MCC16

Root exudates of CC and NCC potato were collected at 10 and 20 DAE and used to irrigate CC potato. The specific procedure is described in Supplementary Method 5.

### Nontargeted metabolomics analysis and differential metabolite screening of root exudates

Nontargeted metabolomics analysis of root exudates from CC10, CC20, NCC10, and NCC20 was performed to identify which metabolites had major effects. Root exudates were collected, then freeze-dried in an LGJ-18S vacuum freeze dryer (Bonnin Instrument Technology, Wuhan, China) and stored at −80°C. The nontargeted metabolomics procedure is described in Supplementary Method 6. The purpose of the differential metabolite screening was to screen for substances that increased significantly in the root exudates of CC20 potato. Significance analysis (TOP50), random forest analysis (TOP40), KEGG pathway enrichment analysis (TOP10), and PCA (TOP40) were used to screen for substances that increased significantly in the root exudates of CC20 potato. The screened metabolic substances were significantly more abundant in CC20 than in CC10 and NCC20.

### Effects of differential metabolites on chemotaxis and biofilm formation by *Pantoea* sp. MCC16

The procedures used to detect chemotaxis and biofilm formation *in vivo* are described in Supplementary Method 7. Supplementary Method 8 describes the approach used to verify whether the target metabolite (nobiletin) increased AR numbers in CC potatoes through soil microorganisms. Chemotaxis and biofilm formation were then verified in the soil. First, sterilized CC soil was placed in pots (10 cm in upper diameter × 10 cm in height). Ten pots received injections of nobiletin, and 10 pots received injections of sterile water as a control. Each pot contained 400 g of soil, and 1 ml of *Pantoea* sp. MCC16 bacterial solution (1.0 × 10^8^ colony-forming units [CFU] ml^−1^) was injected vertically into the soil with a 1-ml syringe at four locations 4 cm from the center of the pot. A syringe was also used to vertically inject 10 ml of 20 μM nobiletin solution or sterile water as the control, and the pots were then placed at 37°C for constant temperature culture, during which the same amount of nobiletin solution was added every 3 days. The central soil column of the pots was collected with a hole punch (1-cm diameter) on the 10th day. Bacteria were isolated and cultured by the standard continuous-dilution culture method, and the number of *Pantoea* sp. MCC16 was counted.

### Effects of exogenous application of *Pantoea* sp. MCC16 and nobiletin on the growth of CC potato

Using CC soil as the background, the following four treatments were established: sterile water, nobiletin solution (20 μM), *Pantoea* sp. MCC16 bacterial solution (1.0 × 10^8^ CFU ml^−1^), and nobiletin solution combined with *Pantoea* sp. MCC16 bacterial solution. Twenty pots were set up for each treatment, and each pot (300-mm upper diameter × 210-mm height) was filled with 4 kg of CC soil and planted with one seed potato. In the nobiletin treatment, potatoes were watered at 3, 6, and 9 DAE. In the *Pantoea* sp. MCC16 bacterial solution treatment, pots were irrigated once with 350 ml on the day of potato emergence. At 10 and 20 DAE, potato plants were collected to determine plant morphology, AR numbers, root IAA content, rhizosphere soil IAA content, and soil vanillin content. The yield of 10 plants per treatment was determined at harvest.

### Statistical analyses

Community alpha diversity indices (i.e., operational taxonomic unit [OTU] number, Shannon index, and abundance-based coverage estimator [ACE] index) were calculated using the vegan package in R (Oksanen et al., 2019). Beta diversity was analyzed and visualized using Bray–Curtis dissimilarity-based principal coordinate analysis. To test the effects of CC on community dissimilarities (i.e., whether there were differences in beta diversity), a PERMANOVA test was performed using the Adonis function in the vegan 2.6-4 package (Helsinki University, Finland) for 9999 permutations (Oksanen et al., 2019). Functional inference was performed using PICRUSt2.0 against the KEGG pathway database (Douglas et al., 2020). The *p* values were corrected for multiple comparisons using the Benjamini–Hochberg false discovery rate method (Lydersen, 2024). To identify the exudate compounds that made the greatest contributions to the classification, the random forest method and default parameters of the algorithm R implementation were used (R package RandomForest, ntree = 1000). Correlations were examined using Pearson correlation analysis. Enrichment analysis of metabolic pathways was performed using the online platform MetaboAnalyst (http://www.metaboanalyst.ca/faces/home.xhtml). A phylogenetic tree of bacterial rhizosphere isolates was constructed using MEGA 6 (Temple University, Philadelphia, PA, USA). Phylogenetic trees were annotated and visualized using Adobe Illustrator software (Adobe, San Jose, CA, USA).

All data were tested for normality (Shapiro–Wilk test) and homogeneity of variance (Levene’s test). To analyze microbial abundance data, we applied logarithmic transformation. Comparisons between two groups were performed using Welch’s *t*-test, whereas comparisons among three or more groups were performed by one-way ANOVA followed by Tukey’s honestly significant difference (HSD) test. Statistical significance was determined at *p* < 0.05. Figure 3B was plotted using the Bioinformatics online platform (https://www.bioinformatics.com.cn/). Figures 4D and 5A were plotted using the Chiplot online platform (https://www.chiplot.online/). Figure 6 was plotted using the Figdraw online platform (https://www.figdraw.com/static/index.html#/). Supplemental Figure 1 was prepared in PowerPoint (Microsoft Corporation, Redmond, WA, USA). Other figures were plotted using GraphPad Prism 9 (GraphPad Software, La Jolla, CA, USA).

## Data and code availability

Raw 16S rRNA sequencing data were deposited in the Genome Sequence Archive at the China National Genomics Data Center under accession number CRA015542 (https://ngdc.cncb.ac.cn/gsa/), and metabolite profiling data were deposited in the EMBL-EBI metabolomics repository under the identifier MTBLS12358 (https://www.ebi.ac.uk/metabolights/search).

## Funding

This research was supported by the 10.13039/501100018542Natural Science Foundation of Sichuan Province (grant number 2022NSFSC0014), the Tackling Key Problems and Supporting Projects of Breeding in Sichuan Province (grant numbers 2021YFYZ0019 and 2021YFYZ0005), the Sichuan Potato Innovation Team (grant number sccxtd–2023–09), and the 10.13039/501100001809National Natural Science Foundation of China (grant number 32060443).

## Acknowledgments

We extend our gratitude to Juanjuan Liu, Wensen Huang, Yang Li, and Xinyu Zheng for their invaluable assistance. No conflict of interest is declared.

## Author contributions

H.M., Z.R., and A.L. conceived and designed the study. H.M., X.F., R.L., X. Shi, C.W., K.Z., and S.Z. performed the experiments and analyzed the data. H.M., X. Sun, J.L., and H.L. wrote the manuscript. All authors edited the manuscript and approved the final version.

## Supplemental information

Document S1. Supplemental Figures 1–12 and Supplemental Tables 1–3

Document S2. Article plus supplemental information
