# Supplementary material for: Self-alleviation of continuous-cropping obstacles in potato via root-exudate-driven recruitment of growth-promoting bacteria
Source: Plant Commun. 2025 May 12;6(7):101372. doi: 10.1016/j.xplc.2025.101372 (PMC12281297; doi:10.1016/j.xplc.2025.101372)
Supplement: Document S1. Supplemental Figures 1–12 and Supplemental Tables 1–3 [file mmc1.pdf]

Plant Communications, Volume *6*

Supplemental information

Self-alleviation of continuous-cropping obstacles in potato via root-exudate-driven recruitment of growth-promoting bacteria

Haiyan Ma, Zhitong Ren, Aihua Luo, Xiaoting Fang, Ruilin Liu, Chao Wu, Xinxin Shi, Junji Li, Heping Lv, Xiaohua Sun, Kaiqin Zhang, and Shunlin Zheng

Self-alleviation of continuous cropping obstacles in potato via root exudate-driven recruitment of growth-promoting bacteria

Haiyan Ma[1,2,5,6], Zhitong Ren[1,2,6], Aihua Luo[3,6], Xiaoting Fang[1,2], Ruilin Liu[1,2], Chao Wu[1,2], Xinxin Shi[1,2], Junji Li[1,2], Heping Lv[3], Xiaohua Sun[3], Kaiqin Zhang[1,2], and Shunlin Zheng[1,2,4*]

[1] State Key Laboratory of Crop Gene Exploration and Utilization in Southwest China, College of Agronomy, Sichuan Agricultural University, Chengdu 611130, P.R. China

[2] Crop Ecophysiology and Cultivation Key Laboratory of Sichuan Province, Chengdu 611130, China

[3] Potato Research Institute of Gansu Academy of Agricultural Sciences, Lanzhou, 730070, China

[4] Key Laboratory of Tuber Crop Genetics and Breeding, Ministry of Agriculture, Chengdu Joyson Agricultural Technology Co., Ltd, Xindu 610500, China

[5] Yibin Academy of Agricultural Sciences, Yibin, 644699, China

[6] Co-first author

* **Correspondence: Shunlin Zheng (zhengshunlin123@163.com).**

Supplemental information

Supplementary Methods

Supplementary Method 1. Sample collection from continuous cropping field

For plants where the morphological indices were measured, sample preperation began with repeated shaking to remove most of the soil, followed by the collection of rhizosphere soil. Three rhizosphere soil samples were combined to form a single composite sample for the determination of the vanillin content. After rhizosphere soil collection, the roots were rinsed thoroughly with deionized water, air-dried, and measurements such as the plant height, stem diameter, and number of adventitious roots were recorded. The plant organs were then separated and initially heat-killed for 30 min at 105°C before being dried at 80°C until reaching a constant weight to determine the dry matter content. For the assessment of root IAA content and related gene expression, plant roots were washed with deionized water post-low temperature treatment and a 2-cm segment of the root tip was excised, promptly immersed in liquid nitrogen for freezing for 10 min, and then stored at −80°C. The IAA content in the roots was quantified using a plant growth hormone (IAA) ELISA kit (Jiangsu Meimian Industrial Co., Ltd., China). Total RNA extraction from the roots was conducted using the Trizol method to ensure adequate concentration and purity, and the extracted RNA was then reverse transcribed into cDNA using the HiScript III 1st Strand cDNA Synthesis Kit (Nanjing Nuoweizan Biotechnology Co., LTD., China). Gene expression analysis was conducted via qPCR using AceQ qPCR SYBR Green Master Mix (Nanjing Nuoweizan Biotechnology Co., LTD., China) on a QuantStudio 6 Flex instrument (Thermo Fisher, USA), utilizing the QuantStudio™ Real-Time PCR System software (Thermo Fisher, USA). The expression levels of target genes were calculated according to the $2^{-\Delta\Delta Ct}$ method, with StEF-1α serving as the internal reference gene. The primer sequences for these genes are listed in Supplementary Table 1. After harvesting, 20 potato plants from each treatment group were selected to assess the yield, which was then scaled up to calculate the yield per hectare. On the day of potato planting, a five-point sampling strategy was employed to clear away the soil surface stubble. Topsoil samples from both the continuous cropping (CC) and noncontinuous cropping (NCC) treatments were collected immediately following potato emergence and considered the day 0 soil samples. Subsequently, the soil vanillin content was evaluated in conjunction with rhizosphere soil samples collected at 10, 20, and 30 days after emergence (DAE). The extraction of vanillin from the soil was performed following the method outlined by Li et al. (2010). Phenolic acid quantification was conducted using a Shimadzu LC-20A liquid chromatograph (Shimadzu Corporation, Kyoto, Japan) equipped with an Agilent C18 column (dimensions: 4.6 mm × 250 mm, particle size: 5 μm). The analysis involved a flow rate of 1 mL min^{-1}, a column temperature of 25°C, and a UV detection wavelength of 280 nm, as described by Bai et

al. (2019).

Supplementary Method 2. Sample collection from the soil sterilization pot experiment

Plant and soil samples were systematically collected at 10, 20, and 30 days after emergence (DAE) to quantify various parameters, including the number of adventitious roots (ARs), the root IAA content, the expression of root-related IAA genes, the IAA content in the rhizosphere soil, the vanillin content in the rhizosphere soil, and the potato yield. At each sampling point, six plants were randomly selected for the assessments, with half (three plants) used for the measurement of physiological indices and the remaining three plants utilized to count the number of ARs. The procedure employed to collect potted plant samples was as follows: the selected plants were carefully removed from their pots and the bulk soil was gently shaken off. The rhizosphere soil was then meticulously collected using a sterile brush and deposited into a sterile sampling bag for the determination of the IAA and vanillin concentrations in the rhizosphere soil. These samples were temporarily stored at 4°C. Soil samples obtained from plants under the same treatment conditions were homogenized. After soil sample collection, the roots were thoroughly washed with deionized water and air-dried. Next, a 2-cm section of the root tip was excised from each individual root and placed in a cryovial, rapidly frozen in liquid nitrogen, and subsequently transferred to a −80°C freezer for storage, awaiting assays for root IAA content using an IAA ELISA kit (Jiangsu Meimian Industrial Co., Ltd., China) and gene expression analyses. Finally, the number of ARs was obtained, and 10 pots from each treatment were selected to determine the potato yield.

Supplementary Method 3. 16S rRNA sequencing of potato rhizosphere microbiome

Total genomic DNA was extracted from the soil samples using the FastDNA® SPIN Kit (MP Biomedicals, Santa Ana, CA, USA) according to the manufacturer's instructions. The quality, concentration, and purity of the extracted genomic DNA were validated using agarose gel electrophoresis, and measurements were conducted using both the Nanodrop 2000 and Qubit3.0 spectrophotometers. An internal standard mix containing gene fragments of known copy numbers (10^3, 10^4, 10^5, and 10^6 copies) was introduced into the sample DNA pools. These internal standard sequences contained conserved regions similar to those found in naturally occurring 16S rRNA genes and artificial variable regions, as previously described in Tkacz et al. (2018) and Jiang et al. (2019). To amplify the targeted V4–V5 hypervariable region of the 16S rRNA gene, the primers 515F (sequence: 5'-GTGCCAGCMGCCGCGG-3') and 907R (sequence: 5'-CCGTCAATTCMTTTRAGTTT-3') were utilized. The amplified products subsequently underwent sequencing using the Illumina NovaSeq 6000 platform (performed by

Genesky Biotechnologies Inc., located in Shanghai, China, zip code: 201315). To ensure that high-quality sequencing data were obtained, the DADA2 plugin within the QIIME2 software suite was used to conduct rigorous quality control measures, including filtering, denoising, merging, and chimera removal. This process generated feature tables and representative sequences (as documented by Callahan et al., 2016; Bolyen et al., 2019). Taxonomic assignments of ASV representative sequences were performed using a pre-trained Naive Bayes classifier trained on the RDP Ribosomal Database Project (RDP) database version 11.5. During the data processing, the internal standard sequences were recognized, and their read counts were tabulated; these sequences were later excluded from further analyses. Ultimately, each sample produced a standard curve based on its internal standard sequence reads, allowing sequences to be attributed to their corresponding samples based on each sample's unique barcode and thus enabling absolute quantification.

Supplementary Method 4. Effects of *Pantoea* sp. MCC16 bacterial solution watering test on potato growth

In this experiment, sterilized continuous cropping soil served as the base substrate and a total of 30 pots were prepared. Among them, 15 pots were inoculated with *Pantoea* sp. MCC16 bacterial solution, while the other 15 pots were treated with sterile water as controls. Each pot had dimensions of an upper diameter of 300 mm and a height of 210 mm and contained 4 kg of soil. Before planting, the potatoes were first disinfected in a 0.1% sodium hypochlorite solution for 20 min and then rinsed thoroughly with sterile distilled water. A single seed potato was planted in each pot at a planting depth of 10 cm. On the day of potato emergence, each pot received irrigation with either 350 mL of OD_{600}=1 bacterial solution (equivalent to 1.0×10^8 CFU mL^{-1}) or the same volume of sterile water to ensure that the final soil concentration of the bacterial solution was maintained at 1.0×10^8 CFU mL^{-1} per gram of dry soil in the inoculated pots. Plant samples were collected at three distinct time points at 10, 20, and 30 DAE. At these stages, several parameters were measured, including the number of adventitious roots, the root IAA content, the rhizosphere soil IAA content, the soil vanillin content, and the potato yield.

Supplementary Method 5. Effects of root exudates on potato plant growth

At 10 and 20 DAE, three plants from each continuous cropping and noncontinuous cropping treatment were carefully excavated to minimize root damage, ensuring that the roots remained enveloped in soil. These plants were swiftly transported back to the laboratory at a low temperature. Most of the adhering soil was delicately shaken off, and the roots were then rinsed with sterile distilled water. After rinsing, roots from plants

undergoing identical treatments were placed in a common beaker wrapped in tin foil to prevent light exposure. The beaker was then filled with sterile ultra-pure water containing 0.5 mM calcium chloride, and the roots were allowed to soak in this solution while being continuously aerated under dark conditions for a 12-h incubation period. Following incubation, the collected filtrate was filtered through a 0.22-μm filter membrane (Merck, Darmstadt, Germany). The plant roots were dried, and their fresh weight was recorded. The solution concentration was adjusted according to the fresh weight of the roots to standardize the results. The filtrate was then stored at −80°C following the methodology described by Williams et al.(2021). For the separate experiment involving the application of root exudates to potato plants, 10 pots were prepared for each root secretion filtrate treatment. Each pot was filled with 5 kg of natural soil collected from the CC environment and sowed with a single seed potato. On the day of potato emergence, 100 mL of root exudates was applied, followed by regular irrigation with 20 mL root exudates every 2 days until the 10th day post-emergence, following the methodologies of Valentinuzzi et al. (2015) and Zhou et al. (2023). The number of adventitious roots (ARs) was counted at 10 and 20 DAE, and the rhizosphere soil was collected to determine the population density of *Pantoea* sp. MCC16.

Supplementary Method 6. Nontargeted metabolomics analysis procedure

First, 100 mg of freeze-dried root exudates was dissolved in 500 μL of 80% methanol solution (Thermo Fisher, USA), vortexed for 30 s, placed on ice for 5 min, and then centrifuged at 15000 g and 4°C for 20 min (D3024R, Scilogex, USA). Following centrifugation,100 μL of the supernatant was collected, the methanol content in the solution was diluted to 53% using mass spectrometry-grade water (Merck, Germany), and the solution was centrifuged at 15,000 g and 4°C for 20 min, after which the supernatant was collected for analysis. Liquid chromatography–mass spectrometry (LC-MS) analysis was performed (Want et al., 2013) according to the following chromatographic conditions: the chromatograph used was the Vanquish UHPLC (Thermo Fisher, Germany), the chromatographic column was a Hypesil Gold column (100×2.1 mm, 1.9 μm) (Thermo Fisher, USA), the column temperature was set to 40°C, and the flow rate was set to 0.2 mL min^{-1}. Positive ion mode: mobile phase A: 0.1% formic acid, mobile phase B: methanol. Negative ion mode: mobile phase A: 5 mM ammonium acetate at pH 9.0, mobile phase B: methanol. The chromatographic gradient elution program was as follows: 0 min: A: 98%, B: 2%; 1.5 min: A: 98%, B: 2%; 12 min: B: 100%; 14 min: B: 100%; 14.1 min: A: 98%, B: 2%; 17 min: A: 98%, B: 2%. The mass spectrometry conditions were as follows: mass spectrometer: Q Exactive™ HF-X (Thermo Fisher, Germany), with a selected scanning range of m/z 100–1500. The settings for the ESI source were as follows: spray voltage 3.2 kV; sheath gas flow rate 40

arb; auxiliary gas flow rate 10 arb; capillary temperature 320°C; funnel RF level 40; auxiliary gas heater temperature 350°C; polarity positive and negative; and the MS/MS secondary scans were data-dependent scans. After the measurement was completed, CD 3.1 library search software was employed to screen each metabolite in the original offline data to determine the retention time, mass-to-charge ratio, and other parameters, and a retention time deviation of 0.2 min and a mass deviation of 5 ppm were set to compare different samples. Subsequently, the peaks were extracted and the peak areas were quantified, and then the target ions were integrated to predict the molecular formula based on the molecular ion peaks and fragment ions. Each metabolite was compared with the mzCloud (https://www.mzcloud.org/), mzVault and Masslist databases. After using blank samples to remove background ions, the original quantitative results were standardized, and finally the metabolites identification and relative quantification results were obtained.

Supplementary Method 7. In-vitro chemotaxis and biofilm formation assays

To explore which metabolites screened in CC20 stimulated the growth of MCC16, *in-vitro* chemotaxis and biofilm formation experiments were performed. The chemotaxis experiment was slightly modified according to a previously reported method (Rudrappa et al., 2008). Briefly, the MCC16 seed solution was first activated, diluted to a ratio of 1%, and then inoculated into fresh LB broth. Incubation was performed at a constant shaking speed of 170 r min^{-1} until OD_{600}=1, followed by centrifugation for 5 min (4°C, 5000 r min^{-1}).The solution was resuspended in an equal volume of chemotaxis buffer (100 mM K_3PO_4 (pH 7.0) and 20 μM EDTA) (Xiong et al., 2020), and a pipette was used to collect 100 μL of MCC16 bacterial suspension for later use. A 1-mL sterile syringe was employed to accurately draw 100 μL of 11 metabolites (N-isovalerylglycine, epimedin C, D-turanose, nobiletin, sauchinone, praeruptorin A, isomucronulatol-7-O-glucoside, methyl 4-hydroxy-3-methoxycinnamate, 3-succinoylpyridine, thioctic acid and kaempferol) at different concentrations (0, 10, 20, 40, 60, 80, and 100 μg mL^{-1}), after which the pipette containing MCC16 and the syringe containing metabolites were placed horizontally on a clean bench within a laminar flow hood with the pipette tip on the right and the syringe needle left. The needle was inserted into the pipette tip for 2 h. After 2 h, the syringe was carefully removed, the solution was spread over LB solid medium, and the plate dilutions were counted. Each treatment was repeated three times using chemotaxis buffer as a control. The number of colonies (CFUs) was counted and calculating the chemotaxis index (RCI) was calculated, which is the ratio of the number of treated colonies to the number of control colonies (when RCI>2, it is considered to have a significant difference).

The effects of metabolites on the biofilm-forming properties of MCC16 were determined

based on a previously described experimental method (Tan et al., 2013) with slight modifications. Briefly, the MCC16 seed liquid was first activated, then diluted to a ratio of 1% and inoculated into fresh LB broth. The solution was cultivated at a constant temperature of 37°C and 170 r min^{-1} until OD_{600}=0.2, then diluted 100 times, and we added 10 μL of metabolites per milliliter of bacterial suspension to transfer different concentrations (0, 10, 20, 40, 60, 80, and 100 μmol L^{-1}) of nobiletin into the bacterial suspension. The solutions were mixed thoroughly and transferred to a 96-well cell culture plate with 200 μL per well. Each treatment was repeated for eight times and incubated statically at 37°C for 24 h. The bacterial solution without the test substance was used as a control. After incubation, we carefully removed the culture medium under the biofilm was carefully removed from each well and the biofilm was gently rinsed three times with 250 μL of sterile physiological saline. After drying at room temperature, 200 μL of 0.1% crystal violet solution was added and let to stand for 30 min. The dye solution was discarded and then carefully rinsed four times with 250 μL of sterile saline and dried at room temperature. Following drying, 200 μL of 95% ethanol was added for decolorization, mixed, and diluted 40 times, and the absorbance was measured at a wavelength of 562 nm using a microplate reader (Bio-Rad, Microplate Reader 550, Hercules, CA, USA).

Supplementary Method 8. Effects of exogenous apply nobiletin on adventitious root (AR) numbers of potato seedlings in sterlized continuous cropping soil

First, starting with CC soil as the background, the soil was divided into two portions. One portion underwent sterilization treatment, while the other was kept in its natural, unsterilized state. Next, an exogenous nobiletin solution was applied to both the sterilized and unsterilized soils, with equal amount of sterile water serving as the control. Ten pots were set up for each treatment, and each pot (300 mm upper diameter × 210 mm height) was loaded with 4 kg soil and planted with 1 seed potato, and potato seedlings were irrigated with 100 mL of nobiletin solution (20 μM) at 3, 6, and 9 DAE. At 10 and 20 DAE, potato plants were collected to determine AR numbers.

Supplementary Figures

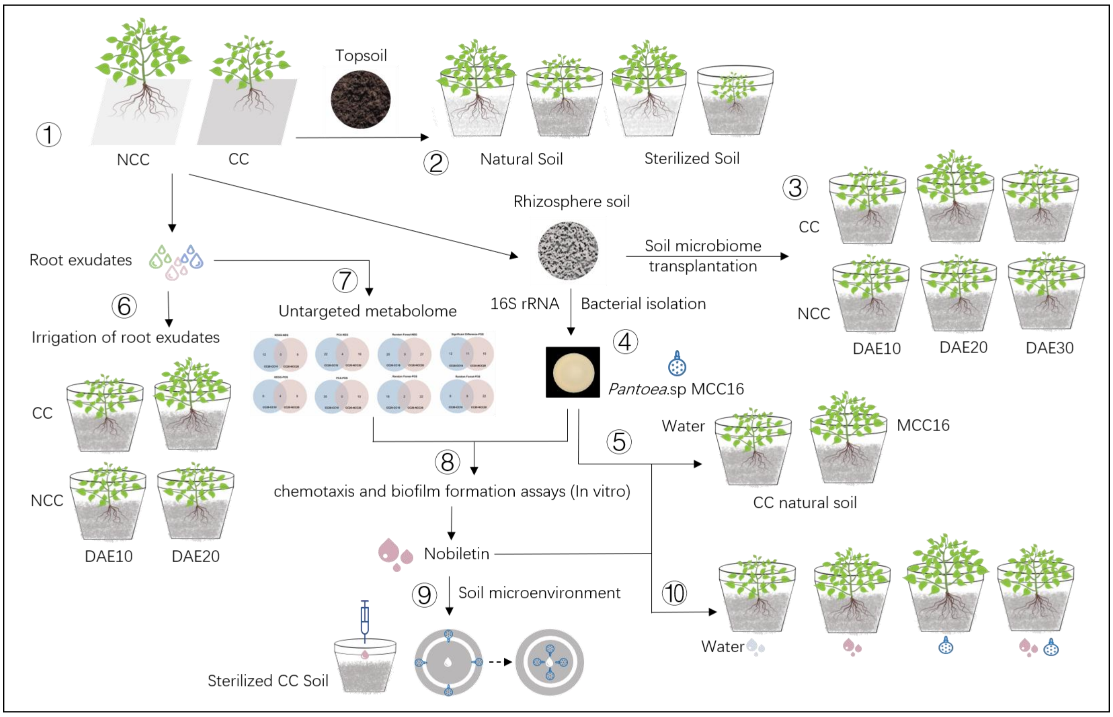

Supplementary Figure 1. Schematic representation of the experimental design.

First, compared with noncontinuous cropping (NCC), the inhibitory effect of continuous cropping (CC) on potato growth was verified ①. Then, field topsoil was collected and sterilized to determine whether soil microorganisms under CC promoted plant growth ② . Rhizosphere soil of potato seedlings was collected in the field for bacterial transplantation to observe when (DAE, days after potato emergence) soil microorganisms under CC promoted plant growth ③ . Subsequently, the target strain *Pantoea* sp. MCC16 was isolated by 16S rRNA absolute quantitative sequencing and pure culture ④, and then, the strain was applied to soil to verify its promotion of growth of CC potatoes ⑤. To determine which substances enriched *Pantoea* sp. MCC16 in CC soil, first, root exudates of CC potato seedlings were collected and applied to CC soil to observe the effects on potato plant growth ⑥. Subsequently, metabolites that increased significantly in root exudates of CC potato were screened by nontargeted metabolomics ⑦ and then analyzed with *Pantoea* sp. MCC16 for chemotaxis and biofilm formation ⑧. The metabolite nobiletin, which may recruit *Pantoea* sp. MCC16, was selected. Functional verification of nobiletin to recruit *Pantoea* sp. MCC16 was conducted in a microenvironment experiment ⑨ . Last, a pot experiment was used to confirm that exogenous nobiletin and *Pantoea* sp. MCC16 could effectively alleviate the CC barrier of potato ⑩.

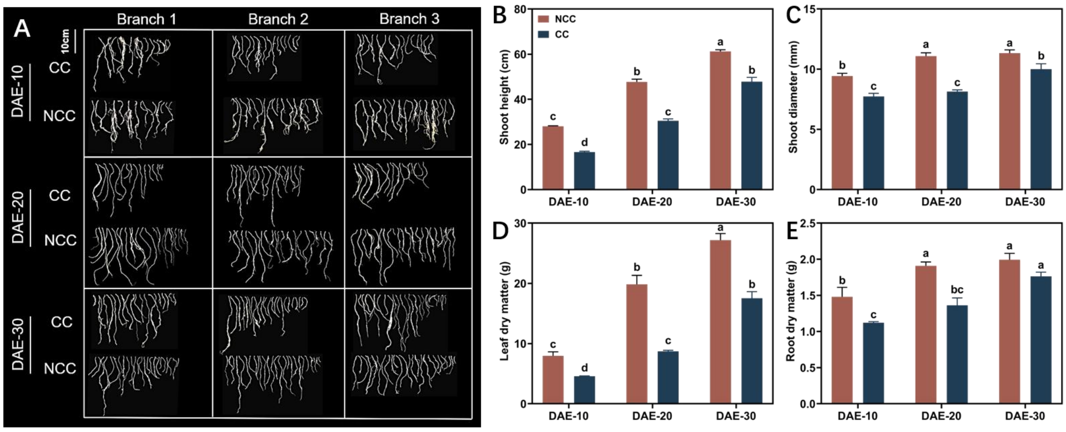

Supplementary Figure 2. Effects of continuous cropping (CC) and noncontinuous cropping (NCC) on potato seedling growth at 10, 20, and 30 days after emergence (DAE).

(A) Effect of continuous cropping (CC) and noncontinous cropping (NCC) on the number of adventitious roots (scale bar = 10 cm). Each plant contains three branches.

(B) Effects of CC and NCC on potato plant height.

(C) Effects of CC and NCC on potato shoot diameter.

(D) Effects of CC and NCC on potato leaf dry matter.

(E) Effects of CC and NCC on potato root dry matter.

Data are shown as the mean ± SEM (n = 3). Different letters indicate significant differences (Tukey's HSD test, $p < 0.05$).

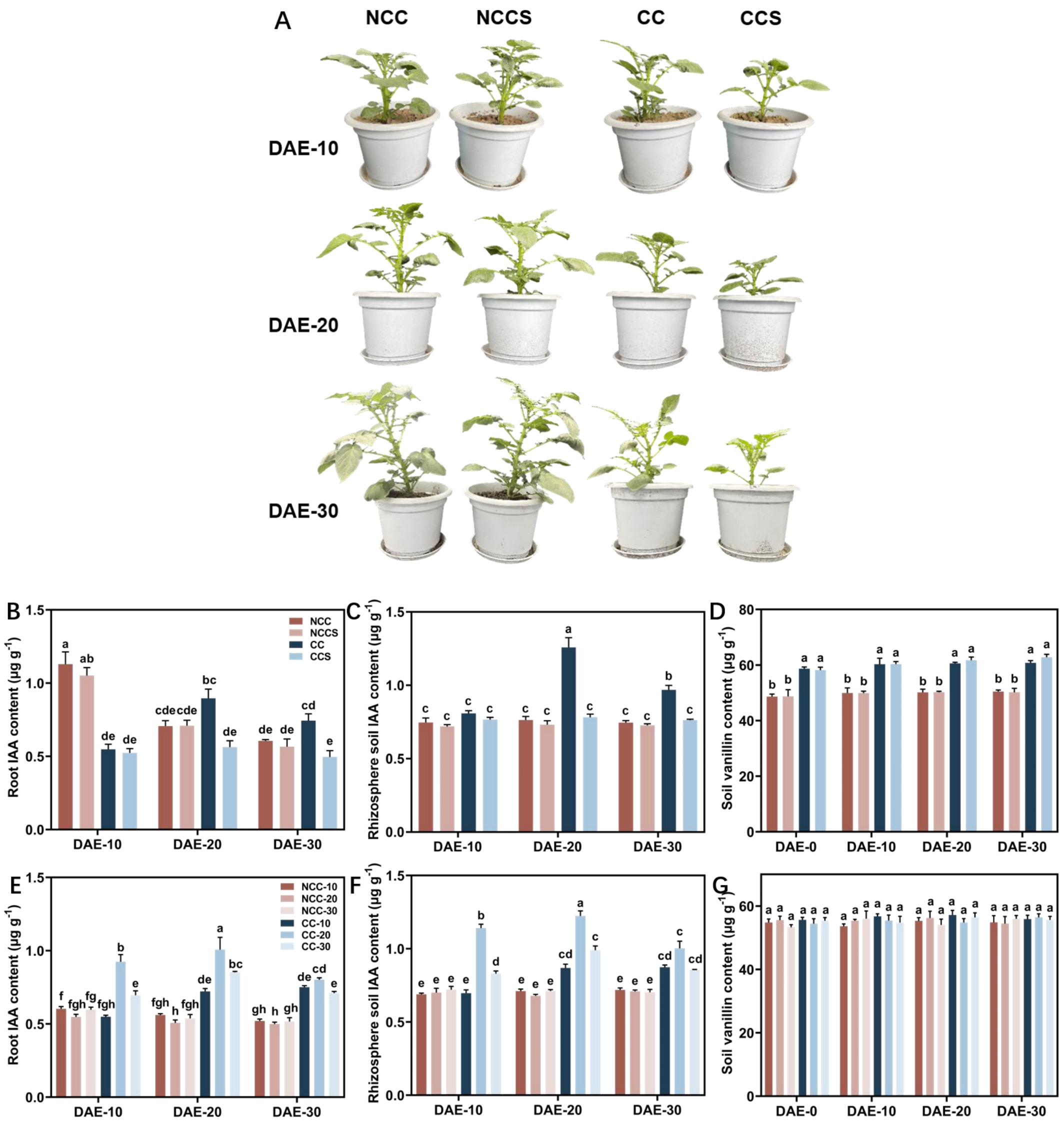

Supplementary Figure 3. Effects of soil sterilization and transplantation of continuous cropping soil microorganisms on potato plant growth.

(A) Effect of soil sterilization on potato plant growth.

(B) Effect of soil sterilization on root IAA content.

(C) Effect of soil sterilization on rhizosphere soil IAA content.

(D) Effect of soil sterilization on soil vanillin content.

(E) Effect of soil microflora transplantation on root IAA content.

(F) Effect of soil microflora transplantation on rhizosphere soil IAA content.

(G) Effect of soil microflora transplantation on soil vanillin content.

NCC: noncontinuous cropping natural soil; NCCS: noncontinuous cropping sterilized soil; CC: continuous cropping natural soil; CCS: continuous cropping sterilized soil. NCC-X: rhizosphere microorganisms on day X after potato emergence in noncontinuous cropping soils; CC-X: rhizosphere microorganisms on day X after potato emergence in continuous cropping soils; DAE-X: X days after potato emergence. IAA: indole-3-acetic acid. Data are shown as the mean ± SEM ($n = 3$). Different letters indicate significant differences (Tukey's HSD test, $p < 0.05$).

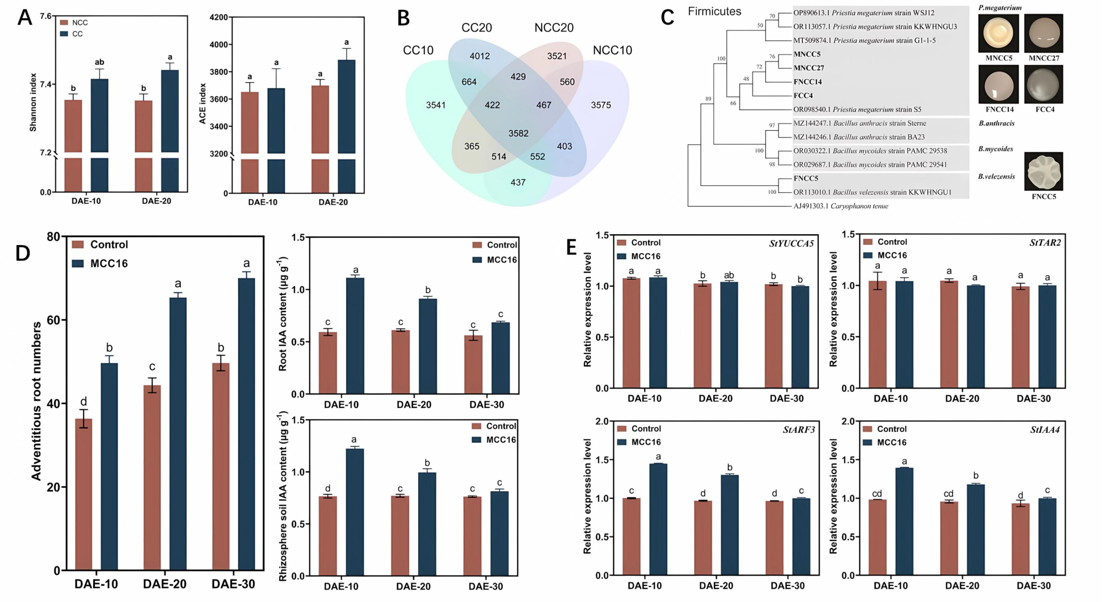

Supplementary Figure 4. Effects of continuous cropping on soil microbial community structure and irrigation with exogenous *Pantoea* sp. MCC16 on the growth of continuous cropping potato plants.

(A) α diversity analysis of the rhizosphere bacterial community structure in CC and NCC potato.

(B) Venn diagram of CC and NCC potato rhizosphere bacterial community diversity. The codes represent the number of amplicon sequence variants (ASVs) detected.

(C) Evolutionary tree analysis of five strains belonging to *Firmicutes*.

(D) Effects of exogenous *Pantoea* sp. MCC16 on the CC potato adventitious root (AR) numbers, root IAA content, and rhizosphere soil IAA content.

(E) Effects of exogenous *Pantoea* sp. MCC16 on the expression of IAA synthesis genes (*StYUCCA5 and StTAR2*) and response genes (*StARF3 and StIAA4*) expression in CC potato roots.

CC: continuous cropping, NCC: noncontinuous cropping. IAA: indole-3-acetic acid. DAE-X: X days after potato emergence. Data are shown as the mean ± SEM (n = 3). Different letters indicate significant differences (Tukey's HSD test, $p < 0.05$).

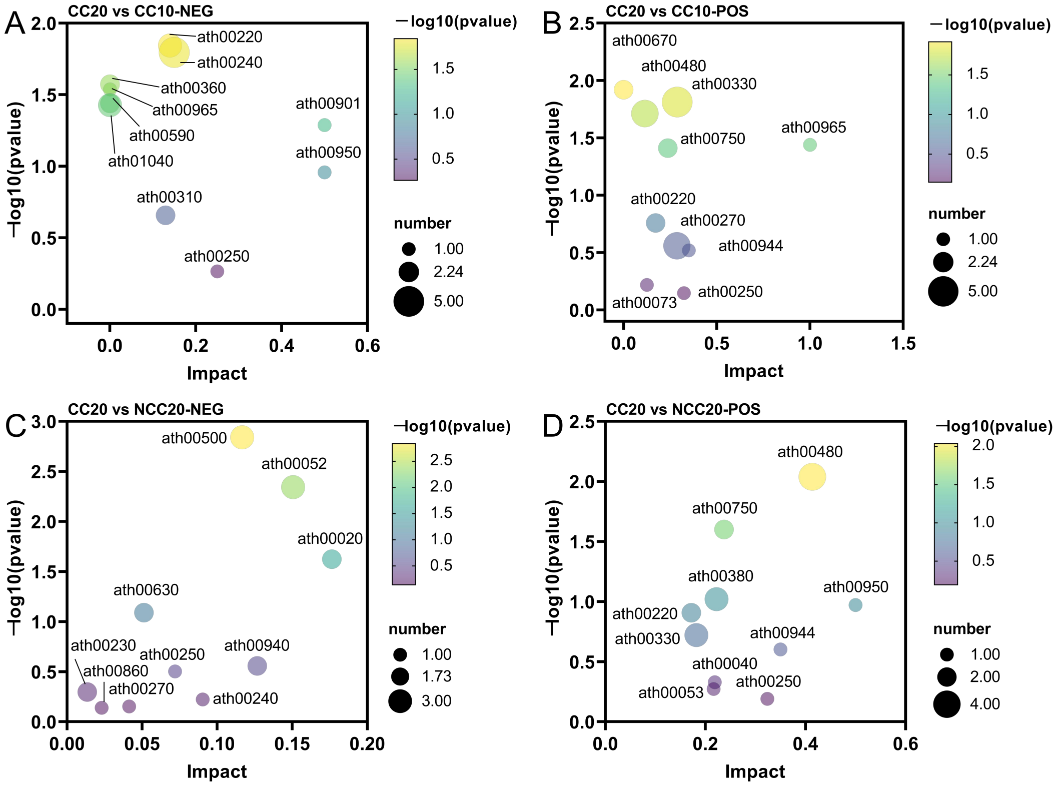

Supplementary Figure 5. Top 10 significantly enriched KEGG (Kyoto Encyclopedia of Genes and Genomes) pathways of differential root metabolites in CC20.

(A) Compared with CC10, the top 10 metabolic pathways demonstrating the significant enrichment of differential metabolites under negative ion mode were detected in the root exudates of CC20.

(B) Compared with CC10, the top 10 metabolic pathways demonstrating the significant enrichment of differential metabolites under positive ion mode were identified in the root exudates of CC20.

(C) Compared with NCC20, the top 10 metabolic pathways demonstrating the significant enrichment of differential metabolites under negative ion mode were detected in the root exudates of CC20.

(D) Compared with NCC20, the top 10 metabolic pathways demonstrating the significant enrichment of differential metabolites under positive ion mode were identified in the root exudates of CC20.

NCC-X: root metabolites on day X after potato emergence in noncontinuous cropping soils; CC-X: root metabolites on day X after potato emergence in continuous cropping soils. NEG: negative ion mode. POS: positive ion mode. Here, “number” represents the amount of significantly different metabolites enriched in this metabolic pathway, and “Impact” represents the contribution degree of difference formation. The more yellow the color, the more significant the difference.

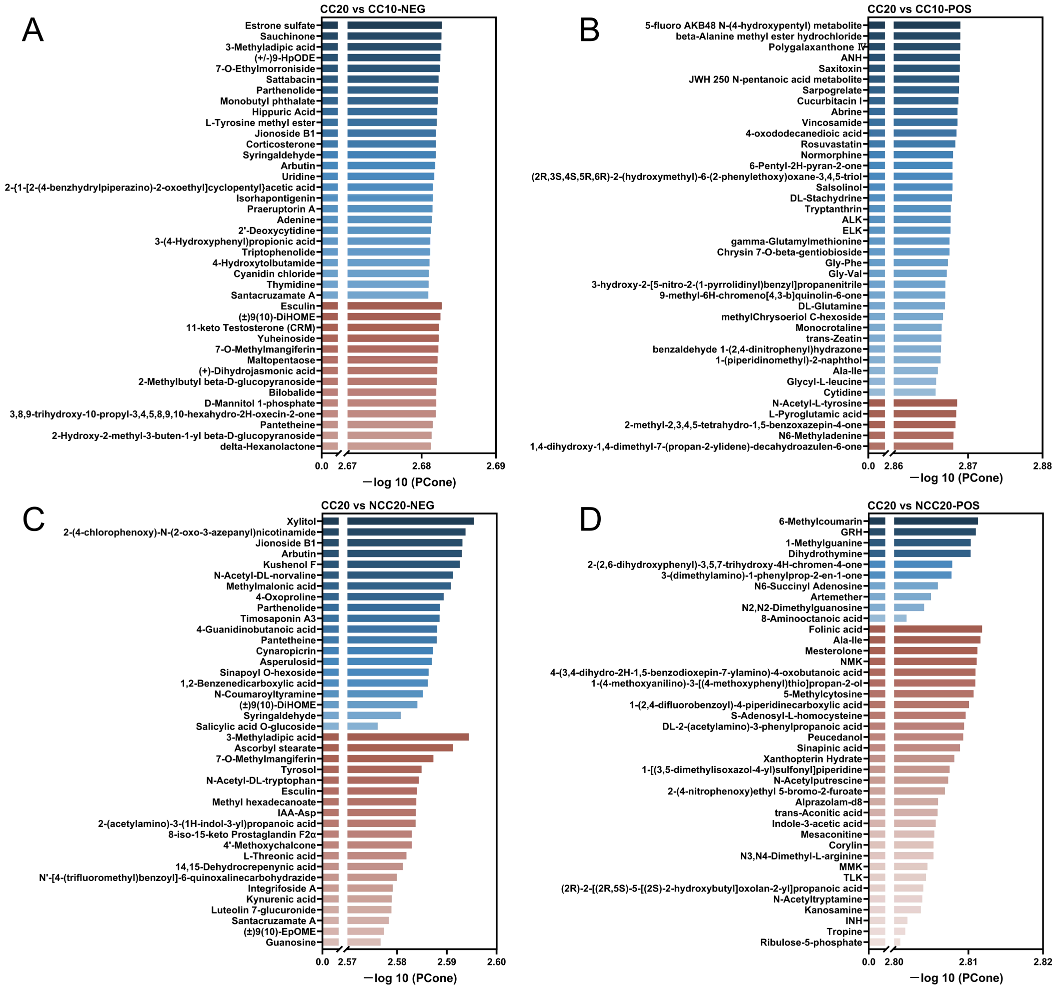

Supplementary Figure 6. Principal component analysis variable importance analysis (TOP40) of differential root metabolites.

(A) Ranking of significantly differential metabolites under negative ion mode in root exudates between CC10 and CC20.

(B) Ranking of significantly differential metabolites under positive ion mode in root exudates between CC10 and CC20.

(C) Ranking of significantly differential metabolites under negative ion mode in root exudates between NCC20 and CC20.

(D) Ranking of significantly differential metabolites under positive ion mode in root exudates between NCC20 and CC20.

NCC-X: root metabolites on day X after potato emergence in noncontinuous cropping soils; CC-X: root metabolites on day X after potato emergence in continuous cropping soils. Blue indicates enrichment in CC20 and red indicates enrichment in CC10 or NCC20, where the darker the color, the greater the importance.

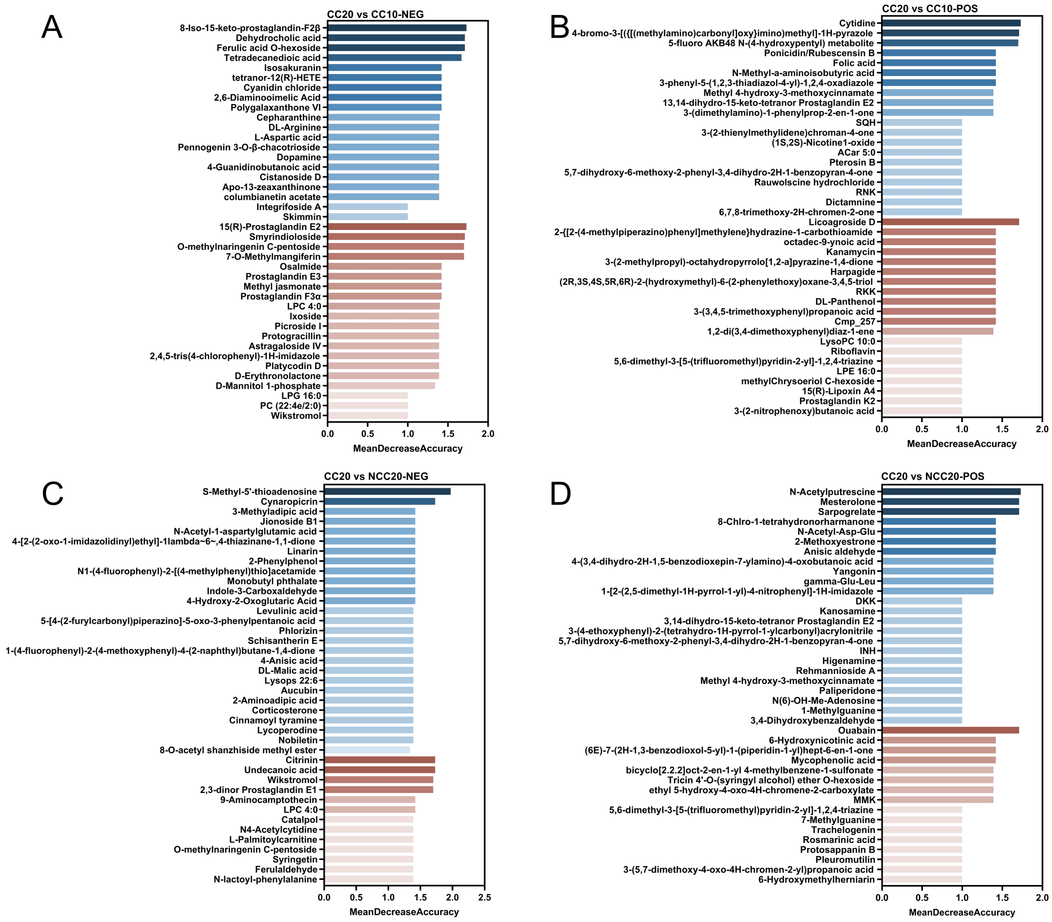

Supplementary Figure 7. Random forest variable importance analysis (TOP40) of differential root metabolites.

(A) Ranking of significantly differential metabolites under negative ion mode in root exudates between CC10 and CC20.

(B) Ranking of significantly differential metabolites under positive ion mode in root exudates between CC10 and CC20.

(C) Ranking of significantly differential metabolites under negative ion mode in root exudates between NCC20 and CC20.

(D) Ranking of significantly differential metabolites under positive ion mode in root exudates between NCC20 and CC20.

NCC-X: root metabolites on day X after potato emergence in noncontinuous cropping soils; CC-X: root metabolites on day X after potato emergence in continuous cropping soils. Blue indicates enrichment in CC20 and red indicates enrichment in CC10 or NCC20, where the darker the color, the greater the importance.

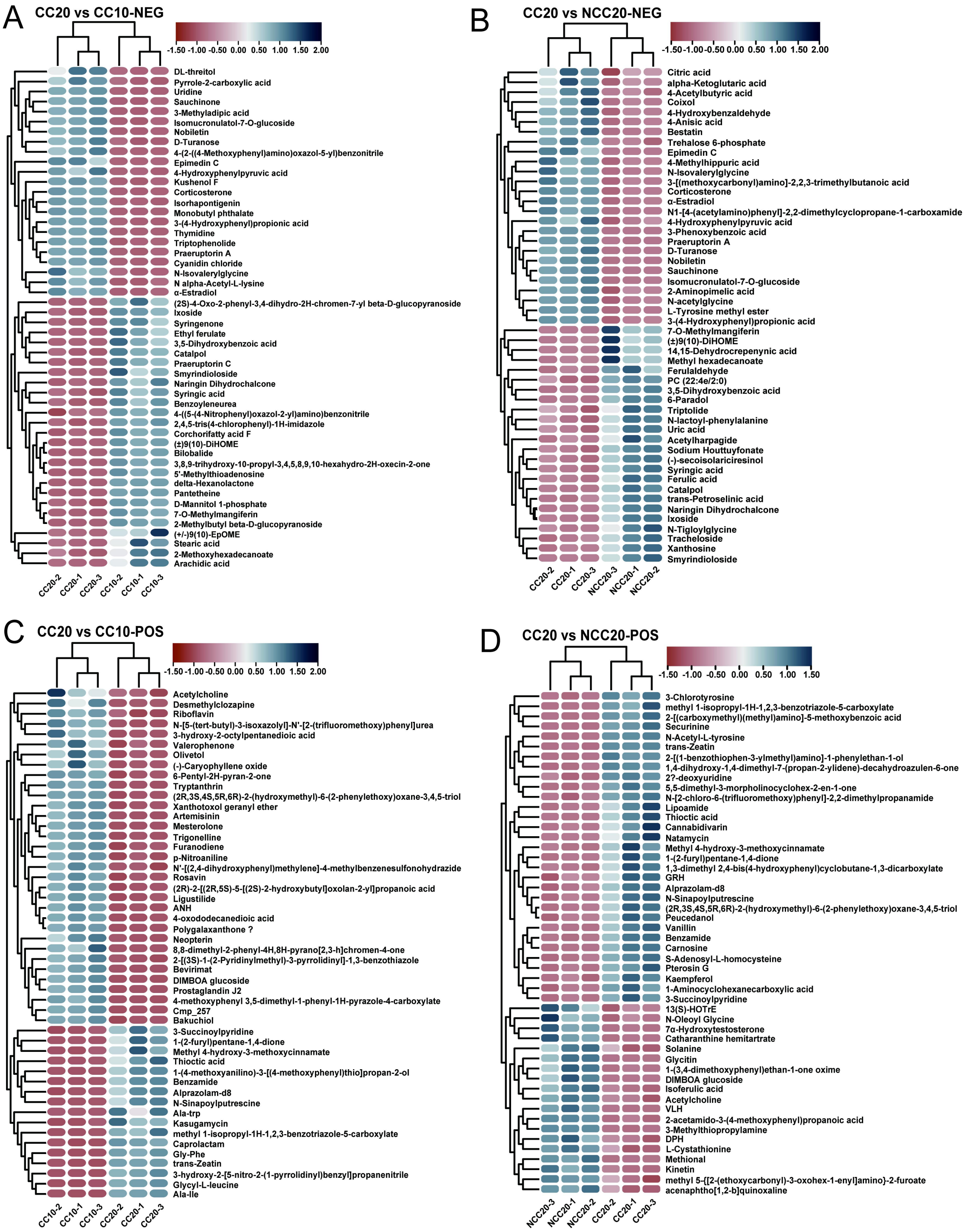

Supplementary Figure 8. Significance difference analysis (TOP50) of differential root metabolites.

NCC-X: root metabolites on day X after potato emergence in noncontinuous cropping soils; CC-X: root metabolites on day X after potato emergence in continuous cropping soils.

(A) Ranking of significantly differential metabolites under negative ion mode in root exudates between CC10 and CC20.

(B) Ranking of significantly differential metabolites under negative ion mode in root exudates between NCC20 and CC20.

(C) Ranking of significantly differential metabolites under positive ion mode in root exudates between CC10 and CC20.

(D) Ranking of significantly differential metabolites under positive ion mode in root exudates between NCC20 and CC20.

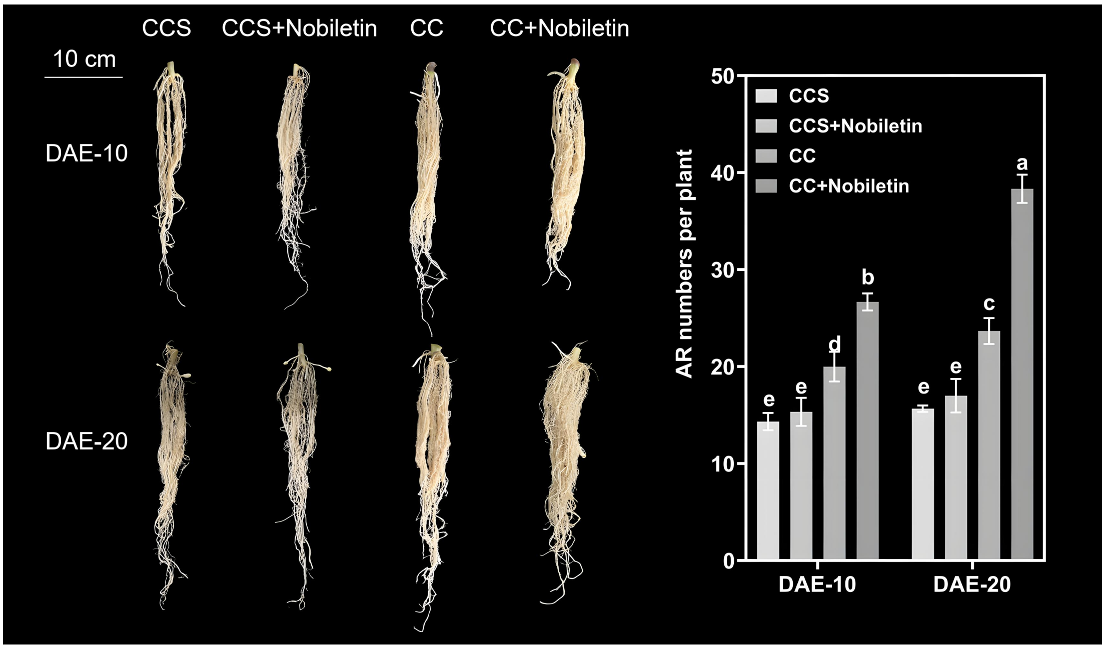

Supplementary Figure 9. Nobiletin enhances adventitious root (AR) numbers of potato seedlings in continuous cropping systems via soil microorganisms

CC:continuous cropping soil; CCS:sterilized:continuous cropping soil; CC+Nobiletin: apply nobiletin to CC soil; CCS+Nobiletin: apply nobiletin to CCS soil.

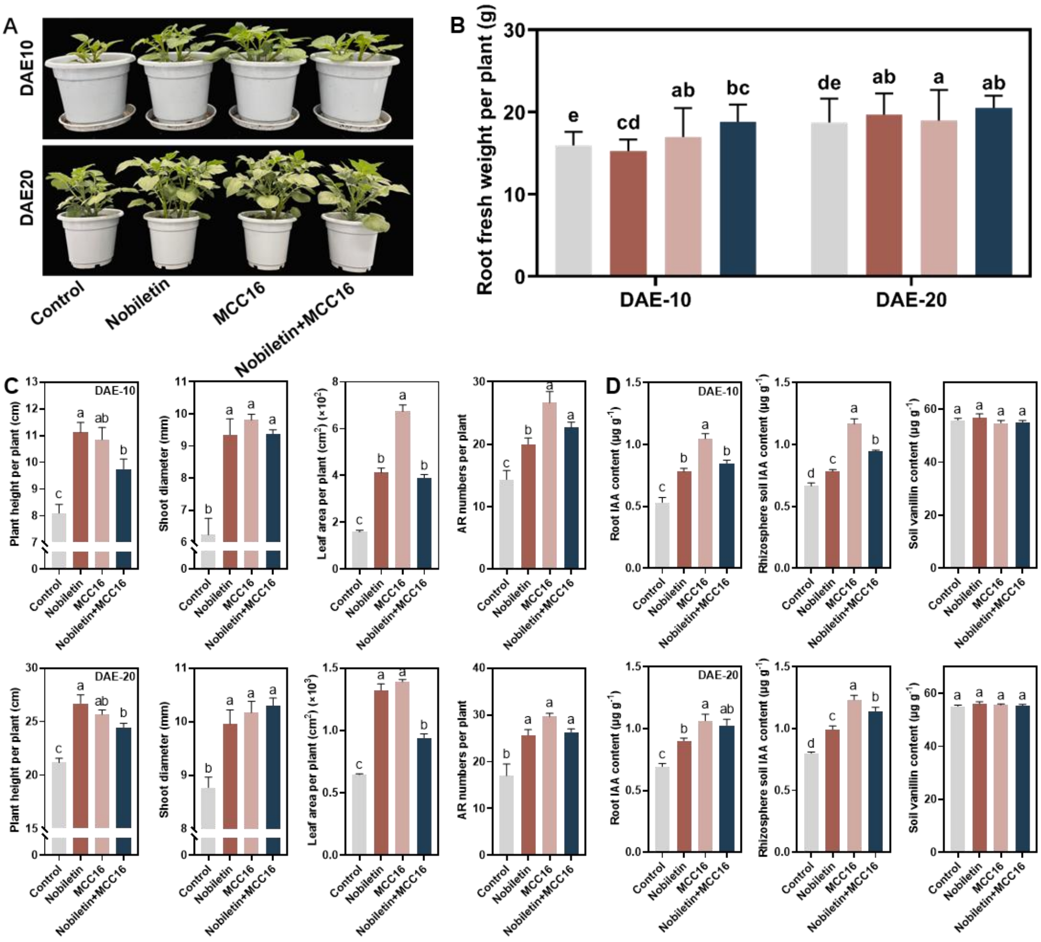

Supplementary Figure 10. Alleviating effects of exogenous nobiletin and *Pantoea* sp. MCC16 on continuous cropping potato.

(A) Effects of exogenous nobiletin or *Pantoea* sp. MCC16 on the growth of continuous cropping potato plants

(B) Effects of exogenous nobiletin or *Pantoea* sp. MCC16 on the root fresh weight of continuous cropping potato plants

(C) Effects of exogenous nobiletin or *Pantoea* sp. MCC16 on the plant height, shoot diameter, leaf area and adventitious root (AR) numbers of continuous cropping potato plants

(D) Effects of exogenous nobiletin or *Pantoea* sp. MCC16 on root IAA content, rhizosphere soil IAA content, and rhizosphere soil vanillin content.

Data are shown as the mean ± SEM (n = 3). Different letters indicate significant differences (Tukey's HSD test, $p < 0.05$).

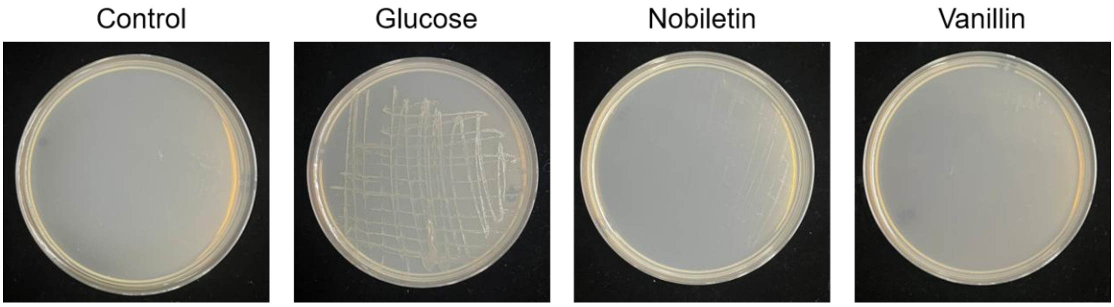

Supplementary Figure 11. Carbon source identification of *Pantoea* sp. MCC16.

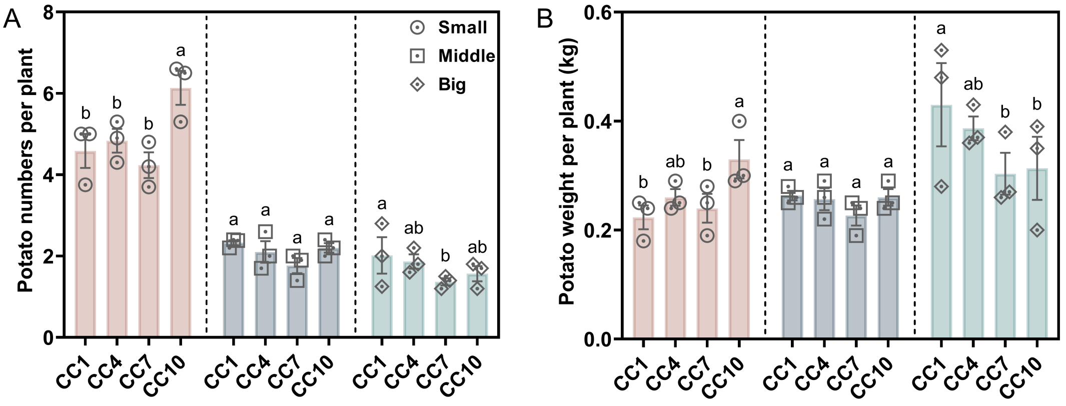

Supplementary Figure 12. Yield composition of continuous cropping potato in the field.

(A) Number of potatoes per plant under different continuous cropping years.

(B) Potato weight per plant under different continuous cropping years.

CC-X: potato continuous cropping for X years. Small: < 80 g, Middle: 80–200 g, Big: > 200 g. Data are shown as the mean ± SEM (n = 3). Different letters indicate significant differences (Tukey's HSD test, $p < 0.05$).

Supplementary Tables

Supplementary Table 1. List of qRT-PCR primers used in this study.

Gene	Primer name and sequence (5´-3´)	PCR protocol
StEF-1α	Forward, CTTGTACACCACGCTAAGGAG<br>Reverse, GTCAATGCAAACCATTCCTTG	95°C for 1 min; followed by 95°C for 10 s, 60°C for 20 s, 40 cycles
StYUCCA5	Forward, TCCATCTTGGCTACAGGAAAGTGA<br>Reverse, TGCAACGTCTGTGTGTTGGAACT	
StTAR2	Forward, GGAGAATGCAACTTCAGCCATCA<br>Reverse, TCACGGAGACTCCACGGAGT	
StARF3	Forward, TGTTGTAGGTATCTCACATCTGGTTCACA<br>Reverse, TCTAGACCAAGTGAGTACACAGCTTCA	
StIAA4	Forward, AGAAGCTGAGTGTGGAATGTATGTGA<br>Reverse, TCTCCAACAAGCATCAAGTCACCA	

Supplementary Table 2 Identification of the IAA production capacity of all rhizosphere soil isolates during the potato seedling stage. " + " and " - " indicate that the color reaction is positive (bolded text) and negative (plain text), respectively.

DAE-10		DAE-20		DAE-30	
Strain ID	IAA-production	Strain ID	IAA-production	Strain ID	IAA-production
INCC1	-	MNCC1	-	FNCC1	-
INCC2	-	MNCC2	-	FNCC2	-
INCC3	-	**MNCC3**	+	FNCC3	-
INCC4	-	MNCC4	-	FNCC4	-
INCC5	-	**MNCC5**	+	**FNCC5**	+
INCC6	-	**MNCC6**	+	FNCC6	-
INCC7	-	MNCC7	-	FNCC7	-
INCC8	-	MNCC8	-	FNCC8	-
**INCC9**	+	MNCC9	-	FNCC9	-
**INCC10**	+	MNCC10	-	FNCC10	-
INCC11	-	MNCC11	-	FNCC11	-
INCC12	-	MNCC12	-	FNCC12	-
ICC1	-	MNCC13	-	FNCC13	-
**ICC2**	+	MNCC14	-	**FNCC14**	+
ICC3	-	MNCC15	-	FNCC15	-
ICC4	-	MNCC16	-	FNCC16	-
		**MNCC17**	+	**FNCC17**	+
		MNCC18	-	**FNCC18**	+
		MNCC19	-	FNCC19	-
		MNCC20	-	FNCC20	-
		MNCC21	-	**FNCC21**	+
		MNCC22	-	FNCC22	-
		MNCC23	-	FCC1	-
		MNCC24	-	FCC2	-

MNCC25	-	FCC3	-
MNCC26	-	**FCC4**	+
**MNCC27**	+	FCC5	-
MNCC28	-	FCC6	-
MNCC29	-	**FCC7**	+
MNCC30	-	FCC8	-
MNCC31	-	FCC9	-
MNCC32	-	FCC10	-
MNCC33	-	**FCC11**	+
MCC1	-	FCC12	-
MCC2	-	FCC13	-
MCC3	-	FCC14	-
MCC4	-	FCC15	-
MCC5	-		
MCC6	-		
MCC7	-		
MCC8	-		
MCC9	-		
MCC10	-		
MCC11	-		
MCC12	-		
MCC13	-		
MCC14	-		
MCC15	-		
**MCC16**	+		
MCC17	-		
MCC18	-		
MCC19	-		

Supplementary Table 3. Root exudates of the top 10 KEGG pathways enriched in CC20.

Treatment	KEGG ID	KEGG pathway	Root exudates enriched in CC20
CC20 vs CC10 NEG	ath00220	Arginine biosynthesis	Fumaric acid<br>trans-Cinnamic acid<br>L-Aspartic acid<br>Uridine<br>Thymidine<br>L-Saccharopine<br>Phenylacetaldehyde<br>Dopamine<br>Leukotriene C4
	ath00240	Pyrimidine metabolism	
	ath00360	Phenylalanine metabolism	
	ath00965	Betalain biosynthesis	
	ath00590	Arachidonic acid metabolism	
	ath01040	Biosynthesis of unsaturated fatty acids	
	ath00901	Indole alkaloid biosynthesis	

	ath00950	Isoquinoline alkaloid biosynthesis	L-Ornithine<br>2'-Deoxycytidine<br>Tryptamine
	ath00310	Lysine degradation	
	ath00250	Alanine, aspartate and glutamate metabolism	
CC20 vs NCC20 NEG	ath00500	Starch and sucrose metabolism	Citric acid<br>Sucrose<br>α,α-Trehalose<br>Stachyose<br>Raffinose<br>dUMP<br>Pheophorbide A<br>Trehalose 6-phosphate<br>alpha-Ketoglutaric acid
	ath00052	Galactose metabolism	
	ath00020	Citrate cycle (TCA cycle)	
	ath00940	Phenylpropanoid biosynthesis	
	ath00240	Pyrimidine metabolism	
	ath00250	Alanine, aspartate and glutamate metabolism	
	ath00630	Glyoxylate and dicarboxylate metabolism	
	ath00270	Cysteine and methionine metabolism	
	ath00860	Porphyrin metabolism	
	ath00230	Purine metabolism	
CC20 vs CC10 POS	ath00670	One carbon pool by folate	**4-Guanidinobutyric acid**<br>**L-Glutamic acid**<br>**L-Pyroglutamic acid**<br>S-Adenosyl-L-homocysteine<br>(5-L-Glutamyl)-L-amino acid<br>L-Homoserine<br>**Kaempferol**<br>Homocysteine<br>Folinic acid<br>Folic acid
	ath00330	Arginine and proline metabolism	
	ath00480	Glutathione metabolism	
	ath00965	Betalain biosynthesis	
	ath00750	Vitamin B6 metabolism	
	ath00944	Flavone and flavonol biosynthesis	
	ath00250	Alanine, aspartate and glutamate metabolism	
	ath00270	Cysteine and methionine metabolism	
	ath00220	Arginine biosynthesis	
	ath00073	Cutin, suberine and wax biosynthesis	
CC20 vs NCC20 POS	ath00480	Glutathione metabolism	**4-Guanidinobutyric acid**<br>Serotonin<br>**L-Glutamic acid**<br>Pyridoxine<br>Indole-3-acetic acid<br>**L-Pyroglutamic acid**<br>D-Xylulose<br>3-Hydroxyanthranilic acid<br>Pyridoxal<br>**Kaempferol**<br>Glutathione<br>L-Citrulline<br>Vitamin C
	ath00750	Vitamin B6 metabolism	
	ath00950	Isoquinoline alkaloid biosynthesis	
	ath00944	Flavone and flavonol biosynthesis	
	ath00250	Alanine, aspartate and glutamate metabolism	
	ath00380	Tryptophan metabolism	
	ath00040	Pentose and glucuronate interconversions	
	ath00053	Ascorbate and aldarate metabolism	
	ath00330	Arginine and proline metabolism	
	ath00220	Arginine biosynthesis	
