# Supplementary material for: Self-alleviation of continuous-cropping obstacles in potato via root-exudate-driven recruitment of growth-promoting bacteria
Source: Plant Commun. 2025 May 12;6(7):101372. doi: 10.1016/j.xplc.2025.101372 (PMC12281297; doi:10.1016/j.xplc.2025.101372)
Supplement: Document S2. Article plus supplemental information [file mmc2.pdf]

Plant Communications
Research article

CellPress Partner Journal

Self-alleviation of continuous-cropping obstacles in potato via root-exudate-driven recruitment of growth-promoting bacteria

Haiyan Ma[1,2,5,6], Zhitong Ren[1,2,6], Aihua Luo[3,6], Xiaoting Fang[1,2], Ruilin Liu[1,2], Chao Wu[1,2], Xinxin Shi[1,2], Junji Li[1,2], Heping Lv[3], Xiaohua Sun[3], Kaiqin Zhang[1,2] and Shunlin Zheng[1,2,4,*]

[1]State Key Laboratory of Crop Gene Exploration and Utilization in Southwest China, College of Agronomy, Sichuan Agricultural University, Chengdu 611130, China

[2]Crop Ecophysiology and Cultivation Key Laboratory of Sichuan Province, Chengdu 611130, China

[3]Potato Research Institute of Gansu Academy of Agricultural Sciences, Lanzhou 730070, China

[4]Key Laboratory of Tuber Crop Genetics and Breeding, Ministry of Agriculture, Chengdu Joyson Agricultural Technology Co., Ltd, Xindu 610500, China

[5]Yibin Academy of Agricultural Sciences, Yibin 644699, China

[6]These authors contributed equally to this article.

***Correspondence: Shunlin Zheng (zhengshunlin123@163.com)**

https://doi.org/10.1016/j.xplc.2025.101372

ABSTRACT

Continuous-cropping (CC) obstacles are among the primary factors limiting the development of global agriculture. Although most plants are negatively affected by barriers that develop with CC, they may also overcome such obstacles by altering the soil biological and chemical environment to favor plant growth. In this study, we investigated the mechanism by which plants recruit beneficial microorganisms using root exudates to alleviate obstacles in a 10-year CC potato system. On day 20 after potato emergence, soil microorganisms in the CC system promoted an increase in adventitious root (AR) numbers by increasing the indole-3-acetic acid (IAA) content of the rhizosphere soil. Analysis of rhizosphere bacterial communities using 16S rRNA sequencing revealed that CC alters community structure, increasing the abundance of *Pantoea* sp. MCC16. Irrigation with root exudates from CC potato significantly increased AR numbers and *Pantoea* sp. MCC16 abundance. Through untargeted metabolomic analysis, we identified nobiletin as key metabolite that promotes *Pantoea* sp. MCC16 colonization in the rhizosphere. Furthermore, application of either nobiletin or *Pantoea* sp. MCC16 significantly improved the yield of CC potatoes. These findings demonstrate that CC plants can actively secrete the unique metabolite nobiletin to recruit *Pantoea* sp. MCC16, a high IAA producer, to help plants recover functional traits and mitigate CC obstacles.

Key words: potato, continuous cropping, *Pantoea* sp. MCC16, nobiletin, adventitious root numbers

Ma H., Ren Z., Luo A., Fang X., Liu R., Wu C., Shi X., Li J., Lv H., Sun X., Zhang K., and Zheng S. (2025). Self-alleviation of continuous-cropping obstacles in potato via root-exudate-driven recruitment of growth-promoting bacteria. Plant Comm. **6**, 101372.

INTRODUCTION

Although the traditional practice of continuous cropping (CC) can meet short-term yield demands, the prolonged use of CC engenders a complex of persistent challenges, collectively termed CC obstacles. CC obstacles significantly impair crop productivity and sustainability, thereby hindering the long-term development of modern agriculture (Li et al., 2021; Ma et al., 2023a).

Geographic constraints, arable-land limitations, economic demands, and intensified modern farming practices reduce species diversity in continuously cropped areas. Combined with excessive fertilizer application aimed at maximizing crop yields, these factors disrupt ecosystem self-regulation, exacerbating CC obstacles. It is challenging to completely eliminate such obstacles when implementing a CC system. However, artificial means can be used to mitigate the suppression of plant growth in CC systems. The main obstacles to CC are usually caused by soil-borne diseases and autotoxicity. Approaches to prevent and control soil-borne diseases in CC systems have evolved from extensive management aimed at eliminating all soil microorganisms, to more refined management focusing on individual pathogenic bacteria, and finally to management of the entire microbial community, which involves making overall adjustments to

© 2025 The Authors. Published by Elsevier Inc. on behalf of CAS Center for Excellence in Molecular Plant Sciences, Chinese Academy of Sciences, and Chinese Society for Plant Biology. This is an open access article under the CC BY license (http://creativecommons.org/licenses/by/4.0/).

soil structure (Wang et al., 2024a). The introduction of microorganisms with specific functions has historically been the primary technique used to regulate soil microbial community structure. However, soil colonization is challenging because of factors such as poor environmental adaptability, competition with native flora, and host immune responses. Therefore, an effective strategy to prevent and control soil-borne diseases under CC conditions is the direct soil application of prebiotics required by beneficial bacteria to increase their competitiveness or the application of functional strains that can inhibit the main active substances of pathogenic bacteria (Chung et al., 2016; Wen et al., 2023). In addition, ecosystem function can be effectively optimized by leveraging the "home-field advantage" of native beneficial microorganisms (Jiang et al., 2023).

Autotoxicity, mainly mediated by phenolic acids produced by the plants themselves, remains a difficult problem. Small amounts of phenolic acids can temporarily improve plant tolerance and reduce oxidative stress (Martinez et al., 2016; Sun et al., 2024). However, long-term CC leads to the accumulation of large amounts of phenolic acids in the soil, which disrupt root membrane structures and hinder the development of root morphology (Ni et al., 2021). Autotoxicity is most commonly observed during the seedling stage, although the specific phenolic acids responsible for autotoxicity vary among different plants (Wang et al., 2022). Therefore, to alleviate autotoxicity, in addition to developing new varieties that are resistant to CC obstacles, a common practice is to screen for beneficial bacteria that can degrade specific phenolic acids on a large scale and then apply them to CC soil in a targeted manner (Chen et al., 2023). However, with this method, challenges remain in terms of colonization and efficacy. Artificial stimulation of root system establishment during the seedling stage is another approach to alleviate autotoxicity, and it has been shown to increase yield by approximately 20% (Ma et al., 2023b). With this method, negative plant responses to external stress weaken, gradually reducing the release of phenolic acids into the soil.

Although CC leads to an imbalance in overall ecosystem function, plants can still promote their own growth by adjusting surrounding microhabitats in a "cry-for-help" strategy that is common in plant–microbe systems (Rolfe et al., 2019). Plants actively secrete metabolites that attract beneficial bacteria to accumulate in the rhizosphere. The specific metabolites serve as signaling or energy substances for the bacteria, with specific bacteria selectively recruited during certain periods and their functions maintained. Under long-term stress conditions, nonresistant plant varieties tend to be more effective at stimulating soil microbial communities to suppress pathogens and alleviate environmental stresses—a phenomenon indicative of the development of resistant soil—compared with their resistant counterparts, although the extent of this effect varies depending on the specific environmental conditions and the particular plant–microbe interactions involved (Raaijmakers and Mazzola, 2016). For example, susceptible varieties of cucumber secrete more organic acids to recruit Comamonadaceae, which inhibit *Fusarium oxysporum* f.sp. *cucumerinum* (Foc) (Wen et al., 2020). Similarly, after infection by *Pseudomonas syringae* pv *tomato* (Pst), the subsequent generation of *Arabidopsis* secretes more long-chain organic acids and amino acids to regulate the soil bacterial community and increase plant resistance (Yuan et al., 2018). Some of the obstacles posed by CC can be alleviated or even eliminated with time, usually more than 10 years (Zhang and Chen, 2014), suggesting that plants develop strategies to cope with CC obstacles over the long term. Such strategies are closely linked to the "root exudate–soil microbial" system. Therefore, harnessing the restoration capabilities of the ecosystem itself may be the most effective approach for mitigation of CC obstacles.

Potatoes, a top-four food crop, are known for their nutrient-rich and stress-resistant properties and are widely grown in CC systems. However, the inherent intolerance of Solanaceae plants to CC makes the CC of potato a persistent industry challenge (Chen et al., 2023), and potato seedling growth is typically significantly inhibited in CC systems. In the absence of noticeable pest and disease symptoms, the accumulation of autotoxic substances in soil is thought to be a primary factor underlying this phenomenon (Guo et al., 2016; Lyu et al., 2022; Xiang et al., 2022). In previous research, we discovered that the autotoxic substance vanillin, present in CC systems, initially led to significantly lower adventitious root (AR) numbers than those of normal plants (Ma et al., 2023b). However, AR numbers increased significantly later in the seedling stage. We hypothesized that long-term CC stimulates plants to autonomously recruit beneficial microorganisms, thereby increasing AR numbers. Changes in potato root exudates are considered to drive increases in beneficial plant microorganisms, with exudates influenced by autotoxic substances in CC soil. To test this hypothesis, we performed experiments to (1) evaluate the establishment of a microbiome that promotes increased AR numbers in a CC system, (2) analyze root exudates of CC potatoes to identify signaling compounds for the recruitment of beneficial bacterial taxa, and (3) examine changes in plant-growth-promoting rhizosphere bacteria after exposure to root exudates from CC potatoes and examine the effect of using plant-growth-promoting bacteria and growth-promoting metabolites to alleviate CC obstacles (Supplemental Figure 1).

The results of this study shows that AR numbers increased significantly at a later seedling stage under vanillin stress in CC conditions. The increase in AR numbers was due to significant enrichment of *Pantoea* sp. MCC16 in the rhizosphere soil. By increasing the indole-3-acetic acid (IAA) content of rhizosphere soil, the bacterium increased the content of root IAA, which in turn promoted increases in AR. In addition, we found that enrichment of *Pantoea* sp. MCC16 was due to the recruitment ability of nobiletin, which was secreted by CC potato roots under autotoxic conditions.

RESULTS

Autotoxicity inhibits potato plant growth under continuous cropping

CC significantly inhibited potato seedling growth (Figure 1, ① in Supplemental Figure 1). At the seedling stage, AR numbers increased significantly at 10–20 days after emergence (DAE) under noncontinuous cropping (NCC) conditions but at 20–30 DAE under CC conditions. The maximum IAA content occurred at 10 DAE in NCC potato roots but at 20 DAE in CC potato roots (Figure 1A). Although plant height, shoot diameter, and leaf and root dry weights of CC potato increased significantly at 20–30 DAE, these values were always lower than those of NCC

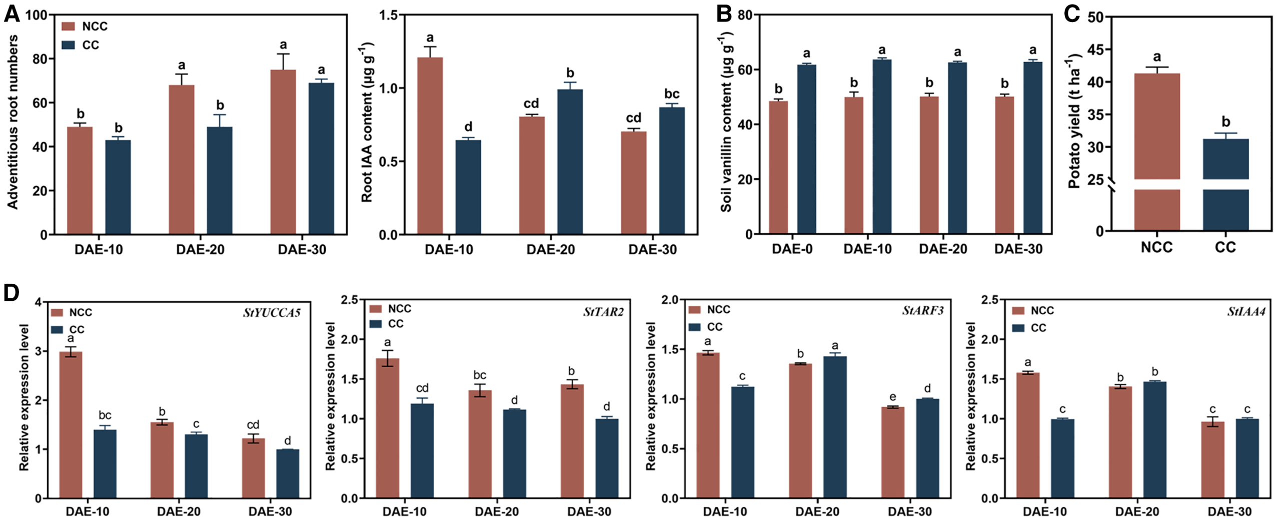

Figure 1. Inhibition of potato plant growth under continuous cropping.
(A) Effects of continuous cropping (CC) on the number of potato ARs and the root indole-3-acetic acid (IAA) content.
(B) Effects of CC on soil vanillin content.
(C) Effects of CC on potato yield.
(D) Effects of CC on the expression of IAA-synthesis (*StYUCCA5*, *StTAR2*) and IAA-response (*StARF3*, *StIAA4*) genes. NCC, noncontinuous cropping; DAE-X, X days after potato emergence. Data are shown as the mean ± SEM (n = 3). Different letters indicate significant differences (Tukey's HSD test, $p < 0.05$).

potato, and potato yield was significantly lower (−24.43%) under CC conditions than under NCC conditions (Supplemental Figure 2; Figure 1C). In rhizosphere soil at the seedling stage, the vanillin content of CC soil was significantly higher than that of NCC soil on all sample dates (Figure 1B). According to RT–qPCR analysis, CC significantly inhibited expression of the root IAA-synthesis genes *StYUCCA5* and *StTAR2* throughout the seedling stage, whereas expression of the root IAA-response genes *StARF3* and *StIAA4* increased significantly at 10–20 DAE (Figure 1D). These results indicated that CC significantly inhibited the growth of potato seedlings but that substances in CC soil promoted an increase in root IAA content at 20 DAE.

Soil microorganisms promotes potato plant growth under continuous cropping

To determine whether microorganisms in CC soil could promote plant growth, we set up a soil sterilization test. In CC soil, the presence of microorganisms promoted potato plant growth (Supplemental Figure 3A). Soil vanillin content was significantly higher in CC soil than in NCC soil, regardless of whether the CC soil had been sterilized, indicating the development of autotoxicity in CC soil (Supplemental Figure 3D). Soil sterilization had no significant effect on AR numbers at any seedling stage in NCC soil, and AR numbers increased significantly at 10–20 DAE (Figure 2A). In addition, IAA content did not differ significantly between sterilized and unsterilized NCC soil during the seedling stage, and root IAA content was highest at 10 DAE (Supplemental Figures 3B and 3C).

In CC soil, AR numbers in sterilized soil did not increase significantly during the seedling stage, but in unsterilized soil, numbers increased significantly at 20–30 DAE (Figure 2A). In CC rhizosphere soil, IAA content increased significantly at 20 DAE (Supplemental Figure 3C). Notably, expression of the root IAA-synthesis genes *StYUCCA5* and *StTAR2* decreased significantly, whereas expression of the root IAA-response genes *StARF3* and *StIAA4* increased significantly (Figure 2B). In addition, although potato yield was significantly lower in CC soil than in NCC soil (regardless of sterilization), potato yield was significantly lower in sterilized continuously cropped soil (CCS) than in in unsterilized CC soil (Figure 2A). These results indicated that soil microorganisms caused the increase in AR numbers under CC conditions (with autotoxicity) (② in Supplemental Figure 1).

To further explore the stage at which microorganisms in CC soil played the main role in increasing AR numbers, we collected rhizosphere flora of potato seedlings in CC and NCC soil and transferred them to sterilized CC soil. There was no significant difference in the effect of bacterial transfer on soil vanillin content, and vanillin stress was present in each treatment (Supplemental Figure 3G). Soil microorganisms from NCC soil did not significantly affect the IAA content of rhizosphere soil or potato roots, whereas soil microorganisms from CC soil significantly increased IAA content in both soil and roots. Regarding the increases in IAA content in rhizosphere soil and roots, the most significant effect of CC rhizosphere microorganisms occurred at 20 DAE (Supplemental Figure 3E and 3F).

In addition, microorganisms from the rhizosphere of NCC potato seedlings produced no significant increases in AR numbers or potato yield. However, microorganisms from CC soil at 20 DAE significantly increased AR numbers throughout the seedling stage, with the first significant difference detected at 10 DAE (Figure 2C). Therefore, microorganisms from CC soil at 20 DAE significantly increased the yield of CC potatoes (Figure 2C, ③ in Supplemental Figure 1). On the basis of these results, we

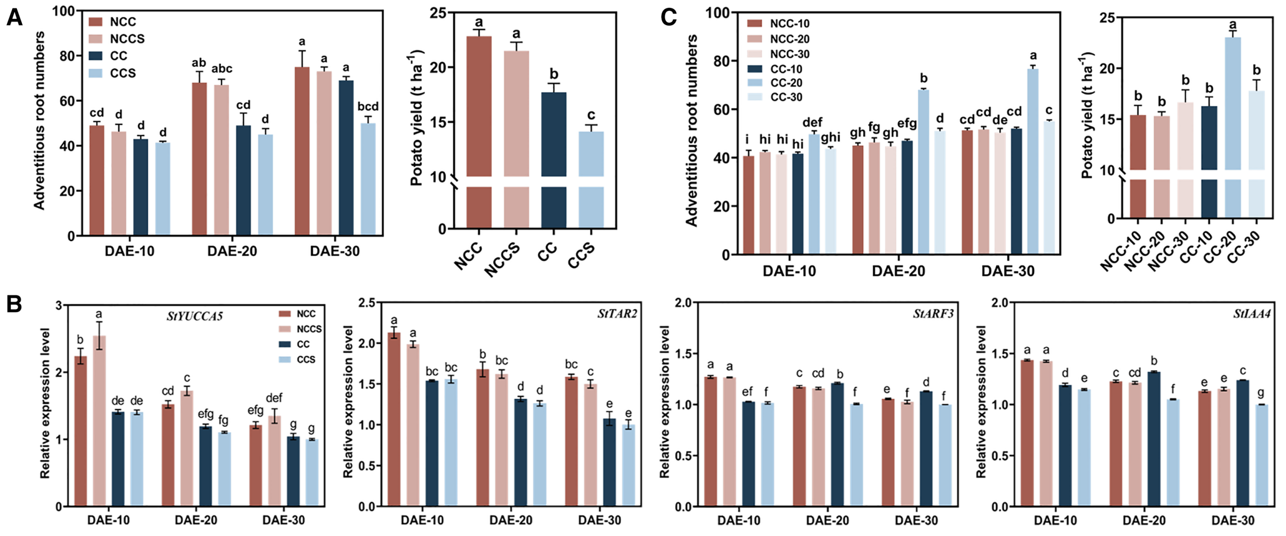

Figure 2. Rhizosphere microorganisms promote potato plant growth in continuously cropped soil 20 days after emergence.
(A) Effects of soil sterilization on the AR number and yield of potato. NCC, unsterilized noncontinuous-cropping soil; NCCS, sterilized noncontinuous-cropping soil; CC, unsterilized continuous-cropping soil; CCS, sterilized continuous cropping soil; DAE-X, X days after potato emergence.
(B) Effects of soil sterilization on the expression of IAA-synthesis (*StYUCCA5*, *StTAR2*) and IAA-response (*StARF3*, *StIAA4*) genes in potato roots.
(C) Effects of microflora transfer on AR numbers and potato yield. NCC-X, rhizosphere microorganisms on day X after potato emergence in noncontinuous cropping soils; CC-X, rhizosphere microorganisms on day X after potato emergence in CC soils.
Data are shown as the mean ± SEM (n = 3). Different letters indicate significant differences (Tukey's HSD test, $p < 0.05$).

hypothesized that microorganisms in CC soil could significantly increase potato yield under CC vanillin stress, with the main functions of rhizosphere microorganisms apparent at 20 DAE of CC potatoes.

Pantoea sp. MCC16 promotes potato growth under continuous cropping

After confirming that rhizosphere soil microorganisms at 20 DAE had a significant growth-promoting effect on CC potatoes, we analyzed the bacterial community structure of CC and NCC soil by performing sequencing for absolute quantification of 16S rRNA at 10 and 20 DAE.

The difference in rhizosphere bacterial community structure between CC and NCC was more significant at 20 DAE than at 10 DAE (Figure 3A). Bacterial community diversity was significantly higher in CC soil than in NCC soil, especially at 20 DAE, whereas there was little difference in community richness (Supplemental Figure 4A and 4B). The absolute abundance of the top 10 bacterial phyla was highest in CC soil at 20 DAE (CC20), especially for Proteobacteria and Bacteroidetes (Figure 3B). To identify which bacteria in CC20 caused significant differences compared with the other treatments, we identified the bacteria that were present in CC20 but not in CC10, NCC10, or NCC20. Eleven bacteria were screened in total, and *Rudaea* (3ASV), *Pantoea* (1ASV), and *Melioribacter* (2ASV) were present only in the CC20 treatment; *Rudaea* and *Pantoea* are members of Proteobacteria (Figure 3C).

Because previous experiments confirmed that the bacteria in CC20 rhizosphere soil could increase soil IAA content, we isolated and cultured rhizosphere soil bacteria in the seedling stage under CC and NCC. Seven IAA-producing strains (IAA-producing ability >20 μg ml^{-1}) were isolated: three strains in CC and four strains in NCC. The IAA production capacity of MCC16 in CC was as high as 77.022 μg ml^{-1}, which was 125.5% higher than that of FNCC5, the strain with the highest IAA production capacity in NCC (Figure 3D). The seven strains were then identified by analyzing their 16S rDNA sequences. Five of the strains were in Firmicutes, whereas the other two were in Proteobacteria (ICC5, MCC16), and the abundance of Proteobacteria in CC20 increased significantly (Figure 3B and 3D). Strain ICC5 was identified as *Escherichia coli*, and strain MCC16 showed 100% homology to *Pantoea* sp. SAS9 (Figure 3E). Absolute quantification of 16S rRNA sequencing results revealed that only one taxon, ASV7733, was in the genus *Pantoea*. This taxon was only found at 20 DAE, and strain MCC16 was isolated from potatoes at 20 DAE, indicating that ASV7733 was the MCC16 strain (Figure 3E; Supplemental Figure 4C, ④ in Supplemental Figure 1).

When determining whether *Pantoea* sp. MCC16 could promote plant growth in CC potato, we found that the IAA content in rhizosphere soil and roots of CC potato increased significantly after bacterial suspension treatment, leading to increases in AR numbers and potato yield. Notably, expression of the IAA-response genes *StARF3* and *StIAA4* was significantly enhanced in the roots after *Pantoea* sp. MCC16 treatment, whereas expression of the IAA-synthesis genes *StYUCCA5* and *StTAR2* showed no significant change (Figure 3F; Supplemental Figure 4D and 4E). These results also confirmed that *Pantoea* sp. MCC16 was the main strain that promoted plant growth in CC soil (⑤ in Supplemental Figure 1).

Potato root exudates under continuous cropping promote plant growth

To determine why *Pantoea* sp. MCC16 appeared only in CC20, we collected root exudates of CC and NCC potatoes at 10 and

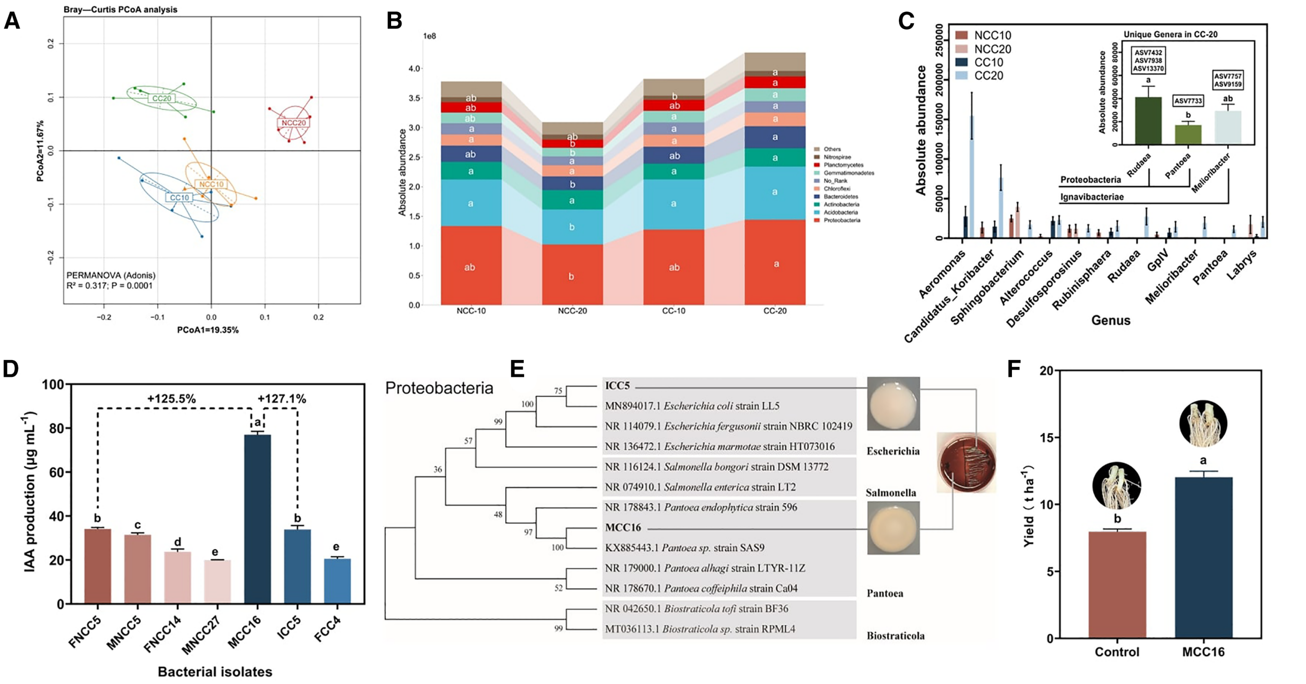

Figure 3. ***Pantoea*** **sp. MCC16 promotes plant growth in continuous cropping soil.**

(A) Principal coordinate analysis of differences in rhizosphere soil community structure under continuous cropping (CC) and noncontinuous cropping (NCC) conditions on days 10 and 20 after emergence. NCC-X, rhizosphere microorganisms on day X after potato emergence in NCC soils; CC-X, rhizosphere microorganisms on day X after potato emergence in CC soils.

(B) Absolute abundance of the top 10 bacterial phyla in CC10, CC20, NCC10, and NCC20.

(C) Bacterial genera absent (11) in CC10, NCC10, or NCC20 and unique genera (3) in CC20.

(D) Strains with an indole-3-acetic acid (IAA) production capacity >20 μg ml^{-1} in CC and NCC rhizosphere soils. I-X, 10 days after potato emergence (DAE); M-X, 20 DAE; F-X, 30 DAE.

(E) Evolutionary tree of bacterial species ICC5 and MCC16. The red plate contains *E. coli* identification medium.

(F) Effect of irrigation with a solution of *Pantoea* sp. MCC16 on the yield of CC potato.

Data are shown as the mean ± SEM (n = 3). Different letters indicate significant differences (Tukey's HSD test, $p < 0.05$).

20 DAE and performed irrigation tests of root exudates against the background of unsterilized CC soil. Only the CC20 root exudates significantly increased AR numbers at the beginning of the potato seedling stage and maintained this enhancement effect over time. In addition, strain MCC16 in rhizosphere soil appeared only under irrigation with CC20 root exudates at 10 DAE (Figure 4A). These results indicated that CC20 root exudates could directly promote *Pantoea* sp. MCC16 colonization of the potato rhizosphere (⑥ in Supplemental Figure 1).

To further explore which specific metabolites in CC20 could recruit *Pantoea* sp. MCC16, we performed untargeted metabolomics analysis of root exudates in CC and NCC. The components and contents of root exudates treated with CC and NCC differed significantly in both positive and negative modes (Figure 4B). Using Kyoto Encyclopedia of Genes and Genomes (KEGG) pathway enrichment analysis (TOP10) (Supplemental Figure 5; Supplementary Table 3), principal-component analysis variable-importance analysis (TOP40) (Supplemental Figure 6), random forest variable importance analysis (TOP40) (Supplemental Figure 7), and significant difference analysis (TOP50) (Supplemental Figure 8), we selected 29 metabolites that increased significantly only in CC20 (Figure 4C, ⑦ in Supplemental Figure 1). The absolute abundance of MCC16 was significantly positively correlated with the relative contents of 11 metabolites: N-isovalerylglycine, epimedin C, D-turanose, nobiletin, sauchinone, praeruptorin A, isomucronulatol-7-O-glucoside, methyl 4-hydroxy-3-methoxycinnamate, 3-succinoylpyridine, thioctic acid, and kaempferol (Figure 4D).

Nobiletin alleviates potato continuous cropping obstacles by recruiting *Pantoea* sp. MCC16

To determine which substances could recruit *Pantoea* sp. MCC16, we performed an *in vitro* test to assess the chemotaxis of *Pantoea* sp. MCC16 toward the 11 substances. Only nobiletin significantly stimulated chemotaxis in *Pantoea* sp. MCC16, and this chemotaxis reached its maximum value at 20 μmol l^{-1} nobiletin (Figure 5A). We next examined the effect of nobiletin on biofilm formation by *Pantoea* sp. MCC16 and found that biofilm formation was significantly promoted by 20 μmol L^{-1} nobiletin (Figure 5B, ⑧ in Supplemental Figure 1). To confirm this result, we set up an experiment with exogenous nobiletin irrigation in CC soil. In CCS, exogenous nobiletin had little effect on AR numbers. In unsterilized CC soil, however, nobiletin significantly promoted AR numbers, increasing them by 73.91% at DAE-10 and 125.49% at DAE-20 compared with CCS (Supplemental Figure 9). Finally, we set up an experiment with exogenous nobiletin irrigation in CC soil. Compared with CC or CCS control soil, nobiletin significantly increased the abundance of

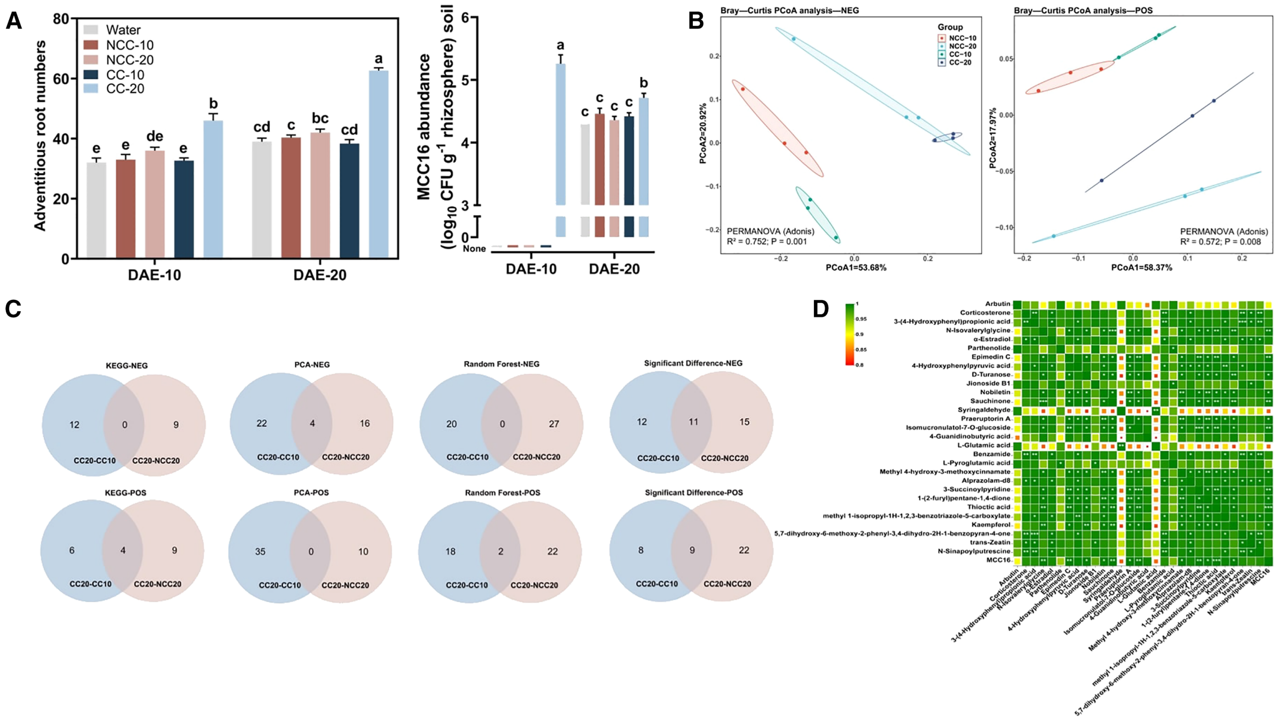

Figure 4. *Pantoea* sp. MCC16 colonization was stimulated by root exudates on day 20 after emergence of continuously cropped potato.

(A) Effects of root exudates from CC and NCC potato on AR numbers and *Pantoea* sp. MCC16 abundance of CC potato. NCC-X, root exudates on day X after potato emergence in NCC soils; CC-X, root exudates on day X after potato emergence in CC soils; CFU, colony-forming unit.

(B) Principal coordinate analysis of root exudates in CC and NCC.

(C) Four methods used to screen for metabolites that were concentrated in the root exudates of CC20.

(D) Correlation analysis of metabolites enriched in CC20 (29 metabolites) and *Pantoea* sp. MCC16.

Data are shown as the mean ± SEM ($n = 3$). Different letters indicate significant differences (Tukey's HSD test, $p < 0.05$).

Pantoea sp. MCC16 in rhizosphere soil. These results suggested that nobiletin was a major functional substance secreted by CC potato roots that could recruit *Pantoea* sp. MCC16 (Figure 5C, ⑨ in Supplemental Figure 1).

Finally, we performed a pot test with exogenous application of nobiletin and a *Pantoea* sp. MCC16 bacterial solution. Nobiletin and *Pantoea* sp. MCC16 significantly increased AR numbers at 10 and 20 DAE (Figure 5D). In further studies, no significant difference was found in soil vanillin content under different treatments. However, the IAA content of potato rhizosphere soil and roots increased significantly (Supplemental Figure 10). Exogenous application of nobiletin and *Pantoea* sp. MCC16 also significantly increased potato yield, although their modes of action differed. Nobiletin mainly increased potato quantity, whereas *Pantoea* sp. MCC16 mainly increased potato unit weight (Figure 5E, ⑩ in Supplemental Figure 1; Supplemental Figure 12). This difference may have occurred because exogenous nobiletin changed the structure of the rhizosphere microbial community, thus changing microbial community function.

DISCUSSION

Agricultural ecosystems possess an inherent capacity for self-repair, which manifests as the alleviation of growth suppression caused by biotic or abiotic stresses when environmental conditions improve (Ma et al., 2019; Davis et al., 2023). However, the ability of agricultural ecosystems to overcome CC obstacles has not yet been investigated extensively. This failure may be due to plant–soil systems not reaching self-repair thresholds after an insufficient number of CC years (Xuan et al., 2024). In addition, the initial self-repair of plant–soil systems cannot compensate for losses in the final economic yield, leading to the misconception that CC systems lack self-repair ability (Wang et al., 2024a). In this study, even though the final yield of CC potato was significantly lower than that of NCC potato, the roots of CC potato actively recruited beneficial bacteria during the seedling stage to increase rhizosphere IAA content, thereby alleviating the decline in root biomass caused by self-toxic stress and further promoting the morphogenesis of CC potato (Figure 6). Previous studies on CC obstacles have focused primarily on healthy plants that do not show CC obstacles in a small number of fields (Song et al., 2021; Sui et al., 2023). In this study, our findings suggest that the CC system leads to positive regulation by the majority of plants experiencing CC obstacles. Given the prevalence of these plants, the findings of this study may have significant implications for artificial regulation strategies aimed at enhancing plant adaptation to CC environments. In addition, the results revealed a new mechanism by which CC plants autonomously recruit plant-growth-promoting bacteria to alleviate CC obstacles. Thus, this study demonstrates that beneficial microorganisms can

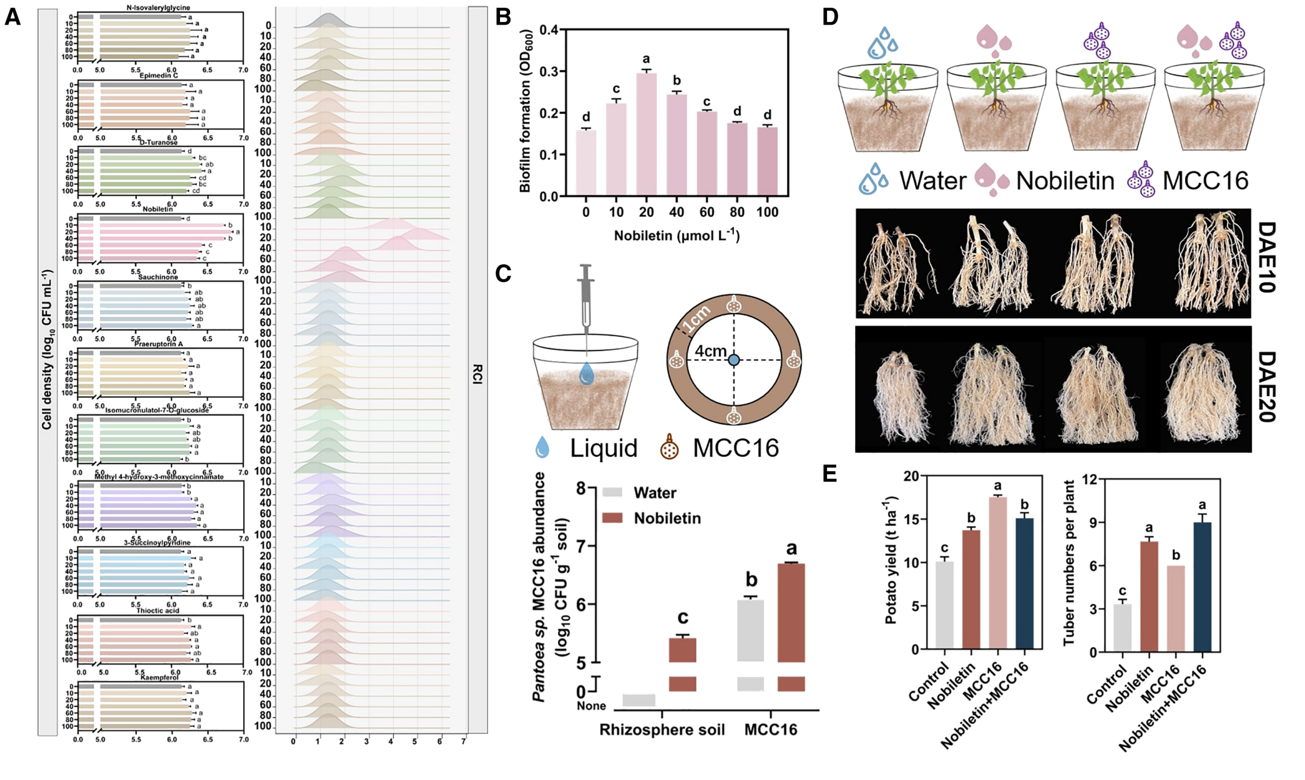

Figure 5. Validation of the ability of nobiletin to alleviate continuous cropping obstacles in potato.

(A) Chemotactic analysis of 11 different concentrations of metabolites and *Pantoea* sp. MCC16.

(B) Effect of different concentrations of nobiletin on the biofilm formation of *Pantoea* sp. MCC16.

(C) Verification of the chemotaxis of *Pantoea sp. MCC16 toward* exogenous metabolites in sterilized CC soil. "Rhizosphere soil" denotes rhizosphere soil inoculated with CC20; "MCC16" denotes inoculation with *Pantoea* sp. MCC16.

(D) Effects of exogenous nobiletin and *Pantoea* sp. MCC16 on the growth of CC potato. Different droplet properties represent different treatments. The blue droplet represents the control with sterile water. DAE, days after emergence.

(E) Effects of exogenous nobiletin and *Pantoea* sp. MCC16 on the yield of CC potato.

Data are shown as the mean ± SEM ($n = 3$). Different letters indicate significant differences (Tukey's HSD test, $p < 0.05$).

be obtained from rhizosphere soil with CC obstacles, providing a new way to overcome difficulties due to CC.

Self-repair in a continuous cropping system dose not directly reduce the content of an autotoxic substance

The accumulation of autotoxic substances is the main factor that causes autotoxicity during CC of plants. To date, most research on mitigating plant autotoxicity has focused on how to eliminate autotoxic substances (Zhang et al., 2010; Chen et al., 2011; Liu et al., 2023). However, according to the results of this study, the ultimate goal of the measures taken by potato plants to deal with autotoxic stress was not to reduce the content of autotoxic substances but to compensate for the plant growth defects caused by autotoxic stress, mainly by increasing AR numbers to promote plant growth at the seedling stage (Figure 1). This strategy may be a sustainable positive regulatory measure chosen by plants independently under long-term autotoxic stress to obtain greater stability. A recent study also demonstrated for the first time that continued allelopathic stress can promote plant microorganisms that have positive effects on plant performance (Revillini et al., 2023). Research also indicates that nonresistant varieties that have been exposed to the same stress for an extended period are more likely to induce soil resistance to the stress, resulting in what is commonly referred to as resistant soil (Raaijmakers and Mazzola, 2016). In addition, when a plant chooses to alleviate its own stress by reducing autotoxic substances in the soil environment, the plant's continued input of those substances into the soil is not affected. Moreover, relying on microorganisms to degrade autotoxic substances over the long term will continue to consume the plant's energy. By contrast, improving the plant's own growth under autotoxic stress can adjust metabolism in the short term, thereby reducing the input of autotoxic substances into the soil and stably improving the soil environment under CC (Xuan et al., 2024).

Under vanillin stress during CC, a decrease in AR numbers due to vanillin inhibition of root IAA content is the main factor that causes reduced root biomass (Ma et al., 2023b). However, under continued vanillin stress in the present study, the IAA content of potato roots increased, the expression of root IAA-responsive genes increased significantly, and the expression of root IAA-synthesis genes decreased significantly (Figure 1D). The results indicated that the significant increase in IAA content in CC potato roots was closely associated with increased IAA content in the soil. Li et al. (2019) observed a significant decrease in the expression of IAA-synthesis and -response genes in the roots

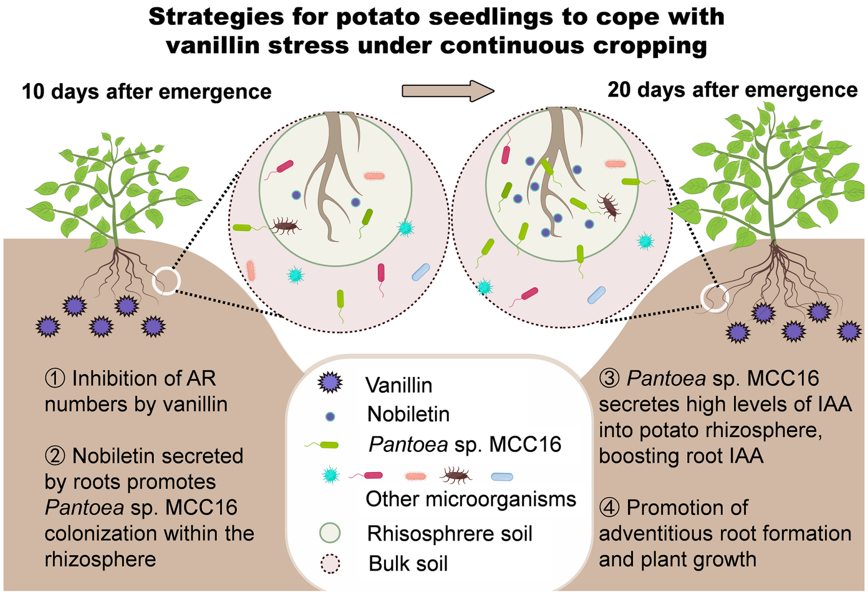

Figure 6. Conceptual model of plant self-repair under continuous cropping obstacles.

(1) When potatoes are replanted into continuous cropping (CC) potato soil with accumulated vanillin, seedlings suffer vanillin stress, resulting in inhibited IAA synthesis in the roots, lower adventitious root (AR) numbers, and suppressed aboveground growth. (2) Under CC vanillin stress, potato plants adopt autonomous adaptive strategies by secreting substantial amounts of nobiletin from roots, which acts as a signaling molecule to recruit *Pantoea* sp. MCC16 for rhizosphere enrichment. (3) In the CC potato rhizosphere, *Pantoea* sp. MCC16 secretes abundant IAA (indole-3-acetic acid), increasing rhizosphere IAA levels and subsequently enhancing IAA accumulation in the roots. (4) Consequently, under vanillin stress, CC potato seedlings exhibit increased AR numbers, promoting aboveground plant growth and ultimately achieving mitigation of CC obstacles.

of CC peanuts. In this study, this decrease may have been attributable to the relatively short duration of CC, during which the system did not reach the threshold at which plants initiate self-repair. Furthermore, although CC potatoes did exhibit self-repair, the increase in AR numbers was delayed by approximately 10 days compared with that of normal plants (Figure 1A; Supplemental Figure 2A). This may have been because, after potato seedlings are stressed by vanillin, they require some time to respond to the recruitment of specific functional microorganisms. Previous studies have also shown that, in addition to the perception of stress signals, the initiation of *in vivo* signal transduction, and the activation of molecular network cascades of gene expression (Yang et al., 2023), plant adaptation to environmental stress also requires host–microbe interaction signaling (Rodriguez et al., 2019; Song et al., 2021). Although insufficient AR numbers in the early stage may be the main reason why the yield of CC potatoes is significantly lower than that of normal plants (Huang et al., 2017), AR numbers are expected to rise as the degree of self-repair gradually increases.

Change in the rhizosphere microbial community promotes root morphogenesis of continuous cropping plants

Rhizosphere microorganisms have vital roles in plant growth, participating in growth and development, resistance to stress, and other processes (Berendsen et al., 2012; Kwak et al., 2018; Zhang et al., 2019). The roles of microorganisms in plant growth extend beyond the current growing season and also help future generations of plants to adjust to the growth environment (Wilkinson, 1997; Wilkinson and Sherratt, 2001; Bakker et al., 2018). In plants that reproduce vegetatively, progeny also receive a considerable proportion of symbiotic bacteria and fungi from the parent plant (Vannier et al., 2018). However, although host genetics can significantly influence the assembly of rhizosphere microbial communities, these effects are weaker than those of environmental factors (Pérez-Jaramillo et al., 2017; Thiergart et al., 2019). In addition, unlike other vegetatively propagated plants, the seed potatoes planted each year are virus free and propagated from tissue-culture seedlings. With this approach, it is not possible to pass the stress memory of potato CC obstacles to offspring. Thus, the most effective strategy for alleviating the CC obstacles encountered during potato cultivation is to develop soil resistance to CC through sustained planting practices. This process enables the soil to acquire resilience in the face of environmental changes, resulting in the formation of resistant soil.

In current agricultural research, greater attention is given to the development of resistant soil formed through the persistent induction of a specific pathogen, in which the soil acquires a strong resistance to that pathogen. This acquisition proceeds from no resistance to significant resistance and is due primarily to significant enrichment of antagonistic bacteria with specific resistance to the pathogen in the soil (Song et al., 2021). However, production problems caused by non-pathogenic factors can also lead to the formation of resistant soil, such as the enhancement of soil recovery capacity by long-term drought stress through changes in soil microbial community function (Canarini et al., 2021). Less research has focused on the induction of resistant soil by long-term CC stress. In this study, rhizosphere microorganisms from CC potatoes increased AR numbers by increasing root IAA content (Figure 2; Supplemental Figure 3). The presence of these microorganisms was likely the result of independent selection by potatoes under long-term CC conditions. Studies have shown that the microbiome selected by allelopathic substances can moderately reduce the plant growth inhibition caused by allelopathy (Revillini et al., 2023). However, the selective effect of root signals on soil microorganisms during plant growth may be stronger than that of a single allelopathic substance (Venturi and Keel, 2016). In addition, plants show dynamic changes in microbial recruitment during different growth stages (Shi et al., 2015; Cordovez et al., 2021; Santoyo, 2022). In this study, the difference in microbial communities between CC and normal potato plants was much smaller at 10 DAE than at 20 DAE. At the same time, the

absolute abundance of rhizosphere microorganisms in CC potato plants increased from 10 to 20 DAE. This result was the opposite of that observed for normal potato plants (Figures 3A and 3B) and may reflect an effect of CC soil on the components and contents of potato root exudates, which in turn increased soil bacterial diversity (Supplemental Figure 4A). However, in contrast to this study, most studies have found that CC significantly reduces bacterial diversity (Tan et al., 2017; Zhao et al., 2020), a result that may be related to the different backgrounds of the plant samples. The CC barriers to plant growth in the present study were caused by autotoxicity rather than disease, which reduced the negative interactions between microorganisms to a certain extent. In addition, owing to the dynamic changes in soil microorganisms, sampling time also has an important effect on the measurement results. We believe that the seedling stage, a critical period for the establishment of plant morphology, is more important than other periods.

Plant–microbe interactions can be long term or short term (Cordovez et al., 2021; Orozco-Mosqueda and Santoyo, 2021; Santoyo, 2022). Previous studies have suggested that short-term interactions with specific probiotic bacteria may contribute to plant health and productivity (Berlanga-Clavero et al., 2020). In the present study, *Pantoea* sp. MCC16 appeared only at 20 DAE in CC potatoes (Figure 3C), indicating that there was a short-term interaction between CC potato plants and *Pantoea* sp. MCC16. This may have been because the IAA required to establish the potato root system reached its peak at the seedling stage, whereas later growth does not require abundant IAA in the soil. However, change in the abundance of *Pantoea* sp. MCC16 is a dynamic process. Although the findings of this study suggest that *Pantoea* sp. MCC16 appears only in CC20, this does not mean that no *Pantoea* sp. MCC16 is present in CC10 and CC30 but rather that the detection of low-abundance strains is limited by sequencing technology and bacterial isolation and culture methods. In addition, beneficial microorganisms can improve plant growth by secreting plant hormones into the surrounding environment and also by affecting plant gene expression to cause physiological changes (Angulo et al., 2022).

Microorganisms in the environment typically alter plant IAA levels through two mechanisms: one is the synthesis of IAA by microorganisms for direct absorption and utilization by the plant, and the other is the induction of IAA production by the plant itself (Etesami and Glick, 2024). Plant absorption of exogenous IAA can occur through diffusion or via transport proteins that facilitate its entry into the root system. The process by which microorganisms stimulate the autonomous production of IAA in plants often involves signaling pathways activated by metabolic byproducts or other compounds from the microorganisms. For instance, rhizobia AS5 and AS55 stimulate the expression of *IAA12* and *YUCCA2* in *Arabidopsis*, leading to an increase in endogenous IAA levels (Sujkowska-Rybkowska et al., 2022). *Lysinibacillus* spp. can promote plant growth by producing IAA and influencing the plant's own IAA production (Pantoja-Guerra et al., 2023). Our previous research found that exogenous IAA can enhance the IAA level of potato roots under vanillin stress in a sterile matrix, demonstrating that the root can use exogenous IAA directly (Ma et al., 2023b). In the present study, the rhizosphere microorganisms of CC potato did not affect the expression of auxin synthesis genes in potato roots under autotoxic stress but did significantly increase the expression of auxin-response genes (Figure 2B), indicating that the increased AR numbers may have been due to direct utilization of IAA produced by MCC16 in the soil. Given that transport of IAA into and out of plant cells involves both intramembrane receptors (such as TIR1/AFBs) and extramembrane receptors (such as ABL and TMK) and that IAA does not directly enter plant cells from the outside, it will be necessary to further investigate the activity of both intramembrane and extramembrane receptors in order to characterize the mechanism by which *Pantoea* sp. MCC16 increases root IAA levels. In addition, although more IAA-producing bacteria were screened in the rhizosphere of normal potato plants, the IAA-producing ability of such bacteria in the rhizosphere of CC potatoes was stronger (Figure 3D). Moreover, similar to previous studies (Zhao et al., 2020), the IAA-producing bacteria screened in the rhizosphere of normal potato plants were all Firmicutes, whereas those in CC soil were all Proteobacteria (Figure 3E; Supplemental Figure 4C). This result may reflect the continuous accumulation of root exudates due to CC, which changed the soil environment and excluded those microorganisms for which the growth conditions were not suitable. Although many studies have reported the potential of *Pantoea* to promote plant growth (Hernández-Forte et al., 2022; Kumar et al., 2022), the genus is not common in CC systems, possibly because of differences in sampling times and CC duration.

Importance of root exudates in self-repair of the plant–soil system

Changes in the composition of root exudates during plant growth lead to corresponding changes in the structure of the rhizosphere microbial community (Shi et al., 2015; Cordovez et al., 2021; Santoyo, 2022). However, when plants are exposed to external environmental stress, the composition and content of root exudates often differ significantly from those observed under normal conditions during the same period (Stringlis et al., 2018; Gong et al., 2023). There has been extensive research on root secretions during the formation of resilient soil. For example, after infection by *P. syringae* pv. *tomato* for five consecutive generations, *Arabidopsis thaliana* secretes increased amounts of long-chain organic acids and amino acids to regulate the soil bacterial community and increase plant resistance (Yuan et al., 2018). Wild soybean (*Glycine soja*) exudes key metabolites, such as purines, to recruit beneficial *Pseudomonas* strains against salt stress (Zheng et al., 2024). In this study, at 20 DAE of CC potatoes, roots increased the secretion of nobiletin to recruit *Pantoea* sp. MCC16 and promote the establishment of seedling morphology (Figures 4D and 5C). The secondary metabolite nobiletin is a member of the polymethoxyflavonoids, a subclass of flavonoids (Kawai et al., 2007). Flavonoids, phenylpropanoids, nitrogenous compounds, and terpenoids are the main categories of root-specific metabolites and play crucial roles in regulating the assembly of specific microbial populations in the rhizosphere (Singh et al., 2023; Wang et al., 2024b), mainly serving as signaling substances or growth substrates in the microbial recruitment process (He et al., 2022). In this study, through preliminary verification, we found that *Pantoea* sp. MCC16 could not use nobiletin as a substrate (Supplemental Figure 11). Therefore, nobiletin may effectively recruit *Pantoea* sp. MCC16 because the bacterium contains flavonoid-sensing

regulators. Plant-derived flavonoids can be sensed by the TetR regulator PhlH in *P. fluorescens 2P24* and act as important signaling molecules, providing a sensing mechanism that facilitates the movement of microorganisms at the root–soil interface and increases their colonization on the root surface (Yu et al., 2020). However, the proliferation of *Pantoea* sp. MCC16 requires nutrients whose availability may be significantly influenced by certain substances in the soil. It is currently unclear whether these substances are produced by the host plant's root exudates or result from changes in soil microbial community structure, potentially leading to an increase in microorganisms whose secreted metabolites support the growth of *Pantoea* sp. MCC16 or to a decrease in microorganisms that inhibit its growth.

The shikimic acid pathway is one of the main secondary metabolic pathways involved in plant responses to external stress, and IAA, nobiletin, and vanillin are all secondary metabolites produced through the shikimic acid pathway in potatoes. Both nobiletin and vanillin are produced by the phenylalanine metabolism pathway, whereas IAA is produced by the tryptophan metabolism pathway. Both pathways use chorismate as the initial precursor for synthesis (Maeda and Dudareva, 2012). In this study, the IAA content of CC potatoes decreased, suggesting that the contents of phenylalanine-pathway products may have increased. In addition, vanillin is synthesized from ferulic acid (Gallage and Møller, 2015), whereas nobiletin is a flavonoid. Synthesis of both ferulic acid and flavonoids relies on a common precursor, p-coumaric acid (Mandal et al., 2010). We found that the nobiletin content of CC potatoes increased significantly, suggesting that vanillin synthesis in these plants may have decreased, consistent with previous results (Ma et al., 2023b). This strategy not only promotes plant growth in the current season but also gradually reduces the amount of autotoxic substances released into the soil, thereby improving the soil environment in subsequent CC seasons. Because self-repair is initiated by promoting plant growth at the seedling stage under CC vanillin stress, this strategy also explains, at least in part, why CC potato plants choose to secrete nobiletin to recruit *Pantoea* sp. MCC16.

Although the findings of this study demonstrate that potato plants adapt to CC obstacles under vanillin stress in the soil, this does not preclude the possibility that soil vanillin content may decrease as the adaptive process progresses. A recent study showed that tobacco adapts to CC obstacles primarily by reducing soil vanillin content (Xuan et al., 2024), in contrast to the results of the present study. This discrepancy may be attributed to the shorter duration of CC in the current study or to variations in microbial community composition across different regions, which lead to distinct optimal strategies for different plants. Furthermore, the findings of this study indicate that *Pantoea* sp. MCC16 is a key functional strain that alleviates potato CC obstacles, nobiletin is the key root exudate that recruits *Pantoea* sp. MCC16, and 20 DAE is a critical period for adaptation to CC obstacles. However, factors such as region, climate, and cultivar may lead to differences in the primary functional strains that help potatoes adapt to CC obstacles, the main root exudates that recruit functional strains, and the critical timing of recruitment. Despite these differences, the underlying mechanism for alleviation of CC obstacles identified in this study remains broadly applicable.

Although self-repair capabilities have been demonstrated in long-term CC systems, this ability cannot be relied upon to compensate for the consequences of inappropriate agricultural practices. In practical production, annual yield losses can severely impact laborers' income and industrial development. To protect and enhance these vital agricultural ecosystems, sustainable agricultural management strategies must be implemented. The direct application of beneficial bacteria to the soil is currently the primary method used in agriculture to regulate plant growth. However, owing to unsuitable soil environments and ecological niches, as well as competition for resources with indigenous microorganisms, beneficial bacteria often fail to effectively colonize the rhizosphere (Alabouvette et al., 2009; Wang et al., 2019). The use of beneficial bacteria from the indigenous microbial community may therefore represent an effective solution. In this study, application of the beneficial bacterium *Pantoea* sp. MCC16 to CC soil significantly increased potato yields by 51.09% compared with the control (Figure 3F). Moreover, in large-scale field production, the direct large-scale application of bacterial inoculants may consume substantial resources. The enrichment of target bacterial strains in the soil at the appropriate time may thus provide a more efficient approach. In this study, *Pantoea* sp. MCC16 only became enriched in the rhizosphere 20 days after the emergence of CC potatoes, which was clearly disadvantageous for early plant development. Therefore, nobiletin was applied to the soil on the day that the potatoes emerged. Potato root biomass and aboveground growth were significantly higher in nobiletin-treated plants than in the controls at 10 DAE, ultimately leading to a 35.86% increase in potato yield (Figure 5D and 5E; Supplemental Figure 9). Although the direct application of nobiletin was less effective than the direct application of *Pantoea* sp. MCC16, the findings confirmed the feasibility of this approach. Therefore, manipulating the timing and composition of root exudates can effectively enhance the colonization of beneficial bacteria and lead to more sustainable and efficient agricultural practices, reducing reliance on synthetic fertilizers and pesticides. By identifying and promoting the growth of indigenous beneficial bacteria, farmers can enhance plant health and resilience, leading to higher yields and reduced environmental impacts. The use of natural compounds such as nobiletin to stimulate beneficial bacterial growth could also represent a cost-effective and environmentally friendly strategy for improving crop performance.

In conclusion, this study revealed that potatoes mitigate CC obstacles by increasing root IAA content and AR numbers under vanillin stress in the soil. Potato roots counteract vanillin stress by secreting nobiletin, attracting the high-IAA producing bacterium *Pantoea* sp. MCC16, raising the levels of IAA in the rhizosphere, enhancing root IAA uptake, increasing AR numbers, and improving plant resilience, thereby alleviating CC challenges (Figure 6).

METHODS

CC field experiment and sample collection

The potato CC positioning test was initiated in 2011 at the potato experimental station (35°6′41″ N, 103°59′4″ E, 2276 m above sea level) in

Weiyuan County, Dingxi City, Gansu Province, China, with an annual average temperature of 6.8°C, an annual rainfall amount of 500 mm, and a frost-free period of 166 days. The soil type was black loam, which is loose, soft, and easy to cultivate. The physical and chemical properties of the soil in the cultivated layer (0–20 cm) of the abandoned land were as follows: total nitrogen 0.746 g kg^{-1}, total phosphorus 0.638 g kg^{-1}, alkaline nitrogen 42.02 mg kg^{-1}, available phosphorus 13.80 mg kg^{-1}, available potassium 184.99 mg kg^{-1}, organic matter 16.26 g kg^{-1}, and a pH of 8.46. The only variable in this test was the number of CC years. Each year, a new piece of land was introduced as a CC treatment for 1 year. By 2020, the plots had durations of CC ranging from 1 to 10 years. Owing to the short frost-free period in the area, potatoes are planted in early May and harvested in early October each year. The potato variety was Longshu no. 7 (pre-elite seed), which was provided by the Potato Research Institute of the Gansu Academy of Agricultural Sciences (Dingxi, China). Each CC period was represented by one plot, with an area of 48.6 m^2 (9 $\times$ 5.4 m), row spacing of 60 cm, plant spacing of 30 cm, and planting density of 5.56 $\times$ 10^4 plants ha^{-1}. Fertilization occurred on the day of potato planting and included 150 kg ha^{-1} nitrogen fertilizer (urea, 46% N; one-quarter of the nitrogen fertilizer was top-dressed during the budding stage in combination with soil cultivation), 120 kg ha^{-1} phosphate fertilizer (diammonium phosphate, 18% N and 46% P_2O_5), 75 kg ha^{-1} potassium fertilizer (52% K_2O), and 37.5 t ha^{-1} of organic fertilizer (fresh sheep manure). Weeding was performed manually, and 25% leucine was applied during the budding stage to control aphids. Metalaxyl manganese zinc (58%) was used to control late blight, with four sprays performed based on field conditions.

On the basis of previous research (Ma et al., 2023b), 7 years was selected as the representative period for potato CC obstacles. Therefore, the CC treatment in this study refers to 7 years of CC, whereas the NCC treatment refers to 1 year of CC. Previous studies (Ma et al., 2023b) also indicated that the autotoxicity effect of CC significantly inhibits AR numbers in potato seedlings during the mid-seedling stage. However, field experiment observations revealed no significant difference in AR numbers between CC and NCC during the late seedling stage. To further investigate the changes in AR number during the seedling stage, we collected CC and NCC potato plants from the field and analyzed plant morphology, root IAA content, root expression of genes related to IAA synthesis and response, soil vanillin content, and yield. Details of sample collection are provided in Supplementary Method 1.

Soil sterilization and microbial transfer experiments to identify soil microbial functions in CC

CC soil sterilization test

Four treatments were used: unsterilized CC soil, CCS, unsterilized NCC soil, and sterilized NCC soil. The test soil and seed potatoes (Longshu no. 7, pre-elite seed) were transported from Weiyuan Potato Experimental Station, Dingxi City, Gansu Province, China, to Sichuan Agricultural University (Chengdu, China) in late November 2021. For the sterilization treatment, we used two high-pressure steam sterilization events (121°C, 1.5 h) with an interval of 3 days (Zheng et al., 2017). After the second sterilization, sterilized soil extract was isolated and cultured on Luria–Bertani (LB) solid medium to ensure that no bacteria could be isolated from the soil. The specific test procedure was as follows. First, 30 pots were set up for each treatment (300 mm in upper diameter $\times$ 210 mm in height). Each pot was filled with 4 kg of soil and then planted with one grain of whole potato weighing approximately 50 g at a sowing depth of 10 cm. The seed potatoes were disinfected with 0.1% sodium hypochlorite solution for 20 min and then washed with sterile distilled water. When potato plants had emerged to approximately 1 cm, they were watered with 200 ml Hoagland nutrient solution once every 7 days. The application times of the Hoagland nutrient solution were increased appropriately according to the potato growth, and watering ceased after the tuber-formation stage. Throughout the potato growth process, the water supply was appropriate, pests and diseases were controlled, and weeding was performed manually. For details of sample collection, please see Supplementary Method 2.

Microbial flora transfer test

The rhizosphere soils of CC and NCC plants in the CC potato field test were used as the source of microbial flora, and potatoes were planted in the field as described in the first experiment. First, field cultivation was performed in early May 2022, and a new plot was used for the NCC treatment (with specific cultivation measures such as those in the first experiment). Before potato planting, 400 kg of CC topsoil was collected and transported to Sichuan Agricultural University for sterilization treatment (as described above), then used as the basic soil for the subsequent experiment. At 10, 20, and 30 DAE, 10 CC and 10 NCC plants were selected. Soil clods of approximately 20 cm around the stem base of the plants were loosened with a shovel. After the plants were gently pulled out, some of the soil was shaken off, but the roots remained wrapped in soil. The rhizosphere soil of different plants from the same treatment was then collected into the same aseptic bag with a sterile brush, mixed, and stored temporarily at 4°C.

To ensure maximum microbial activity in the rhizosphere soils, pot tests were performed on the day after rhizosphere soil collection (10, 20, and 30 DAE). Therefore, the experiment was divided into three periods with intervals of 10 days. The method for microflora transfer was based on Jin et al. (2023). The specific method was as follows. CC and NCC rhizosphere soils were transferred to sterile soil at a 3% (g) inoculation rate, with a total of 4 kg soil per pot (300-mm upper diameter $\times$ 210-mm height). Potatoes were disinfected with 0.1% sodium hypochlorite solution for 20 min before planting and then washed with sterile distilled water. One seed potato was planted in each pot at a planting depth of 10 cm. Fifteen pots were set up for each treatment, and the pots were placed in a light culture room for cultivation (12-h light, 12-h dark). When the potato plants had emerged to approximately 1 cm, 200 ml Hoagland nutrient solution was applied once every 7 days. The application times of the Hoagland nutrient solution were increased appropriately according to potato growth, and watering ceased after the tuber-formation stage. During the potato growth process, the water supply was appropriate, pests and diseases were controlled, and weeding was performed manually. Potato plant samples were collected from each treatment at 10, 20, and 30 DAE, and the effects of rhizosphere flora on potato AR number, root IAA content, rhizosphere soil IAA content, soil vanillin content, and yield were determined. Potato yield was measured in five pots.

Profiling the potato rhizosphere microbiome

16S rRNA absolute quantitative analysis of rhizosphere bacterial communities was performed at 10 and 20 DAE in CC (CC10, CC20) and NCC (NCC10, NCC20) soils to explore the structure of the rhizosphere soil microbial community during the period. For details of 16S rRNA sequencing, please see Supplementary Method 3.

Isolation of rhizosphere bacteria and screening of IAA-producing strains and their effects on potato growth

Rhizosphere bacteria were isolated and cultured at 10 (I), 20 (M), and 30 (F) DAE in the CC and NCC treatments. The specific steps were as follows. In June 2022, plants in CC and NCC soils were retrieved from the field, and rhizosphere soil was collected. Owing to the difference in sampling times, the standard continuous-dilution culture method was used to isolate culturable bacteria immediately after retrieval. Soil suspensions with different concentrations (*n* = 5) were plated on solid LB medium and cultured overnight at 37°C. After colony formation, labels were made successively according to colony morphology, and bacteria were picked out. Purified single colonies were obtained by plate marking on the corresponding LB medium, and the lines of single colonies were redrawn twice to ensure colony purity. Finally, the strains were inoculated into LB broth medium and stored at −80°C.

Salkowski's reagent method (Sarwar and Kremer, 1995) was used to examine the IAA production capacity of all isolated bacteria. Seven strains of bacteria with an IAA production capacity greater than 20 μg ml^{-1} were identified, followed by sequence alignment (Supplementary Table 2; Supplemental Figure 4C). According to the identifications, the MCC16 strain and the unique strain ASV7733 identified in CC20 rhizosphere soil were the same strain, and this strain also had the strongest IAA production capacity. Therefore, an irrigation test with MCC16 bacterial solution was designed to determine whether this strain could promote the growth of CC potatoes. The specific design of this experiment is described in Supplementary Method 4.

Pot experiment to determine the effect of CC root exudates on potato growth and *Pantoea* sp. MCC16

Root exudates of CC and NCC potato were collected at 10 and 20 DAE and used to irrigate CC potato. The specific procedure is described in Supplementary Method 5.

Nontargeted metabolomics analysis and differential metabolite screening of root exudates

Nontargeted metabolomics analysis of root exudates from CC10, CC20, NCC10, and NCC20 was performed to identify which metabolites had major effects. Root exudates were collected, then freeze-dried in an LGJ-18S vacuum freeze dryer (Bonnin Instrument Technology, Wuhan, China) and stored at −80°C. The nontargeted metabolomics procedure is described in Supplementary Method 6. The purpose of the differential metabolite screening was to screen for substances that increased significantly in the root exudates of CC20 potato. Significance analysis (TOP50), random forest analysis (TOP40), KEGG pathway enrichment analysis (TOP10), and PCA (TOP40) were used to screen for substances that increased significantly in the root exudates of CC20 potato. The screened metabolic substances were significantly more abundant in CC20 than in CC10 and NCC20.

Effects of differential metabolites on chemotaxis and biofilm formation by *Pantoea* sp. MCC16

The procedures used to detect chemotaxis and biofilm formation *in vivo* are described in Supplementary Method 7. Supplementary Method 8 describes the approach used to verify whether the target metabolite (nobiletin) increased AR numbers in CC potatoes through soil microorganisms. Chemotaxis and biofilm formation were then verified in the soil. First, sterilized CC soil was placed in pots (10 cm in upper diameter × 10 cm in height). Ten pots received injections of nobiletin, and 10 pots received injections of sterile water as a control. Each pot contained 400 g of soil, and 1 ml of *Pantoea* sp. MCC16 bacterial solution (1.0×10^8 colony-forming units [CFU] ml^{-1}) was injected vertically into the soil with a 1-ml syringe at four locations 4 cm from the center of the pot. A syringe was also used to vertically inject 10 ml of 20 μM nobiletin solution or sterile water as the control, and the pots were then placed at 37°C for constant temperature culture, during which the same amount of nobiletin solution was added every 3 days. The central soil column of the pots was collected with a hole punch (1-cm diameter) on the 10th day. Bacteria were isolated and cultured by the standard continuous-dilution culture method, and the number of *Pantoea* sp. MCC16 was counted.

Effects of exogenous application of *Pantoea* sp. MCC16 and nobiletin on the growth of CC potato

Using CC soil as the background, the following four treatments were established: sterile water, nobiletin solution (20 μM), *Pantoea* sp. MCC16 bacterial solution (1.0×10^8 CFU ml^{-1}), and nobiletin solution combined with *Pantoea* sp. MCC16 bacterial solution. Twenty pots were set up for each treatment, and each pot (300-mm upper diameter × 210-mm height) was filled with 4 kg of CC soil and planted with one seed potato. In the nobiletin treatment, potatoes were watered at 3, 6, and 9 DAE. In the *Pantoea* sp. MCC16 bacterial solution treatment, pots were irrigated once with 350 ml on the day of potato emergence. At 10 and 20 DAE, potato plants were collected to determine plant morphology, AR numbers, root IAA content, rhizosphere soil IAA content, and soil vanillin content. The yield of 10 plants per treatment was determined at harvest.

Statistical analyses

Community alpha diversity indices (i.e., operational taxonomic unit [OTU] number, Shannon index, and abundance-based coverage estimator [ACE] index) were calculated using the vegan package in R (Oksanen et al., 2019). Beta diversity was analyzed and visualized using Bray–Curtis dissimilarity-based principal coordinate analysis. To test the effects of CC on community dissimilarities (i.e., whether there were differences in beta diversity), a PERMANOVA test was performed using the Adonis function in the vegan 2.6-4 package (Helsinki University, Finland) for 9999 permutations (Oksanen et al., 2019). Functional inference was performed using PICRUSt2.0 against the KEGG pathway database (Douglas et al., 2020). The *p* values were corrected for multiple comparisons using the Benjamini–Hochberg false discovery rate method (Lydersen, 2024). To identify the exudate compounds that made the greatest contributions to the classification, the random forest method and default parameters of the algorithm R implementation were used (R package RandomForest, ntree = 1000). Correlations were examined using Pearson correlation analysis. Enrichment analysis of metabolic pathways was performed using the online platform MetaboAnalyst (http://www.metaboanalyst.ca/faces/home.xhtml). A phylogenetic tree of bacterial rhizosphere isolates was constructed using MEGA 6 (Temple University, Philadelphia, PA, USA). Phylogenetic trees were annotated and visualized using Adobe Illustrator software (Adobe, San Jose, CA, USA).

All data were tested for normality (Shapiro–Wilk test) and homogeneity of variance (Levene's test). To analyze microbial abundance data, we applied logarithmic transformation. Comparisons between two groups were performed using Welch's *t*-test, whereas comparisons among three or more groups were performed by one-way ANOVA followed by Tukey's honestly significant difference (HSD) test. Statistical significance was determined at $p < 0.05$. Figure 3B was plotted using the Bioinformatics online platform (https://www.bioinformatics.com.cn/). Figures 4D and 5A were plotted using the Chiplot online platform (https://www.chiplot.online/). Figure 6 was plotted using the Figdraw online platform (https://www.figdraw.com/static/index.html#/). Supplemental Figure 1 was prepared in PowerPoint (Microsoft Corporation, Redmond, WA, USA). Other figures were plotted using GraphPad Prism 9 (GraphPad Software, La Jolla, CA, USA).

DATA AND CODE AVAILABILITY

Raw 16S rRNA sequencing data were deposited in the Genome Sequence Archive at the China National Genomics Data Center under accession number CRA015542 (https://ngdc.cncb.ac.cn/gsa/), and metabolite profiling data were deposited in the EMBL-EBI metabolomics repository under the identifier MTBLS12358 (https://www.ebi.ac.uk/metabolights/search).

FUNDING

This research was supported by the Natural Science Foundation of Sichuan Province (grant number 2022NSFSC0014), the Tackling Key Problems and Supporting Projects of Breeding in Sichuan Province (grant numbers 2021YFYZ0019 and 2021YFYZ0005), the Sichuan Potato Innovation Team (grant number sccxtd–2023–09), and the National Natural Science Foundation of China (grant number 32060443).

ACKNOWLEDGMENTS

We extend our gratitude to Juanjuan Liu, Wensen Huang, Yang Li, and Xinyu Zheng for their invaluable assistance. No conflict of interest is declared.

AUTHOR CONTRIBUTIONS

H.M., Z.R., and A.L. conceived and designed the study. H.M., X.F., R.L., X. Shi, C.W., K.Z., and S.Z. performed the experiments and analyzed the

data. H.M., X. Sun, J.L., and H.L. wrote the manuscript. All authors edited the manuscript and approved the final version.

SUPPLEMENTAL INFORMATION

Supplemental information is available at *Plant Communications Online*.

Received: May 7, 2024
Revised: June 13, 2024
Accepted: May 8, 2025
Published: May 12, 2025

REFERENCES

Alabouvette, C., Olivain, C., Migheli, Q., and Steinberg, C. (2009). Microbiological control of soil-borne phytopathogenic fungi with special emphasis on wilt-inducing Fusarium oxysporum. New Phytol. **184**:529–544.

Angulo, V., Beriot, N., Garcia-Hernandez, E., Li, E., Masteling, R., and Lau, J.A. (2022). Plant–microbe eco-evolutionary dynamics in a changing world. New Phytol. **234**:1919–1928.

Bakker, P.A.H.M., Pieterse, C.M.J., De Jonge, R., and Berendsen, R.L. (2018). The soil-borne legacy. Cell **172**:1178–1180.

Berendsen, R.L., Pieterse, C.M.J., and Bakker, P.A.H.M. (2012). The rhizosphere microbiome and plant health. Trends Plant Sci. **17**:478–486.

Berlanga-Clavero, M.V., Molina-Santiago, C., de Vicente, A., and Romero, D. (2020). More than words: the chemistry behind the interactions in the plant holobiont. Environ. Microbiol. **22**:4532–4544.

Canarini, A., Schmidt, H., Fuchslueger, L., Martin, V., Herbold, C.W., Zezula, D., Gündler, P., Hasibeder, R., Jecmenica, M., Bahn, M., and Richter, A. (2021). Ecological memory of recurrent drought modifies soil processes via changes in soil microbial community. Nat. Commun. **12**:5308.

Chen, Y., Peng, Y., Dai, C.-C., and Ju, Q. (2011). Biodegradation of 4-hydroxybenzoic acid by *Phomopsis liquidambari*. Appl. Soil Ecol. **51**:102–110.

Chen, Y., Yang, L., Zhang, L., Li, J., Zheng, Y., Yang, W., Deng, L., Gao, Q., Mi, Q., Li, X., et al. (2023). Autotoxins in continuous tobacco cropping soils and their management. Front. Plant Sci. **14**:1106033.

Chung, J.h., Song, G.C., and Ryu, C.-M. (2016). Sweet scents from good bacteria: Case studies on bacterial volatile compounds for plant growth and immunity. Plant Mol. Biol. **90**:677–687.

Cordovez, V., Rotoni, C., Dini-Andreote, F., Oyserman, B., Carrión, V. J., and Raaijmakers, J.M. (2021). Successive plant growth amplifies genotype-specific assembly of the tomato rhizosphere microbiome. Sci. Total Environ. **772**:144825.

Davis, A.G., Huggins, D.R., and Reganold, J.P. (2023). Linking soil health and ecological resilience to achieve agricultural sustainability. Front. Ecol. Environ. **21**:131–139.

Douglas, G.M., Maffei, V.J., Zaneveld, J.R., Yurgel, S.N., Brown, J.R., Taylor, C.M., Huttenhower, C., and Langille, M.G.I. (2020). PICRUSt2 for prediction of metagenome functions. Nat. Biotechnol. **38**:685–688.

Etesami, H., and Glick, B.R. (2024). Bacterial indole-3-acetic acid: A key regulator for plant growth, plant-microbe interactions, and agricultural adaptive resilience. Microbiol. Res. **281**:127602.

Gallage, N.J., and Møller, B.L. (2015). Vanillin-bioconversion and bioengineering of the most popular plant flavor and its de novo biosynthesis in the vanilla orchid. Mol. Plant **8**:40–57.

Gong, X., Feng, Y., Dang, K., Jiang, Y., Qi, H., and Feng, B. (2023). Linkages of microbial community structure and root exudates: Evidence from microbial nitrogen limitation in soils of crop families. Sci. Total Environ. **881**:163536.

Guo, K., He, X., Yan, Z., Li, X., Ren, X., Pan, L., and Qin, B. (2016). Allelochemicals from the rhizosphere soil of cultivated *Astragalus hoantchy*. J. Agr. Food Chem. **64**:3345–3352.

He, D., Singh, S.K., Peng, L., Kaushal, R., Vílchez, J.I., Shao, C., Wu, X., Zheng, S., Morcillo, R.J.L., Paré, P.W., and Zhang, H. (2022). Flavonoid-attracted Aeromonas sp. from the *Arabidopsis* root microbiome enhances plant dehydration resistance. ISME J. **16**:2622–2632.

Hernández-Forte, I., Pérez-Pérez, R., Taulé-Gregorio, C.B., Fabiano-González, E., Battistoni-Urrutia, F., and Nápoles-García, M.C. (2022). New bacteria genera associated with rice *(Oryza sativa L.*) in Cuba promote the crop growth. Agron. Mesoam. **33**:47223.

Huang, Y., ZHANG, E., ZHANG, X., WANG, H., Wang, Q., LIU, Q., and SHI, Y. (2017). Autotoxicity of water extracts of the root and bulb of *Lilium davidii var. unicolor Salisb*. Acta Prataculturae Sinica **26**:93–103.

Jiang, M., Delgado-Baquerizo, M., Yuan, M.M., Ding, J., Yergeau, E., Zhou, J., Crowther, T.W., and Liang, Y. (2023). Home-based microbial solution to boost crop growth in low-fertility soil. New Phytol. **239**:752–765.

Jin, X., Rahman, M. K. u, Ma, C., Zheng, X., Wu, F., and Zhou, X. (2023). Silicon modification improves biochar's ability to mitigate cadmium toxicity in tomato by enhancing root colonization of plant-beneficial bacteria. Ecotox Environ Safe **249**:114407.

Kawai, M., Hirano, T., Higa, S., Arimitsu, J., Maruta, M., Kuwahara, Y., Ohkawara, T., Hagihara, K., Yamadori, T., Shima, Y., et al. (2007). Flavonoids and related compounds as anti-allergic substances. Allergol. Int. **56**:113–123.

Kumar, P., Chauhan, V., Dang, A.S., Kumar, A., and Suneja, P. (2022). Draft genome sequence of *Pantoea agglomerans* CPHN2, a potential plant-growth-promoting endophyte. Microbiol. Resour. Ann. **11**: e00192-22.

Kwak, M.-J., Kong, H.G., Choi, K., Kwon, S.-K., Song, J.Y., Lee, J., Lee, P.A., Choi, S.Y., Seo, M., Lee, H.J., et al. (2018). Rhizosphere microbiome structure alters to enable wilt resistance in tomato. Nat. Biotechnol. **36**:1100–1109.

Li, X., Jousset, A., de Boer, W., Carrión, V.J., Zhang, T., Wang, X., and Kuramae, E.E. (2019). Legacy of land use history determines reprogramming of plant physiology by soil microbiome. ISME J. **13**:738–751.

Li, H., Zhang, Y., Zhang, Q., Ahmad, N., Liu, P., Wang, R., Li, J., and Wang, X. (2021). Converting continuous cropping to rotation including subsoiling improves crop yield and prevents soil water deficit: A 12-yr in-situ study in the loess plateau, China. Agr. Water Manage. **256**:107062.

Liu, Y., Wang, H., Qian, X., Gu, J., Chen, W., Shen, X., Tao, S., Jiao, S., and Wei, G. (2023). Metagenomics insights into responses of rhizobacteria and their alleviation role in licorice allelopathy. Microbiome **11**:109.

Lydersen, S. (2024). Adjustment of p values for multiple hypotheses: why, when and how. Ann. Rheum. Dis. **83**:1254–1255. https://doi.org/10.1136/ard-2024-225537.

Lyu, J., Jin, N., Meng, X., Jin, L., Wang, S., Xiao, X., Liu, Z., Tang, Z., and Yu, J. (2022). Exogenous silicon alleviates the adverse effects of cinnamic acid-induced autotoxicity stress on cucumber seedling growth. Front. Plant Sci. **13**:968514.

Ma, A., Lu, X., Gray, C., Raybould, A., Tamaddoni-Nezhad, A., Woodward, G., and Bohan, D.A. (2019). Ecological networks reveal resilience of agro-ecosystems to changes in farming management. Nat. Ecol. Evol. **3**:260–264.

Ma, Z., Guan, Z., Liu, Q., Hu, Y., Liu, L., Wang, B., Huang, L., Li, H., Yang, Y., Han, M., et al. (2023a). Obstacles in continuous cropping: Mechanisms and control measures. Adv. Agron. **179**:205–256.

Ma, H., Li, J., Luo, A., Lv, H., Ren, Z., Yang, H., Fang, X., Shahzad, M.A., Qu, H., Zhang, K., et al. (2023b). Vanillin, a newly discovered autotoxic substance in long-term potato continuous cropping soil, inhibits plant growth by decreasing the root auxin content and reducing adventitious root numbers. J. Agric. Food Chem. **71**:16993–17004.

Maeda, H., and Dudareva, N. (2012). The Shikimate Pathway and Aromatic Amino Acid Biosynthesis in Plants. Annu. Rev. Plant Biol. **63**:73–105.

Mandal, S.M., Chakraborty, D., and Dey, S. (2010). Phenolic acids act as signaling molecules in plant-microbe symbioses. Plant Signal. Behav. **5**:359–368.

Martinez, V., Mestre, T.C., Rubio, F., Girones-Vilaplana, A., Moreno, D. A., Mittler, R., and Rivero, R.M. (2016). Accumulation of flavonols over hydroxycinnamic acids favors oxidative damage protection under abiotic stress. Front. Plant Sci. **7**:838.

Ni, X., Jin, C., Liu, A., Chen, Y., and Hu, Y. (2021). Physiological and transcriptomic analyses to reveal underlying phenolic acid action in consecutive monoculture problem of *Polygonatum odoratum*. BMC Plant Biol. **21**:362.

Oksanen, J., Blanchet, F.G., Friendly, M., Kindt, R., Legendre, P., McGlinn, D., Minchin, P.R., O'hara, R., Simpson, G.L., and Solymos, P. (2019). Vegan: Community Ecology Package (R Package Version 2).

Orozco-Mosqueda, M.d.C., and Santoyo, G. (2021). Plant-microbial endophytes interactions: Scrutinizing their beneficial mechanisms from genomic explorations. Curr. Plant Biol. **25**:100189.

Pantoja-Guerra, M., Burkett-Cadena, M., Cadena, J., Dunlap, C.A., and Ramírez, C.A. (2023). *Lysinibacillus* spp.: an IAA-producing endospore forming-bacteria that promotes plant growth. Antonie Leeuwenhoek **116**:615–630.

Pérez-Jaramillo, J.E., Carrión, V.J., Bosse, M., Ferrão, L.F.V., de Hollander, M., Garcia, A.A.F., Ramírez, C.A., Mendes, R., and Raaijmakers, J.M. (2017). Linking rhizosphere microbiome composition of wild and domesticated *Phaseolus vulgaris* to genotypic and root phenotypic traits. ISME J. **11**:2244–2257.

Raaijmakers, J.M., and Mazzola, M. (2016). Soil immune responses. Science **352**:1392–1393.

Revillini, D., David, A.S., Reyes, A.L., Knecht, L.D., Vigo, C., Allen, P., Searcy, C.A., and Afkhami, M.E. (2023). Allelopathy-selected microbiomes mitigate chemical inhibition of plant performance. New Phytol. **240**:2007–2019.

Rodriguez, P.A., Rothballer, M., Chowdhury, S.P., Nussbaumer, T., Gutjahr, C., and Falter-Braun, P. (2019). Systems biology of plant-microbiome interactions. Mol. Plant **12**:804–821.

Rolfe, S.A., Griffiths, J., and Ton, J. (2019). Crying out for help with root exudates: Adaptive mechanisms by which stressed plants assemble health-promoting soil microbiomes. Curr. Opin. Microbiol. **49**:73–82.

Santoyo, G. (2022). How plants recruit their microbiome? New insights into beneficial interactions. J. Adv. Res. **40**:45–58.

Sarwar, M., and Kremer, R.j. (1995). Determination of bacterially derived auxins using a microplate method. Lett. Appl. Microbiol. **20**:282–285.

Shi, S., Nuccio, E., Herman, D.J., Rijkers, R., Estera, K., Li, J., da Rocha, U.N., He, Z., Pett-Ridge, J., Brodie, E.L., et al. (2015). Successional trajectories of rhizosphere bacterial communities over consecutive seasons. mBio **6**:e00746. https://doi.org/10.1128/mbio.00746-15.

Singh, G., Agrawal, H., and Bednarek, P. (2023). Specialized metabolites as versatile tools in shaping plant–microbe associations. Mol. Plant **16**:122–144.

Song, Y., Wilson, A.J., Zhang, X.-C., Thoms, D., Sohrabi, R., Song, S., Geissmann, Q., Liu, Y., Walgren, L., He, S.Y., and Haney, C.H. (2021). FERONIA restricts *Pseudomonas* in the rhizosphere microbiome via regulation of reactive oxygen species. Nat. Plants **7**:644–654.

Stringlis, I.A., Yu, K., Feussner, K., de Jonge, R., Van Bentum, S., Van Verk, M.C., Berendsen, R.L., Bakker, P.A.H.M., Feussner, I., and Pieterse, C.M.J. (2018). MYB72-dependent coumarin exudation shapes root microbiome assembly to promote plant health. Proc. Natl. Acad. Sci. USA **115**:E5213–E5222.

Sui, J., Yang, J., Li, C., Zhang, L., and Hua, X. (2023). Effects of a microbial restoration substrate on plant growth and rhizosphere microbial community in a continuous cropping poplar. Microorganisms **11**:486.

Sujkowska-Rybkowska, M., Rusaczonek, A., Kasowska, D., Gediga, K., Banasiewicz, J., Stępkowski, T., and Bernacki, M.J. (2022). Potential of rhizobia *Nodulating Anthyllis vulneraria* L. from ultramafic soil as plant growth promoting bacteria alleviating nickel stress in *Arabidopsis thaliana* L. Int. J. Mol. Sci. **23**:11538.

Sun, Q., Li, X., Sun, L., Sun, M., Xu, H., and Zhou, X. (2024). Plant hormones and phenolic acids response to UV-B stress in *Rhododendron chrysanthum pall*. Biol. Direct **19**:40.

Tan, Y., Cui, Y., Li, H., Kuang, A., Li, X., Wei, Y., and Ji, X. (2017). Diversity and composition of rhizospheric soil and root endogenous bacteria in *Panax notoginseng* during continuous cropping practices. J Basic Microb **57**:337–344.

Thiergart, T., Zgadzaj, R., Bozsóki, Z., Garrido-Oter, R., Radutoiu, S., and Schulze-Lefert, P. (2019). *Lotus japonicus* symbiosis genes impact microbial interactions between symbionts and multikingdom commensal communities. mBio **10**:e01833-19. https://doi.org/10.1128/mbio.01833-19.

Vannier, N., Mony, C., Bittebiere, A.-K., Michon-Coudouel, S., Biget, M., and Vandenkoornhuyse, P. (2018). A microorganisms' journey between plant generations. Microbiome **6**:79.

Venturi, V., and Keel, C. (2016). Signaling in the rhizosphere. Trends Plant Sci. **21**:187–198.

Wang, X., Wei, Z., Yang, K., Wang, J., Jousset, A., Xu, Y., Shen, Q., and Friman, V.-P. (2019). Phage combination therapies for bacterial wilt disease in tomato. Nat. Biotechnol. **37**:1513–1520.

Wang, C., Liu, Z., Wang, Z., Pang, W., Zhang, L., Wen, Z., Zhao, Y., Sun, J., Wang, Z.Y., and Yang, C. (2022). Effects of autotoxicity and allelopathy on seed germination and seedling growth in *Medicago truncatula*. Front. Plant Sci. **13**:908426.

Wang, K., Lu, Q., Dou, Z., Chi, Z., Cui, D., Ma, J., Wang, G., Kuang, J., Wang, N., and Zuo, Y. (2024a). A review of research progress on continuous cropping obstacles. Front. Agri. Sci. Eng. **11**:253.

Wang, X., Zhang, J., Lu, X., Bai, Y., and Wang, G. (2024b). Two diversities meet in the rhizosphere: root specialized metabolites and microbiome. J. Genet. Genomics **51**:467–478.

Wen, T., Yuan, J., He, X., Lin, Y., Huang, Q., and Shen, Q. (2020). Enrichment of beneficial cucumber rhizosphere microbes mediated by organic acid secretion. Hortic. Res. **7**:154.

Wen, T., Xie, P., Liu, H., Liu, T., Zhao, M., Yang, S., Niu, G., Hale, L., Singh, B.K., Kowalchuk, G.A., et al. (2023). Tapping the rhizosphere metabolites for the prebiotic control of soil-borne bacterial wilt disease. Nat. Commun. **14**:4497.

Wilkinson, D.M. (1997). The role of seed dispersal in the evolution of mycorrhizae. Oikos **78**:394–396.

Wilkinson, D.M., and Sherratt, T.N. (2001). Horizontally acquired mutualisms, an unsolved problem in ecology? Oikos **92**:377–384.

Xiang, W., Chen, J., Zhang, F., Huang, R., and Li, L. (2022). Autotoxicity in *Panax notoginseng* of root exudatesand their allelochemicals. Front. Plant Sci. **13**:1020626.

Xuan, P., Ma, H., Deng, X., Li, Y., Tian, J., Li, J., Ma, E., Xu, Z., Xiao, D., Bezemer, T.M., et al. (2024). Microbiome-mediated alleviation of tobacco replant problem via autotoxin degradation after long-term continuous cropping. iMeta **3**:e189.

Yang, Z., Cao, Y., Shi, Y., Qin, F., Jiang, C., and Yang, S. (2023). Genetic and molecular exploration of maize environmental stress resilience: Toward sustainable agriculture. Mol. Plant **16**:1496–1517.

Yu, X.-Q., Yan, X., Zhang, M.-Y., Zhang, L.-Q., and He, Y.-X. (2020). Flavonoids repress the production of antifungal 2,4-DAPG but potentially facilitate root colonization of the rhizobacterium *Pseudomonas fluorescens*. Environ. Microbiol. **22**:5073–5089.

Yuan, J., Zhao, J., Wen, T., Zhao, M., Li, R., Goossens, P., Huang, Q., Bai, Y., Vivanco, J.M., Kowalchuk, G.A., et al. (2018). Root exudates drive the soil-borne legacy of aboveground pathogen infection. Microbiome **6**:156.

Zhang, W., and Chen, Y. (2014). Analysis of the bacterial communities in continuous cotton fields of Xinjiang province. Acta Ecol. Sin. **34**:4682–4689.

Zhang, Z.Y., Pan, L.P., and Li, H.H. (2010). Isolation, identification and characterization of soil microbes which degrade phenolic allelochemicals. J. Appl. Microbiol. **108**:1839–1849.

Zhang, J., Liu, Y.-X., Zhang, N., Hu, B., Jin, T., Xu, H., Qin, Y., Yan, P., Zhang, X., Guo, X., et al. (2019). NRT1.1B is associated with root microbiota composition and nitrogen use in field-grown rice. Nat. Biotechnol. **37**:676–684.

Zhao, J., Zhang, D., Yang, Y., Pan, Y., Zhao, D., Zhu, J., Zhang, L., and Yang, Z. (2020). Dissecting the effect of continuous cropping of potato on soil bacterial communities as revealed by high-throughput sequencing. PLoS One **15**:e0233356.

Zheng, J., Chen, H., Li, J., Nie, M., and Fang, C. (2017). The effects of different soil sterilization treatments on soil microbial activity. J. Fudan Univ. (Natural Science) **56**:11.

Zheng, Y., Cao, X., Zhou, Y., Ma, S., Wang, Y., Li, Z., Zhao, D., Yang, Y., Zhang, H., Meng, C., et al. (2024). Purines enrich root-associated *Pseudomonas* and improve wild soybean growth under salt stress. Nat. Commun. **15**:3520.

Plant Communications, Volume *6*

Supplemental information

Self-alleviation of continuous-cropping obstacles in potato via root-exudate-driven recruitment of growth-promoting bacteria

Haiyan Ma, Zhitong Ren, Aihua Luo, Xiaoting Fang, Ruilin Liu, Chao Wu, Xinxin Shi, Junji Li, Heping Lv, Xiaohua Sun, Kaiqin Zhang, and Shunlin Zheng

Self-alleviation of continuous cropping obstacles in potato via root exudate-driven recruitment of growth-promoting bacteria

Haiyan Ma[1,2,5,6], Zhitong Ren[1,2,6], Aihua Luo[3,6], Xiaoting Fang[1,2], Ruilin Liu[1,2], Chao Wu[1,2], Xinxin Shi[1,2], Junji Li[1,2], Heping Lv[3], Xiaohua Sun[3], Kaiqin Zhang[1,2], and Shunlin Zheng[1,2,4*]

[1] State Key Laboratory of Crop Gene Exploration and Utilization in Southwest China, College of Agronomy, Sichuan Agricultural University, Chengdu 611130, P.R. China

[2] Crop Ecophysiology and Cultivation Key Laboratory of Sichuan Province, Chengdu 611130, China

[3] Potato Research Institute of Gansu Academy of Agricultural Sciences, Lanzhou, 730070, China

[4] Key Laboratory of Tuber Crop Genetics and Breeding, Ministry of Agriculture, Chengdu Joyson Agricultural Technology Co., Ltd, Xindu 610500, China

[5] Yibin Academy of Agricultural Sciences, Yibin, 644699, China

[6] Co-first author

* **Correspondence: Shunlin Zheng (zhengshunlin123@163.com).**

Supplemental information

Supplementary Methods

Supplementary Method 1. Sample collection from continuous cropping field

For plants where the morphological indices were measured, sample preperation began with repeated shaking to remove most of the soil, followed by the collection of rhizosphere soil. Three rhizosphere soil samples were combined to form a single composite sample for the determination of the vanillin content. After rhizosphere soil collection, the roots were rinsed thoroughly with deionized water, air-dried, and measurements such as the plant height, stem diameter, and number of adventitious roots were recorded. The plant organs were then separated and initially heat-killed for 30 min at 105°C before being dried at 80°C until reaching a constant weight to determine the dry matter content. For the assessment of root IAA content and related gene expression, plant roots were washed with deionized water post-low temperature treatment and a 2-cm segment of the root tip was excised, promptly immersed in liquid nitrogen for freezing for 10 min, and then stored at −80°C. The IAA content in the roots was quantified using a plant growth hormone (IAA) ELISA kit (Jiangsu Meimian Industrial Co., Ltd., China). Total RNA extraction from the roots was conducted using the Trizol method to ensure adequate concentration and purity, and the extracted RNA was then reverse transcribed into cDNA using the HiScript III 1st Strand cDNA Synthesis Kit (Nanjing Nuoweizan Biotechnology Co., LTD., China). Gene expression analysis was conducted via qPCR using AceQ qPCR SYBR Green Master Mix (Nanjing Nuoweizan Biotechnology Co., LTD., China) on a QuantStudio 6 Flex instrument (Thermo Fisher, USA), utilizing the QuantStudio™ Real-Time PCR System software (Thermo Fisher, USA). The expression levels of target genes were calculated according to the $2^{-\Delta\Delta Ct}$ method, with StEF-1α serving as the internal reference gene. The primer sequences for these genes are listed in Supplementary Table 1. After harvesting, 20 potato plants from each treatment group were selected to assess the yield, which was then scaled up to calculate the yield per hectare. On the day of potato planting, a five-point sampling strategy was employed to clear away the soil surface stubble. Topsoil samples from both the continuous cropping (CC) and noncontinuous cropping (NCC) treatments were collected immediately following potato emergence and considered the day 0 soil samples. Subsequently, the soil vanillin content was evaluated in conjunction with rhizosphere soil samples collected at 10, 20, and 30 days after emergence (DAE). The extraction of vanillin from the soil was performed following the method outlined by Li et al. (2010). Phenolic acid quantification was conducted using a Shimadzu LC-20A liquid chromatograph (Shimadzu Corporation, Kyoto, Japan) equipped with an Agilent C18 column (dimensions: 4.6 mm × 250 mm, particle size: 5 μm). The analysis involved a flow rate of 1 mL min^{-1}, a column temperature of 25°C, and a UV detection wavelength of 280 nm, as described by Bai et

al. (2019).

Supplementary Method 2. Sample collection from the soil sterilization pot experiment

Plant and soil samples were systematically collected at 10, 20, and 30 days after emergence (DAE) to quantify various parameters, including the number of adventitious roots (ARs), the root IAA content, the expression of root-related IAA genes, the IAA content in the rhizosphere soil, the vanillin content in the rhizosphere soil, and the potato yield. At each sampling point, six plants were randomly selected for the assessments, with half (three plants) used for the measurement of physiological indices and the remaining three plants utilized to count the number of ARs. The procedure employed to collect potted plant samples was as follows: the selected plants were carefully removed from their pots and the bulk soil was gently shaken off. The rhizosphere soil was then meticulously collected using a sterile brush and deposited into a sterile sampling bag for the determination of the IAA and vanillin concentrations in the rhizosphere soil. These samples were temporarily stored at 4°C. Soil samples obtained from plants under the same treatment conditions were homogenized. After soil sample collection, the roots were thoroughly washed with deionized water and air-dried. Next, a 2-cm section of the root tip was excised from each individual root and placed in a cryovial, rapidly frozen in liquid nitrogen, and subsequently transferred to a −80°C freezer for storage, awaiting assays for root IAA content using an IAA ELISA kit (Jiangsu Meimian Industrial Co., Ltd., China) and gene expression analyses. Finally, the number of ARs was obtained, and 10 pots from each treatment were selected to determine the potato yield.

Supplementary Method 3. 16S rRNA sequencing of potato rhizosphere microbiome

Total genomic DNA was extracted from the soil samples using the FastDNA® SPIN Kit (MP Biomedicals, Santa Ana, CA, USA) according to the manufacturer's instructions. The quality, concentration, and purity of the extracted genomic DNA were validated using agarose gel electrophoresis, and measurements were conducted using both the Nanodrop 2000 and Qubit3.0 spectrophotometers. An internal standard mix containing gene fragments of known copy numbers (10^3, 10^4, 10^5, and 10^6 copies) was introduced into the sample DNA pools. These internal standard sequences contained conserved regions similar to those found in naturally occurring 16S rRNA genes and artificial variable regions, as previously described in Tkacz et al. (2018) and Jiang et al. (2019). To amplify the targeted V4–V5 hypervariable region of the 16S rRNA gene, the primers 515F (sequence: 5'-GTGCCAGCMGCCGCGG-3') and 907R (sequence: 5'-CCGTCAATTCMTTTRAGTTT-3') were utilized. The amplified products subsequently underwent sequencing using the Illumina NovaSeq 6000 platform (performed by

Genesky Biotechnologies Inc., located in Shanghai, China, zip code: 201315). To ensure that high-quality sequencing data were obtained, the DADA2 plugin within the QIIME2 software suite was used to conduct rigorous quality control measures, including filtering, denoising, merging, and chimera removal. This process generated feature tables and representative sequences (as documented by Callahan et al., 2016; Bolyen et al., 2019). Taxonomic assignments of ASV representative sequences were performed using a pre-trained Naive Bayes classifier trained on the RDP Ribosomal Database Project (RDP) database version 11.5. During the data processing, the internal standard sequences were recognized, and their read counts were tabulated; these sequences were later excluded from further analyses. Ultimately, each sample produced a standard curve based on its internal standard sequence reads, allowing sequences to be attributed to their corresponding samples based on each sample's unique barcode and thus enabling absolute quantification.

Supplementary Method 4. Effects of *Pantoea* sp. MCC16 bacterial solution watering test on potato growth

In this experiment, sterilized continuous cropping soil served as the base substrate and a total of 30 pots were prepared. Among them, 15 pots were inoculated with *Pantoea* sp. MCC16 bacterial solution, while the other 15 pots were treated with sterile water as controls. Each pot had dimensions of an upper diameter of 300 mm and a height of 210 mm and contained 4 kg of soil. Before planting, the potatoes were first disinfected in a 0.1% sodium hypochlorite solution for 20 min and then rinsed thoroughly with sterile distilled water. A single seed potato was planted in each pot at a planting depth of 10 cm. On the day of potato emergence, each pot received irrigation with either 350 mL of OD_{600}=1 bacterial solution (equivalent to 1.0×10^{8} CFU mL^{-1}) or the same volume of sterile water to ensure that the final soil concentration of the bacterial solution was maintained at 1.0×10^{8} CFU mL^{-1} per gram of dry soil in the inoculated pots. Plant samples were collected at three distinct time points at 10, 20, and 30 DAE. At these stages, several parameters were measured, including the number of adventitious roots, the root IAA content, the rhizosphere soil IAA content, the soil vanillin content, and the potato yield.

Supplementary Method 5. Effects of root exudates on potato plant growth

At 10 and 20 DAE, three plants from each continuous cropping and noncontinuous cropping treatment were carefully excavated to minimize root damage, ensuring that the roots remained enveloped in soil. These plants were swiftly transported back to the laboratory at a low temperature. Most of the adhering soil was delicately shaken off, and the roots were then rinsed with sterile distilled water. After rinsing, roots from plants

undergoing identical treatments were placed in a common beaker wrapped in tin foil to prevent light exposure. The beaker was then filled with sterile ultra-pure water containing 0.5 mM calcium chloride, and the roots were allowed to soak in this solution while being continuously aerated under dark conditions for a 12-h incubation period. Following incubation, the collected filtrate was filtered through a 0.22-μm filter membrane (Merck, Darmstadt, Germany). The plant roots were dried, and their fresh weight was recorded. The solution concentration was adjusted according to the fresh weight of the roots to standardize the results. The filtrate was then stored at −80°C following the methodology described by Williams et al.(2021). For the separate experiment involving the application of root exudates to potato plants, 10 pots were prepared for each root secretion filtrate treatment. Each pot was filled with 5 kg of natural soil collected from the CC environment and sowed with a single seed potato. On the day of potato emergence, 100 mL of root exudates was applied, followed by regular irrigation with 20 mL root exudates every 2 days until the 10th day post-emergence, following the methodologies of Valentinuzzi et al. (2015) and Zhou et al. (2023). The number of adventitious roots (ARs) was counted at 10 and 20 DAE, and the rhizosphere soil was collected to determine the population density of *Pantoea* sp. MCC16.

Supplementary Method 6. Nontargeted metabolomics analysis procedure

First, 100 mg of freeze-dried root exudates was dissolved in 500 μL of 80% methanol solution (Thermo Fisher, USA), vortexed for 30 s, placed on ice for 5 min, and then centrifuged at 15000 g and 4°C for 20 min (D3024R, Scilogex, USA). Following centrifugation,100 μL of the supernatant was collected, the methanol content in the solution was diluted to 53% using mass spectrometry-grade water (Merck, Germany), and the solution was centrifuged at 15,000 g and 4°C for 20 min, after which the supernatant was collected for analysis. Liquid chromatography–mass spectrometry (LC-MS) analysis was performed (Want et al., 2013) according to the following chromatographic conditions: the chromatograph used was the Vanquish UHPLC (Thermo Fisher, Germany), the chromatographic column was a Hypesil Gold column (100×2.1 mm, 1.9 μm) (Thermo Fisher, USA), the column temperature was set to 40°C, and the flow rate was set to 0.2 mL min^{-1}. Positive ion mode: mobile phase A: 0.1% formic acid, mobile phase B: methanol. Negative ion mode: mobile phase A: 5 mM ammonium acetate at pH 9.0, mobile phase B: methanol. The chromatographic gradient elution program was as follows: 0 min: A: 98%, B: 2%; 1.5 min: A: 98%, B: 2%; 12 min: B: 100%; 14 min: B: 100%; 14.1 min: A: 98%, B: 2%; 17 min: A: 98%, B: 2%. The mass spectrometry conditions were as follows: mass spectrometer: Q Exactive™ HF-X (Thermo Fisher, Germany), with a selected scanning range of m/z 100–1500. The settings for the ESI source were as follows: spray voltage 3.2 kV; sheath gas flow rate 40

arb; auxiliary gas flow rate 10 arb; capillary temperature 320°C; funnel RF level 40; auxiliary gas heater temperature 350°C; polarity positive and negative; and the MS/MS secondary scans were data-dependent scans. After the measurement was completed, CD 3.1 library search software was employed to screen each metabolite in the original offline data to determine the retention time, mass-to-charge ratio, and other parameters, and a retention time deviation of 0.2 min and a mass deviation of 5 ppm were set to compare different samples. Subsequently, the peaks were extracted and the peak areas were quantified, and then the target ions were integrated to predict the molecular formula based on the molecular ion peaks and fragment ions. Each metabolite was compared with the mzCloud (https://www.mzcloud.org/), mzVault and Masslist databases. After using blank samples to remove background ions, the original quantitative results were standardized, and finally the metabolites identification and relative quantification results were obtained.

Supplementary Method 7. In-vitro chemotaxis and biofilm formation assays

To explore which metabolites screened in CC20 stimulated the growth of MCC16, *in-vitro* chemotaxis and biofilm formation experiments were performed. The chemotaxis experiment was slightly modified according to a previously reported method (Rudrappa et al., 2008). Briefly, the MCC16 seed solution was first activated, diluted to a ratio of 1%, and then inoculated into fresh LB broth. Incubation was performed at a constant shaking speed of 170 r min^{-1} until OD_{600}=1, followed by centrifugation for 5 min (4°C, 5000 r min^{-1}).The solution was resuspended in an equal volume of chemotaxis buffer (100 mM K_3PO_4 (pH 7.0) and 20 μM EDTA) (Xiong et al., 2020), and a pipette was used to collect 100 μL of MCC16 bacterial suspension for later use. A 1-mL sterile syringe was employed to accurately draw 100 μL of 11 metabolites (N-isovalerylglycine, epimedin C, D-turanose, nobiletin, sauchinone, praeruptorin A, isomucronulatol-7-O-glucoside, methyl 4-hydroxy-3-methoxycinnamate, 3-succinoylpyridine, thioctic acid and kaempferol) at different concentrations (0, 10, 20, 40, 60, 80, and 100 μg mL^{-1}), after which the pipette containing MCC16 and the syringe containing metabolites were placed horizontally on a clean bench within a laminar flow hood with the pipette tip on the right and the syringe needle left. The needle was inserted into the pipette tip for 2 h. After 2 h, the syringe was carefully removed, the solution was spread over LB solid medium, and the plate dilutions were counted. Each treatment was repeated three times using chemotaxis buffer as a control. The number of colonies (CFUs) was counted and calculating the chemotaxis index (RCI) was calculated, which is the ratio of the number of treated colonies to the number of control colonies (when RCI>2, it is considered to have a significant difference).

The effects of metabolites on the biofilm-forming properties of MCC16 were determined

based on a previously described experimental method (Tan et al., 2013) with slight modifications. Briefly, the MCC16 seed liquid was first activated, then diluted to a ratio of 1% and inoculated into fresh LB broth. The solution was cultivated at a constant temperature of 37°C and 170 r min^{-1} until OD_{600}=0.2, then diluted 100 times, and we added 10 μL of metabolites per milliliter of bacterial suspension to transfer different concentrations (0, 10, 20, 40, 60, 80, and 100 μmol L^{-1}) of nobiletin into the bacterial suspension. The solutions were mixed thoroughly and transferred to a 96-well cell culture plate with 200 μL per well. Each treatment was repeated for eight times and incubated statically at 37°C for 24 h. The bacterial solution without the test substance was used as a control. After incubation, we carefully removed the culture medium under the biofilm was carefully removed from each well and the biofilm was gently rinsed three times with 250 μL of sterile physiological saline. After drying at room temperature, 200 μL of 0.1% crystal violet solution was added and let to stand for 30 min. The dye solution was discarded and then carefully rinsed four times with 250 μL of sterile saline and dried at room temperature. Following drying, 200 μL of 95% ethanol was added for decolorization, mixed, and diluted 40 times, and the absorbance was measured at a wavelength of 562 nm using a microplate reader (Bio-Rad, Microplate Reader 550, Hercules, CA, USA).

Supplementary Method 8. Effects of exogenous apply nobiletin on adventitious root (AR) numbers of potato seedlings in sterlized continuous cropping soil

First, starting with CC soil as the background, the soil was divided into two portions. One portion underwent sterilization treatment, while the other was kept in its natural, unsterilized state. Next, an exogenous nobiletin solution was applied to both the sterilized and unsterilized soils, with equal amount of sterile water serving as the control. Ten pots were set up for each treatment, and each pot (300 mm upper diameter × 210 mm height) was loaded with 4 kg soil and planted with 1 seed potato, and potato seedlings were irrigated with 100 mL of nobiletin solution (20 μM) at 3, 6, and 9 DAE. At 10 and 20 DAE, potato plants were collected to determine AR numbers.

Supplementary Figures

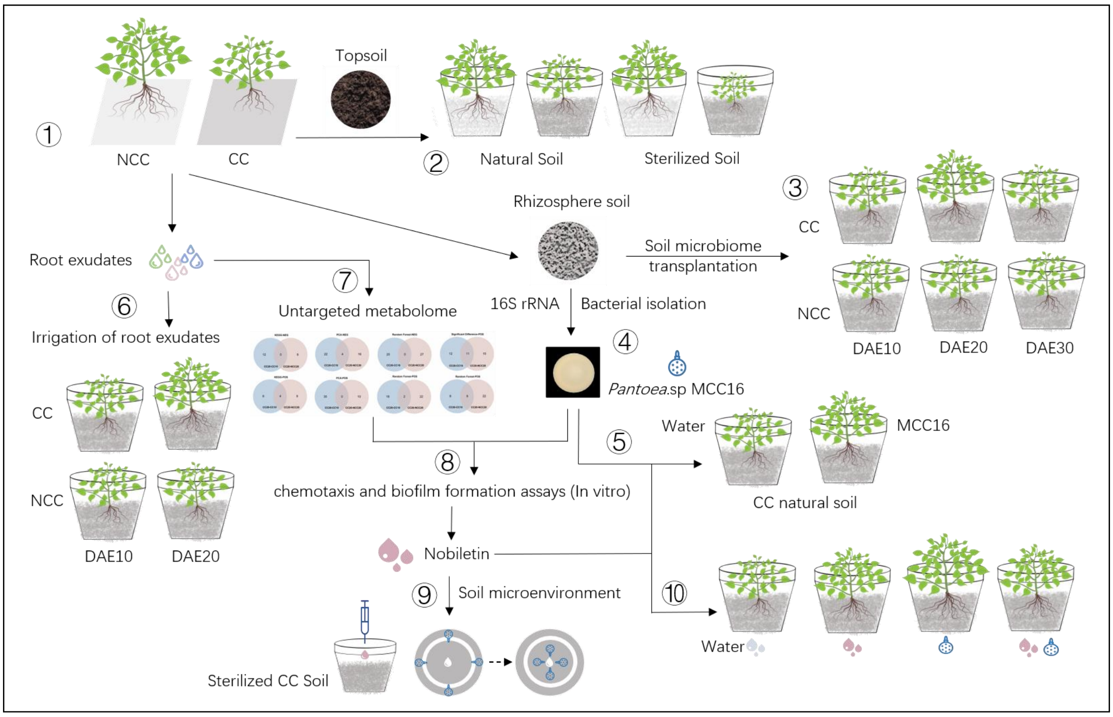

Supplementary Figure 1. Schematic representation of the experimental design.

First, compared with noncontinuous cropping (NCC), the inhibitory effect of continuous cropping (CC) on potato growth was verified ①. Then, field topsoil was collected and sterilized to determine whether soil microorganisms under CC promoted plant growth ②. Rhizosphere soil of potato seedlings was collected in the field for bacterial transplantation to observe when (DAE, days after potato emergence) soil microorganisms under CC promoted plant growth ③. Subsequently, the target strain *Pantoea* sp. MCC16 was isolated by 16S rRNA absolute quantitative sequencing and pure culture ④, and then, the strain was applied to soil to verify its promotion of growth of CC potatoes ⑤. To determine which substances enriched *Pantoea* sp. MCC16 in CC soil, first, root exudates of CC potato seedlings were collected and applied to CC soil to observe the effects on potato plant growth ⑥. Subsequently, metabolites that increased significantly in root exudates of CC potato were screened by nontargeted metabolomics ⑦ and then analyzed with *Pantoea* sp. MCC16 for chemotaxis and biofilm formation ⑧. The metabolite nobiletin, which may recruit *Pantoea* sp. MCC16, was selected. Functional verification of nobiletin to recruit *Pantoea* sp. MCC16 was conducted in a microenvironment experiment ⑨. Last, a pot experiment was used to confirm that exogenous nobiletin and *Pantoea* sp. MCC16 could effectively alleviate the CC barrier of potato ⑩.

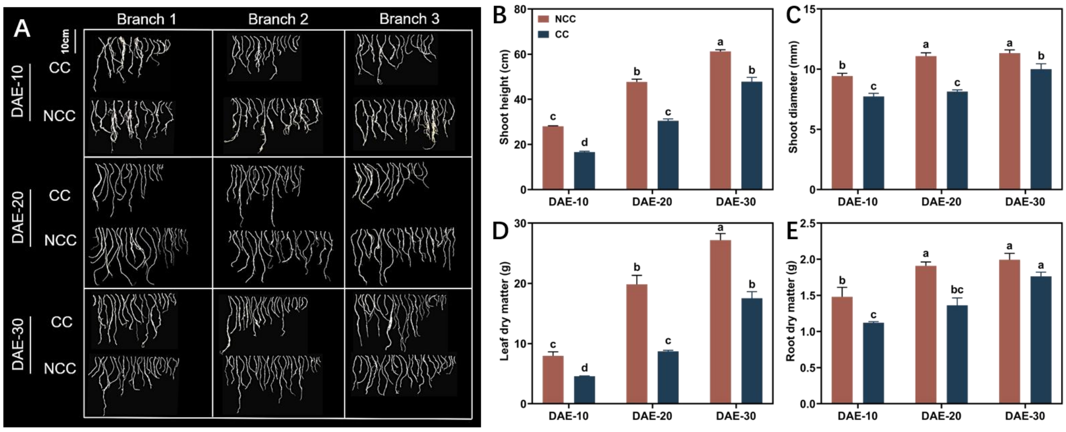

Supplementary Figure 2. Effects of continuous cropping (CC) and noncontinuous cropping (NCC) on potato seedling growth at 10, 20, and 30 days after emergence (DAE).

(A) Effect of continuous cropping (CC) and noncontinous cropping (NCC) on the number of adventitious roots (scale bar = 10 cm). Each plant contains three branches.

(B) Effects of CC and NCC on potato plant height.

(C) Effects of CC and NCC on potato shoot diameter.

(D) Effects of CC and NCC on potato leaf dry matter.

(E) Effects of CC and NCC on potato root dry matter.

Data are shown as the mean ± SEM (n = 3). Different letters indicate significant differences (Tukey's HSD test, $p < 0.05$).

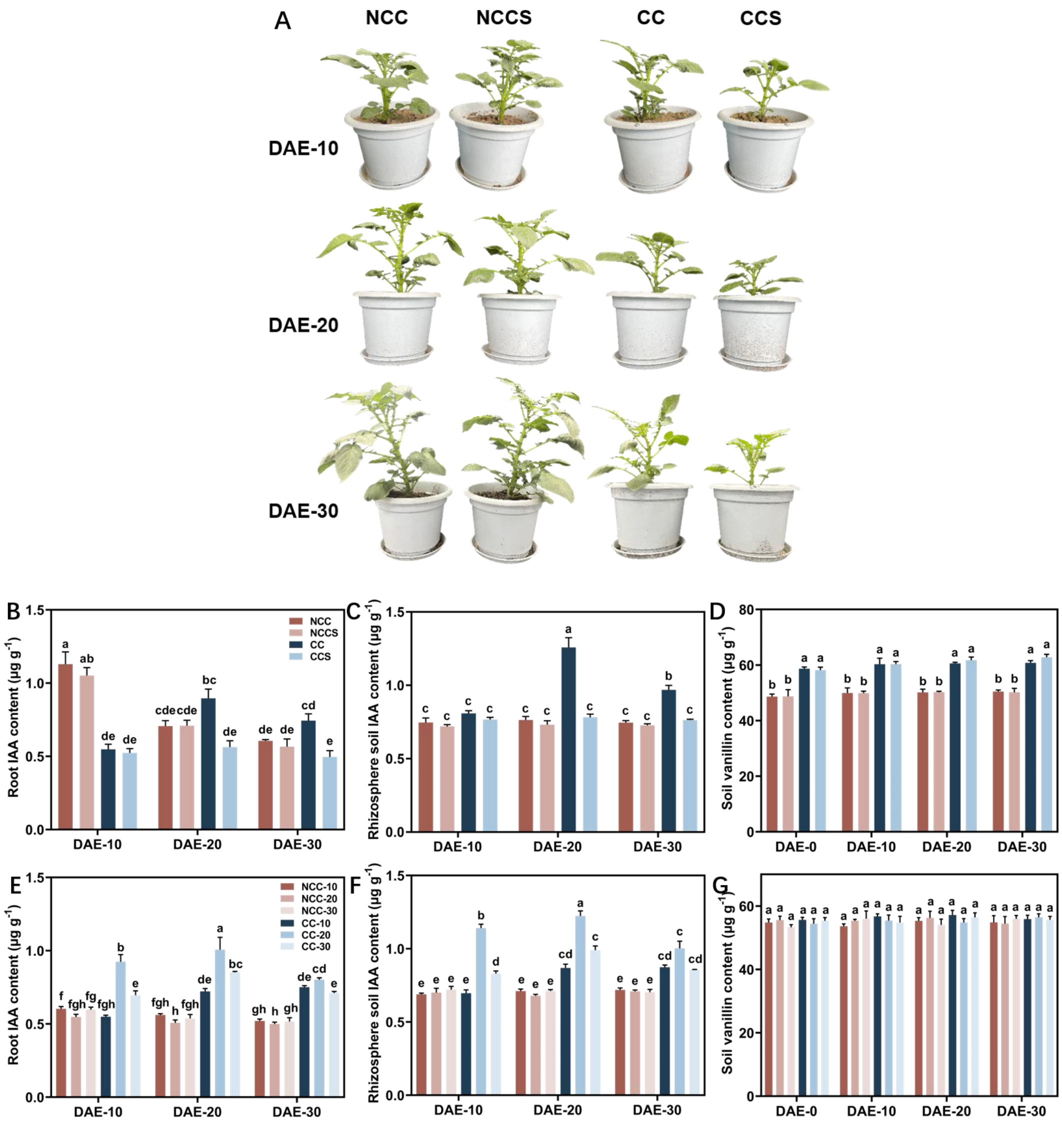

Supplementary Figure 3. Effects of soil sterilization and transplantation of continuous cropping soil microorganisms on potato plant growth.

(A) Effect of soil sterilization on potato plant growth.

(B) Effect of soil sterilization on root IAA content.

(C) Effect of soil sterilization on rhizosphere soil IAA content.

(D) Effect of soil sterilization on soil vanillin content.

(E) Effect of soil microflora transplantation on root IAA content.

(F) Effect of soil microflora transplantation on rhizosphere soil IAA content.

(G) Effect of soil microflora transplantation on soil vanillin content.

NCC: noncontinuous cropping natural soil; NCCS: noncontinuous cropping sterilized soil; CC: continuous cropping natural soil; CCS: continuous cropping sterilized soil. NCC-X: rhizosphere microorganisms on day X after potato emergence in noncontinuous cropping soils; CC-X: rhizosphere microorganisms on day X after potato emergence in continuous cropping soils; DAE-X: X days after potato emergence. IAA: indole-3-acetic acid. Data are shown as the mean ± SEM (n = 3). Different letters indicate significant differences (Tukey's HSD test, p < 0.05).

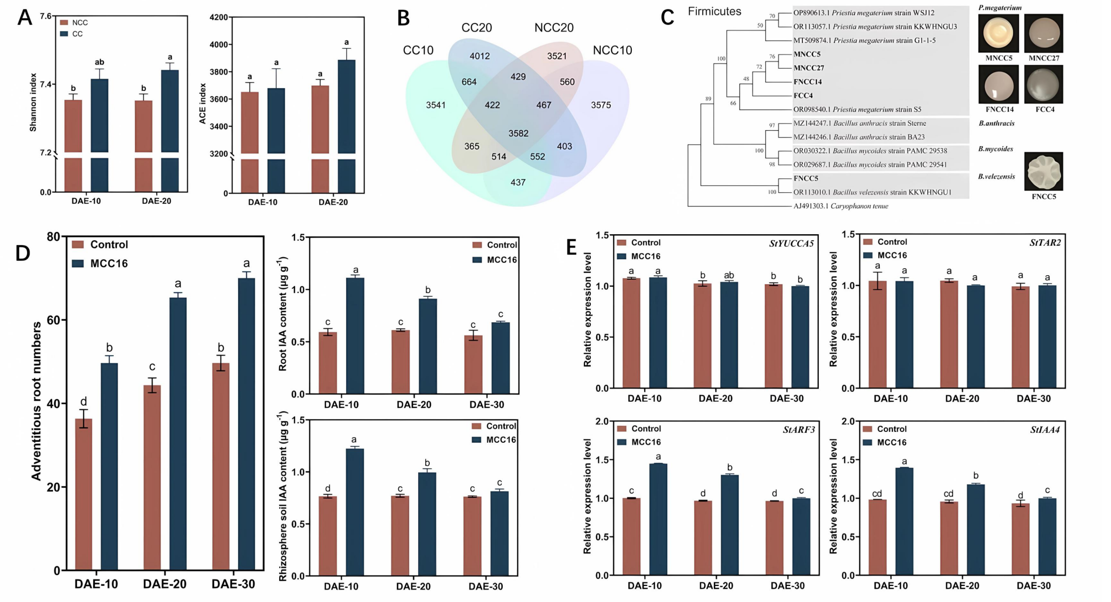

Supplementary Figure 4. Effects of continuous cropping on soil microbial community structure and irrigation with exogenous *Pantoea* sp. MCC16 on the growth of continuous cropping potato plants.

(A) α diversity analysis of the rhizosphere bacterial community structure in CC and NCC potato.

(B) Venn diagram of CC and NCC potato rhizosphere bacterial community diversity. The codes represent the number of amplicon sequence variants (ASVs) detected.

(C) Evolutionary tree analysis of five strains belonging to *Firmicutes*.

(D) Effects of exogenous *Pantoea* sp. MCC16 on the CC potato adventitious root (AR) numbers, root IAA content, and rhizosphere soil IAA content.

(E) Effects of exogenous *Pantoea* sp. MCC16 on the expression of IAA synthesis genes (*StYUCCA5 and StTAR2*) and response genes (*StARF3 and StIAA4*) expression in CC potato roots.

CC: continuous cropping, NCC: noncontinuous cropping. IAA: indole-3-acetic acid. DAE-X: X days after potato emergence. Data are shown as the mean ± SEM (n = 3). Different letters indicate significant differences (Tukey's HSD test, $p < 0.05$).

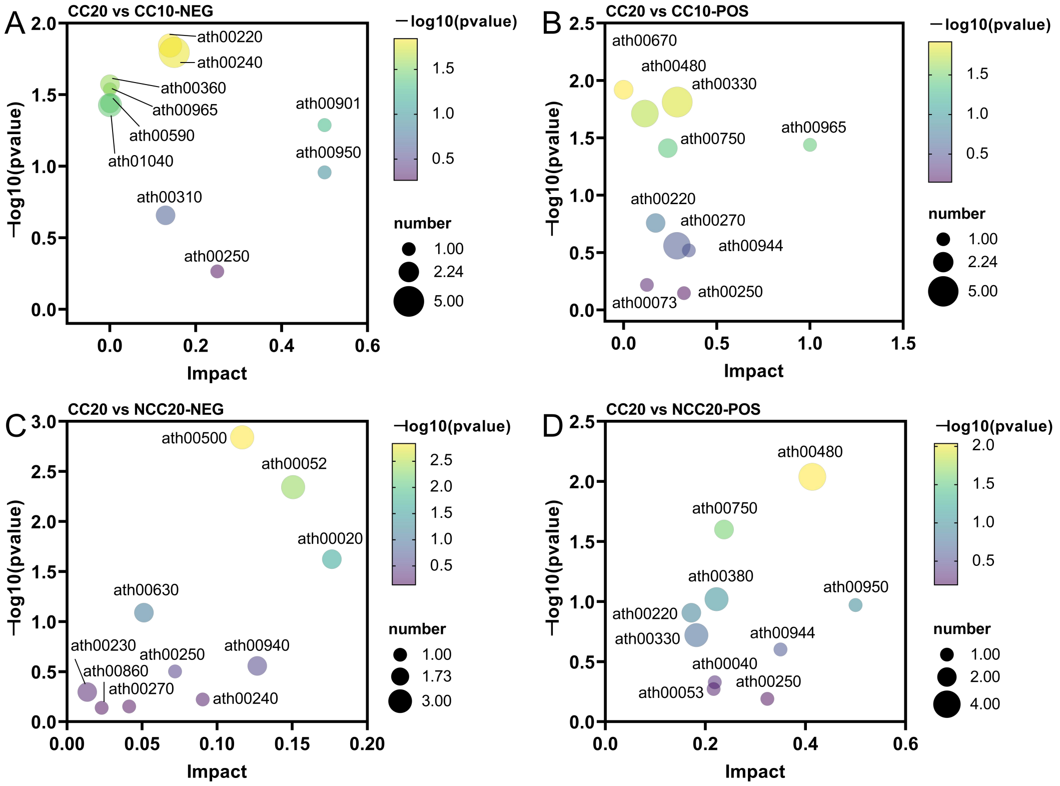

Supplementary Figure 5. Top 10 significantly enriched KEGG (Kyoto Encyclopedia of Genes and Genomes) pathways of differential root metabolites in CC20.

(A) Compared with CC10, the top 10 metabolic pathways demonstrating the significant enrichment of differential metabolites under negative ion mode were detected in the root exudates of CC20.

(B) Compared with CC10, the top 10 metabolic pathways demonstrating the significant enrichment of differential metabolites under positive ion mode were identified in the root exudates of CC20.

(C) Compared with NCC20, the top 10 metabolic pathways demonstrating the significant enrichment of differential metabolites under negative ion mode were detected in the root exudates of CC20.

(D) Compared with NCC20, the top 10 metabolic pathways demonstrating the significant enrichment of differential metabolites under positive ion mode were identified in the root exudates of CC20.

NCC-X: root metabolites on day X after potato emergence in noncontinuous cropping soils; CC-X: root metabolites on day X after potato emergence in continuous cropping soils. NEG: negative ion mode. POS: positive ion mode. Here, “number” represents the amount of significantly different metabolites enriched in this metabolic pathway, and “Impact” represents the contribution degree of difference formation. The more yellow the color, the more significant the difference.

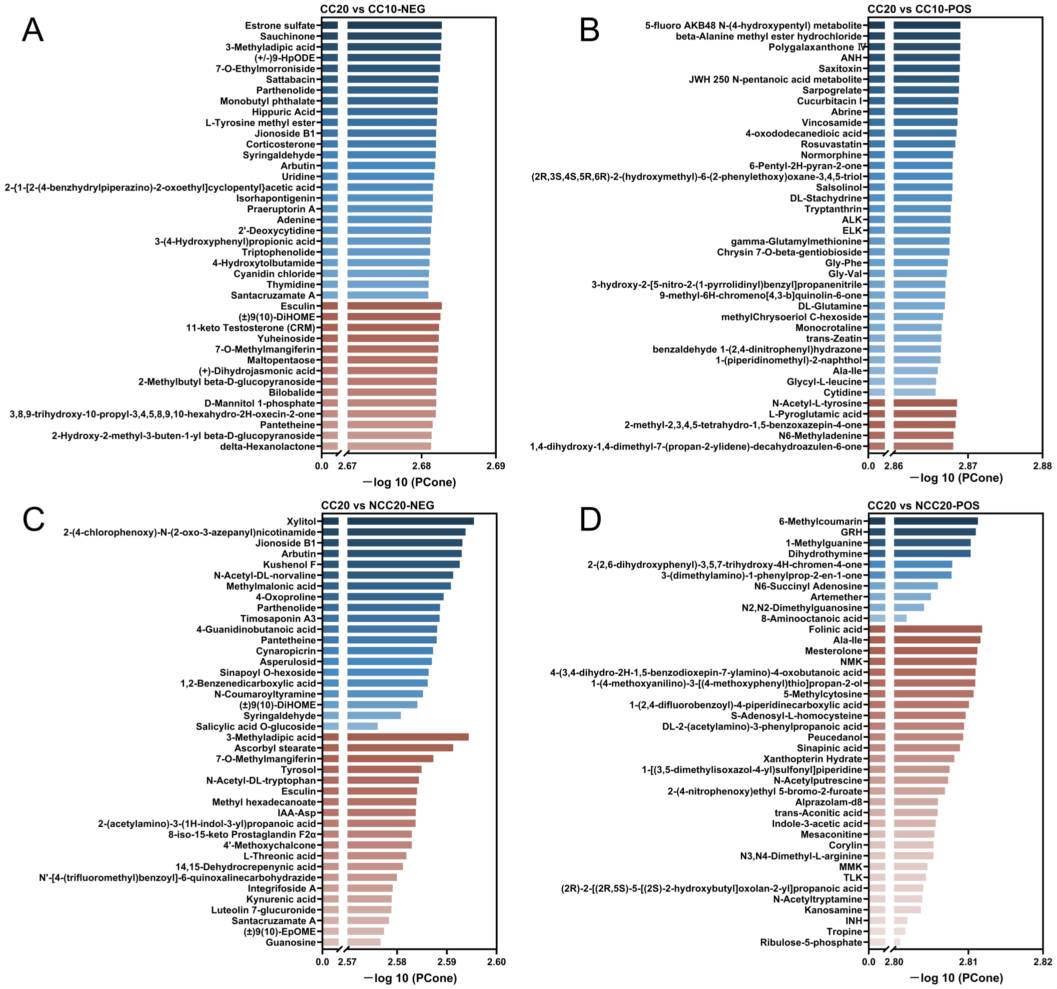

Supplementary Figure 6. Principal component analysis variable importance analysis (TOP40) of differential root metabolites.

(A) Ranking of significantly differential metabolites under negative ion mode in root exudates between CC10 and CC20.

(B) Ranking of significantly differential metabolites under positive ion mode in root exudates between CC10 and CC20.

(C) Ranking of significantly differential metabolites under negative ion mode in root exudates between NCC20 and CC20.

(D) Ranking of significantly differential metabolites under positive ion mode in root exudates between NCC20 and CC20.

NCC-X: root metabolites on day X after potato emergence in noncontinuous cropping soils; CC-X: root metabolites on day X after potato emergence in continuous cropping soils. Blue indicates enrichment in CC20 and red indicates enrichment in CC10 or NCC20, where the darker the color, the greater the importance.

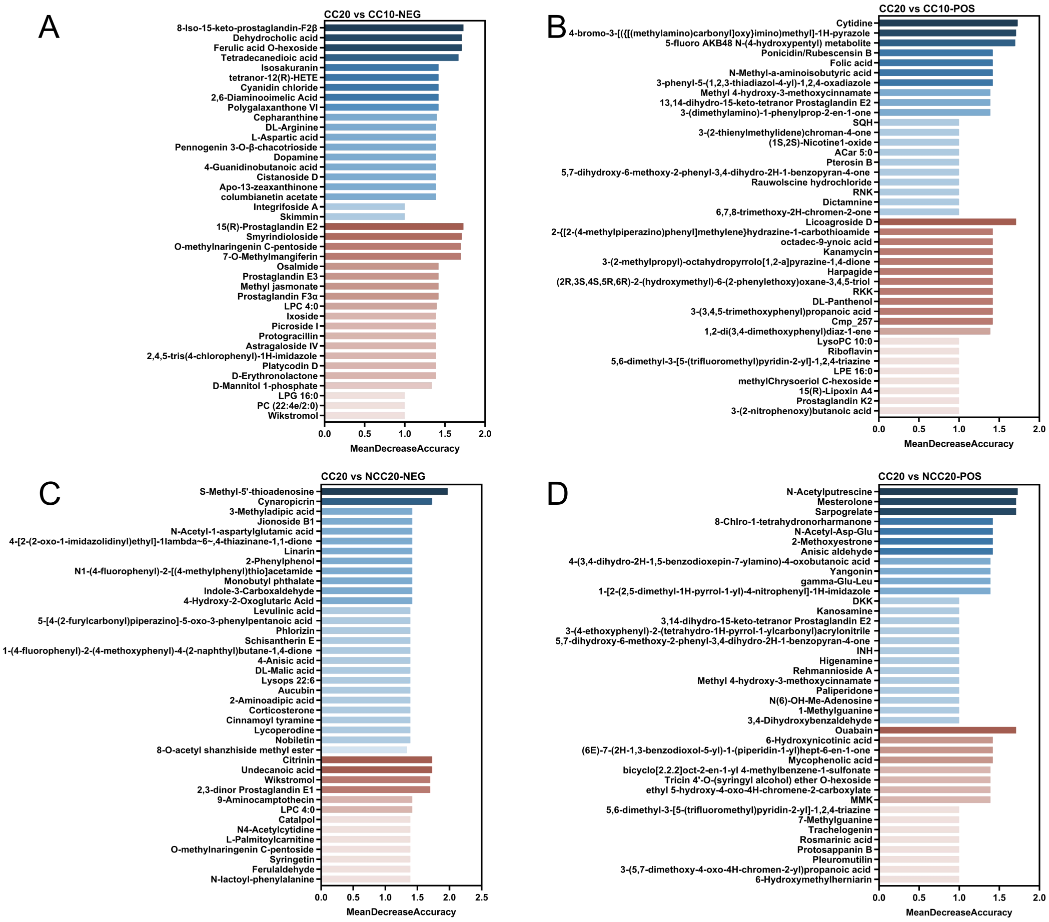

Supplementary Figure 7. Random forest variable importance analysis (TOP40) of differential root metabolites.

(A) Ranking of significantly differential metabolites under negative ion mode in root exudates between CC10 and CC20.

(B) Ranking of significantly differential metabolites under positive ion mode in root exudates between CC10 and CC20.

(C) Ranking of significantly differential metabolites under negative ion mode in root exudates between NCC20 and CC20.

(D) Ranking of significantly differential metabolites under positive ion mode in root exudates between NCC20 and CC20.

NCC-X: root metabolites on day X after potato emergence in noncontinuous cropping soils; CC-X: root metabolites on day X after potato emergence in continuous cropping soils. Blue indicates enrichment in CC20 and red indicates enrichment in CC10 or NCC20, where the darker the color, the greater the importance.

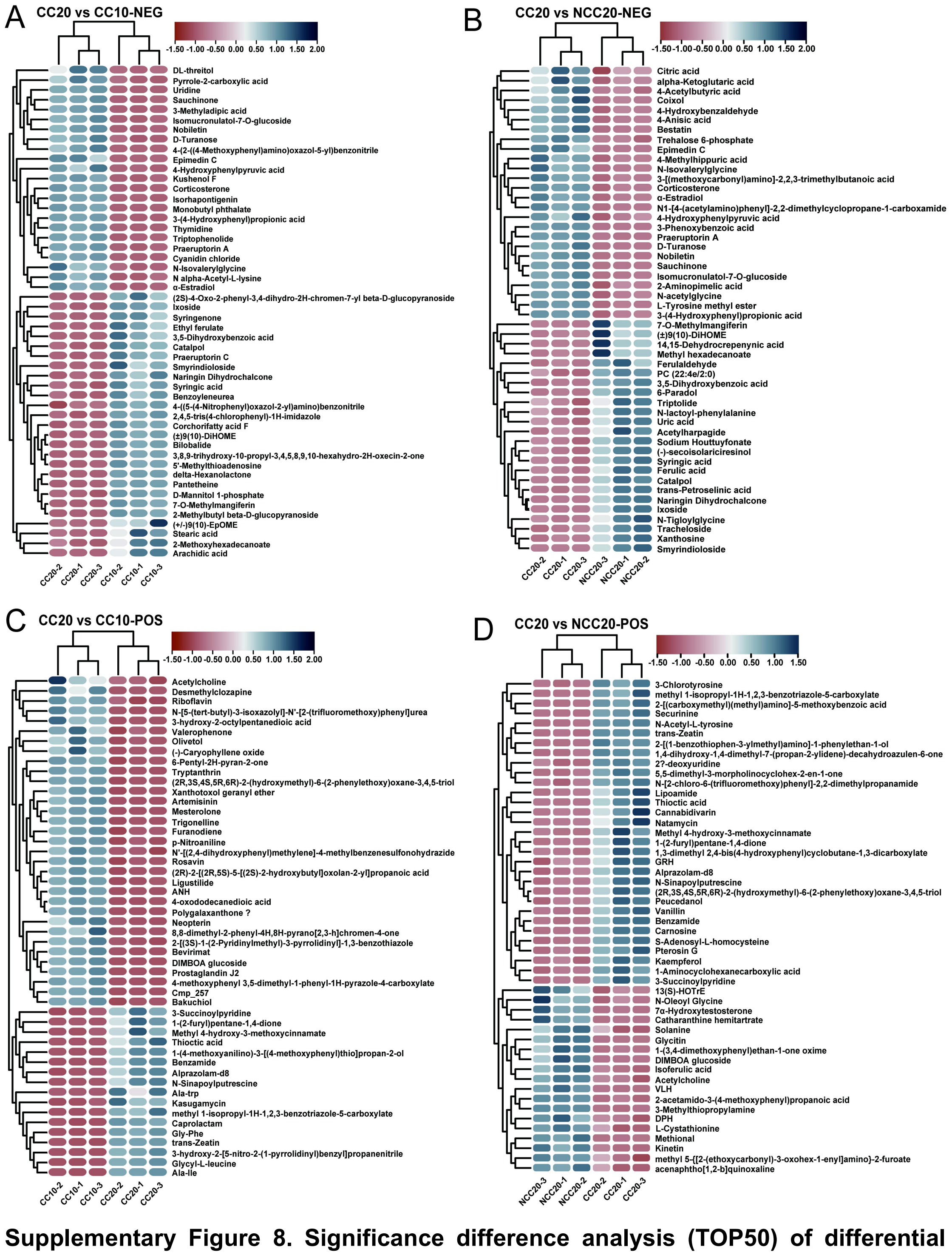

Supplementary Figure 8. Significance difference analysis (TOP50) of differential root metabolites.

NCC-X: root metabolites on day X after potato emergence in noncontinuous cropping soils; CC-X: root metabolites on day X after potato emergence in continuous cropping soils.

(A) Ranking of significantly differential metabolites under negative ion mode in root exudates between CC10 and CC20.

(B) Ranking of significantly differential metabolites under negative ion mode in root exudates between NCC20 and CC20.

(C) Ranking of significantly differential metabolites under positive ion mode in root exudates between CC10 and CC20.

(D) Ranking of significantly differential metabolites under positive ion mode in root exudates between NCC20 and CC20.

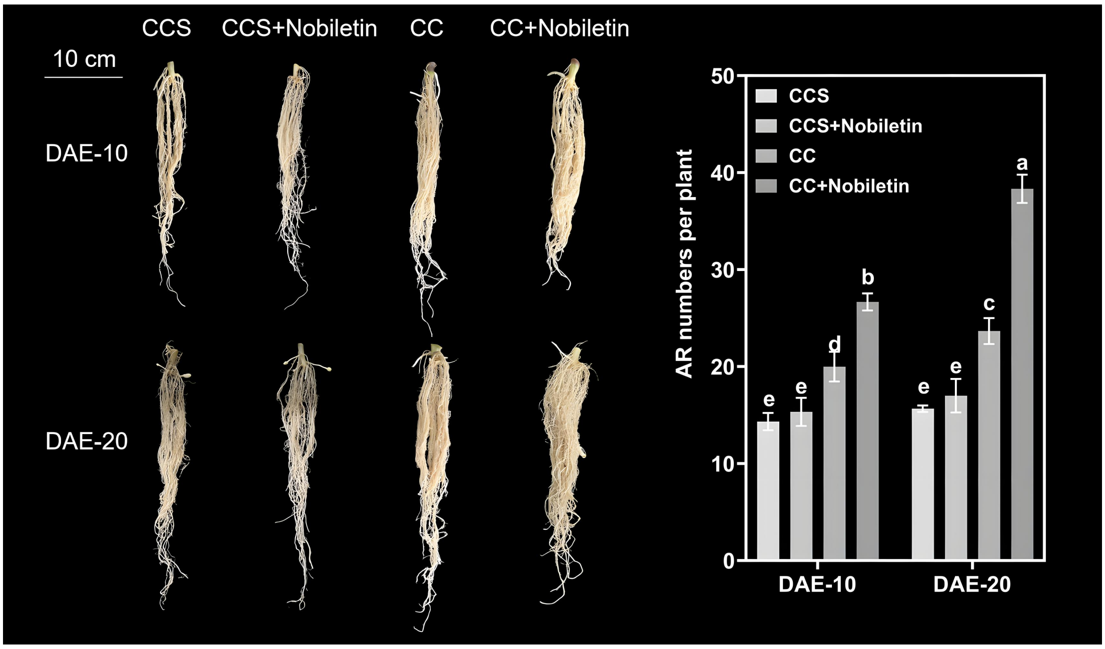

Supplementary Figure 9. Nobiletin enhances adventitious root (AR) numbers of potato seedlings in continuous cropping systems via soil microorganisms

CC:continuous cropping soil; CCS:sterilized:continuous cropping soil; CC+Nobiletin: apply nobiletin to CC soil; CCS+Nobiletin: apply nobiletin to CCS soil.

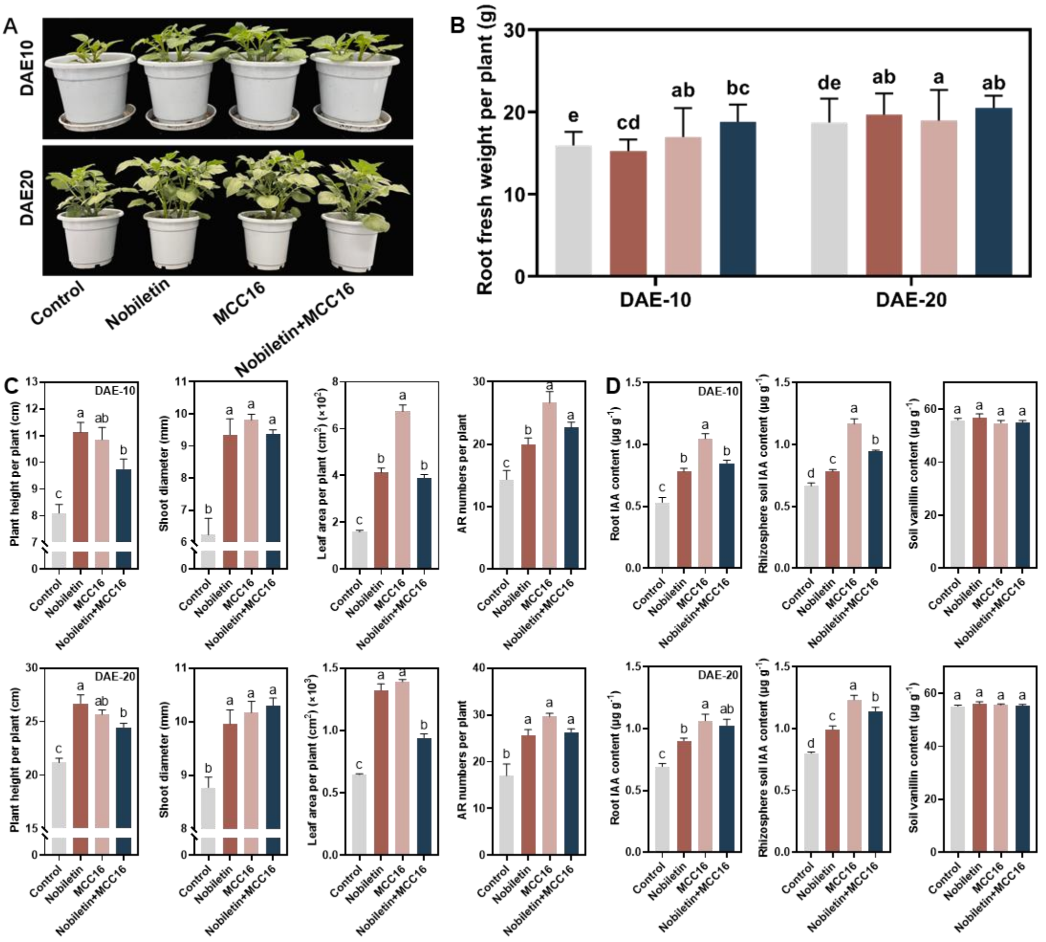

Supplementary Figure 10. Alleviating effects of exogenous nobiletin and *Pantoea* sp. MCC16 on continuous cropping potato.

(A) Effects of exogenous nobiletin or *Pantoea* sp. MCC16 on the growth of continuous cropping potato plants

(B) Effects of exogenous nobiletin or *Pantoea* sp. MCC16 on the root fresh weight of continuous cropping potato plants

(C) Effects of exogenous nobiletin or *Pantoea* sp. MCC16 on the plant height, shoot diameter, leaf area and adventitious root (AR) numbers of continuous cropping potato plants

(D) Effects of exogenous nobiletin or *Pantoea* sp. MCC16 on root IAA content, rhizosphere soil IAA content, and rhizosphere soil vanillin content.

Data are shown as the mean ± SEM (n = 3). Different letters indicate significant differences (Tukey's HSD test, $p < 0.05$).

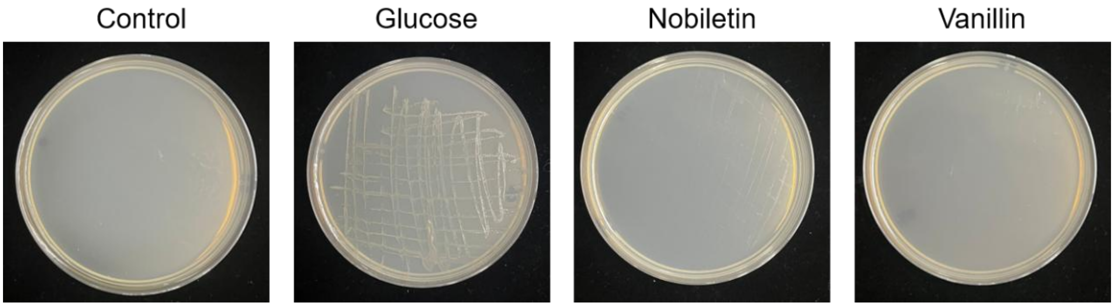

Supplementary Figure 11. Carbon source identification of *Pantoea* sp. MCC16.

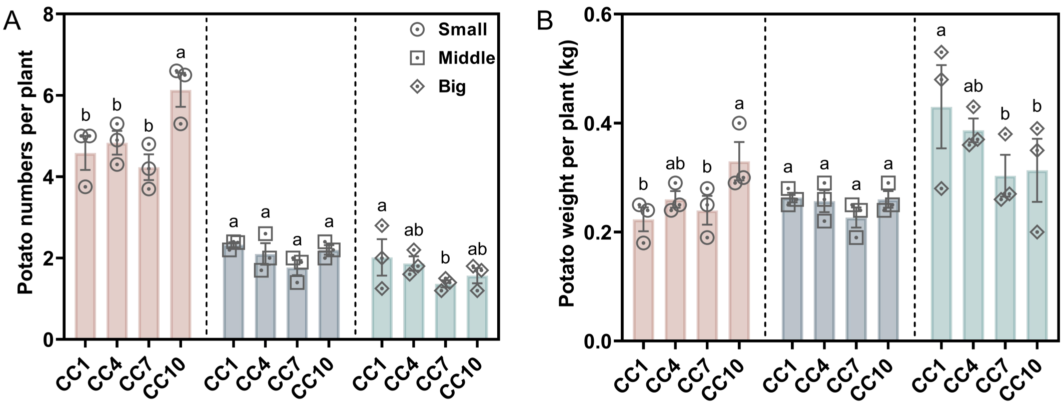

Supplementary Figure 12. Yield composition of continuous cropping potato in the field.

(A) Number of potatoes per plant under different continuous cropping years.

(B) Potato weight per plant under different continuous cropping years.

CC-X: potato continuous cropping for X years. Small: < 80 g, Middle: 80–200 g, Big: > 200 g. Data are shown as the mean ± SEM (n = 3). Different letters indicate significant differences (Tukey's HSD test, $p < 0.05$).

Supplementary Tables

Supplementary Table 1. List of qRT-PCR primers used in this study.

Gene	Primer name and sequence (5´-3´)	PCR protocol
StEF-1α	Forward, CTTGTACACCACGCTAAGGAG<br>Reverse, GTCAATGCAAACCATTCCTTG	95°C for 1 min; followed by 95°C for 10 s, 60°C for 20 s, 40 cycles
StYUCCA5	Forward, TCCATCTTGGCTACAGGAAAGTGA<br>Reverse, TGCAACGTCTGTGTGTTGGAACT	
StTAR2	Forward, GGAGAATGCAACTTCAGCCATCA<br>Reverse, TCACGGAGACTCCACGGAGT	
StARF3	Forward, TGTTGTAGGTATCTCACATCTGGTTCACA<br>Reverse, TCTAGACCAAGTGAGTACACAGCTTCA	
StIAA4	Forward, AGAAGCTGAGTGTGGAATGTATGTGA<br>Reverse, TCTCCAACAAGCATCAAGTCACCA	

Supplementary Table 2 Identification of the IAA production capacity of all rhizosphere soil isolates during the potato seedling stage. " + " and " - " indicate that the color reaction is positive (bolded text) and negative (plain text), respectively.

DAE-10		DAE-20		DAE-30	
Strain ID	IAA-production	Strain ID	IAA-production	Strain ID	IAA-production
INCC1	-	MNCC1	-	FNCC1	-
INCC2	-	MNCC2	-	FNCC2	-
INCC3	-	**MNCC3**	+	FNCC3	-
INCC4	-	MNCC4	-	FNCC4	-
INCC5	-	**MNCC5**	+	**FNCC5**	+
INCC6	-	**MNCC6**	+	FNCC6	-
INCC7	-	MNCC7	-	FNCC7	-
INCC8	-	MNCC8	-	FNCC8	-
**INCC9**	+	MNCC9	-	FNCC9	-
**INCC10**	+	MNCC10	-	FNCC10	-
INCC11	-	MNCC11	-	FNCC11	-
INCC12	-	MNCC12	-	FNCC12	-
ICC1	-	MNCC13	-	FNCC13	-
**ICC2**	+	MNCC14	-	**FNCC14**	+
ICC3	-	MNCC15	-	FNCC15	-
ICC4	-	MNCC16	-	FNCC16	-
		**MNCC17**	+	**FNCC17**	+
		MNCC18	-	**FNCC18**	+
		MNCC19	-	FNCC19	-
		MNCC20	-	FNCC20	-
		MNCC21	-	**FNCC21**	+
		MNCC22	-	FNCC22	-
		MNCC23	-	FCC1	-
		MNCC24	-	FCC2	-

MNCC25	-	FCC3	-
MNCC26	-	**FCC4**	+
**MNCC27**	+	FCC5	-
MNCC28	-	FCC6	-
MNCC29	-	**FCC7**	+
MNCC30	-	FCC8	-
MNCC31	-	FCC9	-
MNCC32	-	FCC10	-
MNCC33	-	**FCC11**	+
MCC1	-	FCC12	-
MCC2	-	FCC13	-
MCC3	-	FCC14	-
MCC4	-	FCC15	-
MCC5	-		
MCC6	-		
MCC7	-		
MCC8	-		
MCC9	-		
MCC10	-		
MCC11	-		
MCC12	-		
MCC13	-		
MCC14	-		
MCC15	-		
**MCC16**	+		
MCC17	-		
MCC18	-		
MCC19	-		

Supplementary Table 3. Root exudates of the top 10 KEGG pathways enriched in CC20.

Treatment	KEGG ID	KEGG pathway	Root exudates enriched in CC20
CC20<br>vs<br>CC10<br>NEG	ath00220	Arginine biosynthesis	Fumaric acid<br>trans-Cinnamic acid<br>L-Aspartic acid<br>Uridine<br>Thymidine<br>L-Saccharopine<br>Phenylacetaldehyde<br>Dopamine<br>Leukotriene C4
	ath00240	Pyrimidine metabolism	
	ath00360	Phenylalanine metabolism	
	ath00965	Betalain biosynthesis	
	ath00590	Arachidonic acid metabolism	
	ath01040	Biosynthesis of unsaturated fatty acids	
	ath00901	Indole alkaloid biosynthesis	

	ath00950	Isoquinoline alkaloid biosynthesis	L-Ornithine<br>2'-Deoxycytidine<br>Tryptamine
	ath00310	Lysine degradation	
	ath00250	Alanine, aspartate and glutamate metabolism	
CC20<br>vs<br>NCC20<br>NEG	ath00500	Starch and sucrose metabolism	Citric acid<br>Sucrose<br>α,α-Trehalose<br>Stachyose<br>Raffinose<br>dUMP<br>Pheophorbide A<br>Trehalose 6-phosphate<br>alpha-Ketoglutaric acid
	ath00052	Galactose metabolism	
	ath00020	Citrate cycle (TCA cycle)	
	ath00940	Phenylpropanoid biosynthesis	
	ath00240	Pyrimidine metabolism	
	ath00250	Alanine, aspartate and glutamate metabolism	
	ath00630	Glyoxylate and dicarboxylate metabolism	
	ath00270	Cysteine and methionine metabolism	
	ath00860	Porphyrin metabolism	
	ath00230	Purine metabolism	
CC20<br>vs<br>CC10<br>POS	ath00670	One carbon pool by folate	**4-Guanidinobutyric acid**<br>**L-Glutamic acid**<br>**L-Pyroglutamic acid**<br>S-Adenosyl-L-homocysteine<br>(5-L-Glutamyl)-L-amino acid<br>L-Homoserine<br>**Kaempferol**<br>Homocysteine<br>Folinic acid<br>Folic acid
	ath00330	Arginine and proline metabolism	
	ath00480	Glutathione metabolism	
	ath00965	Betalain biosynthesis	
	ath00750	Vitamin B6 metabolism	
	ath00944	Flavone and flavonol biosynthesis	
	ath00250	Alanine, aspartate and glutamate metabolism	
	ath00270	Cysteine and methionine metabolism	
	ath00220	Arginine biosynthesis	
	ath00073	Cutin, suberine and wax biosynthesis	
CC20<br>vs<br>NCC20<br>POS	ath00480	Glutathione metabolism	**4-Guanidinobutyric acid**<br>Serotonin<br>**L-Glutamic acid**<br>Pyridoxine<br>Indole-3-acetic acid<br>**L-Pyroglutamic acid**<br>D-Xylulose<br>3-Hydroxyanthranilic acid<br>Pyridoxal<br>**Kaempferol**<br>Glutathione<br>L-Citrulline<br>Vitamin C
	ath00750	Vitamin B6 metabolism	
	ath00950	Isoquinoline alkaloid biosynthesis	
	ath00944	Flavone and flavonol biosynthesis	
	ath00250	Alanine, aspartate and glutamate metabolism	
	ath00380	Tryptophan metabolism	
	ath00040	Pentose and glucuronate interconversions	
	ath00053	Ascorbate and aldarate metabolism	
	ath00330	Arginine and proline metabolism	
	ath00220	Arginine biosynthesis	
